# A Bayesian measure of association that utilizes the underlying distributions of noise and information

**DOI:** 10.1371/journal.pone.0201185

**Published:** 2018-08-17

**Authors:** Ishan Goel, Sukant Khurana

**Affiliations:** CSIR-Central Drug Research Institute, Jankipuram Extension, Lucknow, Uttar Pradesh, India; University of North Carolina at Chapel Hill, UNITED STATES

## Abstract

We propose a new approach, Bayesian Probability of Association (BPA) which takes into account the probability distributions of information and noise in the variables and uses Bayesian statistics to predict associations better than existing approaches. Our approach overcomes the limitations of linearity of the relationship and normality of the data, assumed by the Pearson correlation coefficient. It is different from the current measures of association because considering information separately from noise helps identify the association in information more accurately, makes the approach less sensitive to noise and also helps identify causal directions. We tested the approach on 15 datasets with no underlying association and on 75 datasets with known causal relationships and compared the results with other measures of association. No false associations were detected and true associations were predicted in more than 90% cases whereas the Pearson correlation coefficient and mutual information content predicted associations for less than half of the datasets.

## Introduction

Studying associations between variable is essential to almost all the branches of science and helps us understand the working and the design of a system. In neuroscience, studying associations between different brain regions helps us understand which brain regions are connected [1,2]. In financial markets, studying associations helps us understand which financial instruments are related [3–5]. In social sciences, studying associations helps us understand which factors in a society are associated to one another [6]. Associations are present in all branches of science and understanding such associations help us take decisions, create new designs and predict outcomes.

To study associations among variables, various quantitative approaches have been developed largely around the ideas of correlation analysis and information theory. Correlation analysis between two variables has been central to the study of associations and Pearson Correlation Coefficient has been an indispensable tool for it [7]. Pearson Correlation Coefficient quantifies the extent of linear association in two variables. In other words, Pearson correlation coefficient tests how tightly the scatter plot of two variables fits a line. The tightness of fit is a good measure of association as it suggests that the variables are dependent on each other and when one is changed the other also changes. However, Pearson Correlation Coefficient suffers from the limitations of working only under the assumptions of normality of the underlying data and the linearity of the underlying association [8,9]. Further, attempts have also been made to compare the correlation coefficient with regression and identify the differences in the two [7,10]. Other types of measures have been developed to overcome the limitations of the same. Rank Correlation measures tackle the problem of normality by ranking the data and hence utilize only the order of the values instead of the actual values [9]. Spearman Rank Correlation Coefficient and Kendall Tau Rank Correlation Coefficient are two popular rank correlation coefficients. Other variations of the Pearson correlation coefficient like the circular correlation coefficient [11] and the scaled correlation coefficient [12] were also developed but on the same lines as the Pearson correlation coefficient. Circular correlation coefficient attempted to identify associations in angular values for instance in wind and ocean current directions. Scaled correlation coefficient looks at correlation over smaller time frames to identify the correlations in fast signal components. Further, polyserial and polychoric correlation coefficients also were developed to be robust to the non-normality of data, but they were developed to be used with variables that had a normally distributed underlying latent variable [13]. For instance, Likert scales are often used to measure normally distributed variables that cannot be measured otherwise [14].

Pearson Correlation Coefficient gives both false positives and false negatives because of its limitations of assumptions of linearity and normality. One type of the case where Pearson Correlation Coefficient gives a false negative is the case of testing a quadratic relationship. As the underlying data does not fit a straight line in this case, no matter how tight the relationship is, Pearson Correlation Coefficient is not very high. Further, as Pearson Correlation Coefficient assumes the underlying data to be normally distributed it gives more weight to values away from the mean. When the actual underlying distribution is not normal, Pearson Correlation Coefficient often ends up giving false positives if only the extreme values are correlated. Overcoming one part of the limitation, the distance correlation measures [15] guaranteed independence if the coefficient was 0 and hence theoretically ensured no false negatives.

Pearson Correlation Coefficient is calculated on data arranged into pairs and correlation was checked between two or more groups. However, a different measure was also required for looking at correlation within different groups. Intraclass correlation coefficient was developed which would test the similarity within different groups of data [16]. While these approaches were trying to improve Pearson Correlation Coefficient for specific cases, none of these approaches actually looked at the underlying probability distribution of data.

With the development of Shannon’s information theory [17], attempts were made to look at association by considering the distribution of the considered variables. Mutual Information Content compared the joint probability distribution of two variables with the product of their independent probability distribution to come up with a measure of association [18]. Mutual Information Content overcame the limitations of linearity and also normality but still did not consider information and noise separately. While mutual information content had a range from 0 to ∞, it was adjusted to be in the range of 0 to 1 and the adjusted variant was called the normalized mutual information content so that the quantification of the association could be brought to a standard scale. Other measures like the correlation ratio [19] were also developed that could detect functional dependencies. While information theoretic approaches have given a new direction to associational analysis, they do not look at information and noise separately in the variable and have not provided a major improvement in the accuracy of results.

In our work to identify associations, we use the word noise to refer to the unassociated component in two variables. While this component may not always be undesirable in the data, for the purpose of identifying association between the two variables, this component can be considered noise. Current works on association are highly sensitive to noise in the variables because they do not segregate information and noise. A fundamentally different approach to association is required that takes noise into account and is more robust. In some systems where different variables affect each other very strongly the amount of stochasticity in the system becomes very high. Because of this reason, it is not very easy to identify associations in these systems. One might argue that even if such associations are identified, such variables cannot be expected to behave in a strongly correlated manner due to the high noise. However, identifying associations is always beneficial for the study of the underlying system.

The underlying distribution of the associated component in two variables (which we refer to as information) holds important information for suggesting associations in the variables but has not been fully explored. Correlated values with lower probability are stronger indicators of association as compared to the values with higher probability. The low probability of independent occurrence of two rare events as compared to one being influenced with the other holds information that has not yet been utilized to study associations.

We first present the new approach to test associations and then suggest ways to make good approximations of the underlying distributions and the underlying relationship of the two variables. We then present the result with synthetic and real world data and show how the suggested approach works well even without any information of the underlying distribution and approximating it to be normal. We also suggest an extension of this approach which can potentially help identify causal directions in variables. Lastly, we show how the approach works well with even non-linear data.

## Methods

We have developed a new approach to study associations in data and we present the derivation of the new approach and also explain how should the approach be used. We implemented the approach in Python 3.5 to test the results of the new approach. We have shared our implementation (S1 File) in pdf format and interested readers can copy the functions and use the code.

### Calculating the Bayesian Probability of association

The data from two variables X and Y for n data points is given. As most real world variables are affected by multiple surrounding variables, they always have a noise component in them. Hence, both X and Y are assumed to be the sum of an information component (I) and a noise component (N). Considering the given variable to be a sum of information and a noise component leaves room for an independent component in the two variables and allows for the consideration of the dependent component to be perfectly associated. When the data from the two variables does not fit a curve perfectly, this noise component allows us to look at the possibility of association probabilistically rather than rejecting it straight away.

$$X=I_{X}+N_{X}$$ (1)

$$Y=I_{Y}+N_{Y}$$ (2)

We take two mutually exclusive hypotheses, first that the information components are related by a relationship *f* and second that the information components are independent. Hence, the following hypotheses are taken.

$$H_{1}:I_{X}=f(I_{Y})$$ (3)

$$H_{2}:I_{X}\neq f(I_{Y})$$ (4)

for some function *f* which we refer to as the relationship function. Please note that the first hypothesis assumes that one of the variables is generated stochastically while the other variable assumes its value as a direct consequence of the value of the first variable. For instance, consider the dataset of the sides and areas of squares that are independent and stochastically generated. Observe how, one of the variables can be taken to be stochastically distributed but the other is a direct consequence of the first. Also note that the given coefficient predicts or rejects association considering the specific relationship function and various choices of the same can be taken iteratively to test association. It must be noted here that although *H*_2_ assumes *I*_*X*_ and *I*_*Y*_ to be independent for any relationship function, the suggested approach calculates the probability of association for a single relationship function and hence *P*(*H*_2_) is not the absolute probability of *I*_*X*_ and *I*_*Y*_ being independent. *P*(*H*_2_) is the conditional probability of independence given that the relationship function is *f*. The prior probabilities of 0.5 are assigned to both the hypotheses as there is no reason to believe the likelihood of one over the other until we start observing the data. If there is prior information available, these probabilities can be adjusted accordingly.

$$P(H_{1})=0.5$$ (5)

$$P(H_{2})=0.5$$ (6)

Also note that as the hypotheses are mutually exclusive and exhaustive given the relationship function, the sum of the probabilities of both the hypotheses should be 1 at all times.

$$P(H_{1})+P(H_{2})=1$$ (7)

Now, both the variables for one data point at a time are observed and the likelihood of observing that data point given *H*_1_ and the likelihood of observing the data point given *H*_2_ is calculated. The prior probabilities are updated using Bayesian updating. The posterior probability becomes the prior probability for the next data point and the algorithm is iterated over all data points.

Given both the variables for one data point, say *x* and *y*, the likelihood of observing the values given hypothesis 1 is calculated as

$$L(x,y|H_{1})=\left[0.5*\int_{i=-\infty }^{i=\infty }P(I_{X}=i)*P(N_{X}=x-i)*P(N_{Y}=y-f(i))\right]+\left[0.5*\int_{i=-\infty }^{i=\infty }P(I_{Y}=i)*P(N_{X}=x-f^{-1}(i))*P(N_{Y}=y-i)\right]$$ (8)

where P(e) refers to the probability of some event e and the probabilities will be given by the estimated underlying distribution of the variable. We explain in the next section on how to estimate these distributions. In the first bracket, we consider *I*_*X*_ to be the independent variable and *I*_*Y*_ to be the dependent variable. If the variable, *I*_*X*_ had assumed a value of *i*, *N*_*X*_ must have assumed a value of *x* − *i* and *N*_*Y*_ must have assumed a value of *y* − *f*(*i*). However, this value *i* could have been anything in the range of −∞ to ∞. Hence, we integrate over the range. Please note that *P*(*I*_*Y*_ = *f*(*i*)|*I*_*X*_ = *i*) = 1 as it is a direct consequence of the condition. Also note that all the other probabilities in the equation are independent of each other. There is an equal chance that *I*_*Y*_ was the independent variable and *I*_*X*_ was the dependent variable. Hence, we calculate the same considering the latter case which constitutes the second bracket and take an average of both the values by multiplying them by 0.5 and adding them.

We calculate the likelihood of observing *x* and *y*, given the second hypothesis as follows.

$$L(x,y|H_{2})=\int_{i_{x}=-\infty }^{i_{x}=\infty }P(I_{X}=i_{x})*P(N_{X}=x-i_{x})*\int_{i_{y}=-\infty }^{i_{y}=\infty }P(I_{Y}=i_{y})*P(N_{Y}=y-i_{y})$$ (9)

Considering the second hypothesis, *I*_*X*_ and *I*_*Y*_ must have been generated independently as they have no underlying association. Hence, in the first integral we multiply the probability of *I*_*X*_ assuming a value of *i*_*x*_ and *N*_*X*_ assuming the value of *x* − *i*_*x*_. We integrate this over all possible values of *i*_*x*_. Similarly, we calculate the same for variable Y and multiply the two probabilities.

We then update the probabilities using the Bayesian approach as follows.

$$Posterior(H_{1})=\frac{Prior(H_{1})*L(x,y|H_{1})}{Prior(H_{1})*L(x,y|H_{1})+Prior(H_{2})*L(x,y|H_{2})}$$ (10)

$$Posterior(H_{2})=\frac{Prior(H_{2})*L(x,y|H_{2})}{Prior(H_{1})*L(x,y|H_{1})+Prior(H_{2})*L(x,y|H_{2})}$$ (11)

where Posterior refers to the posterior probability and Prior refers to the prior probabilities.

Observe how the given approach gives more weight to less probable outcomes only when they are encountered in both the variables. Assume that the relationship function *f* is the identity function and *I*_*X*_ = *I*_*Y*_. When *x* and *y*, are encountered away from the mean but close to one another (thus leaving a small residue for the noise variable) *L*(*x*,*y*|*H*_1_) multiplies the low probability of encountering *x* and *y* only once while, *L*(*x*,*y*|*H*_2_) multiplies the low probability twice thus in effect making *L*(*x*,*y*|*H*_1_) higher than *L*(*x*,*y*|*H*_2_). Whereas when *x* and *y*, are encountered away from the mean but away from each other (away from the mean in the opposite directions, thus making the noise component large), the value of noise is also away from the mean in *L*(*x*,*y*|*H*_1_), thus multiplying a low probability component and reducing the overall value. Also observe, when *x* and *y* are not close enough *L*(*x*,*y*|*H*_1_) is reduced because the absolute sum of the two noise components is always equal to |*x* − *y*| (given our relationship function *f* is the identity function, as *I*_*X*_ = *I*_*Y*_, |*N*_*X*_ + *N*_*Y*_| = |x − y|) where as it is not so for *L*(*x*,*y*|*H*_2_).

Note that in the rest of the paper, whenever we present and talk about results, we only present the probability of association which is the probability of the first hypothesis and refer to it as the Bayesian Probability of Association (BPA).

### Estimation of underlying distributions and the relationship functions

Having the dataset, one cannot exactly know the underlying distribution of noise and information but one can make good approximations on the same. In specific studies where one has more domain knowledge of the underlying system, one can make more accurate estimations and have better results.

The central limit theorem states that the sum of different random variables tend to be normally distributed and hence, the normal distribution is a good estimate if nothing else is known about the data. It is worth noting that while Pearson Correlation Coefficient assumes its data to be normally distributed and quantifies association accordingly, BPA is not restricted to be used with the normal distribution and using more accurate distributions will only improve the accuracy of the result. We show with the real world data that this approximation works quite efficiently. When the variable is considered to be normally distributed and the variable *X*, is the sum of *I*_*X*_ and *N*_*X*_, it is known that.

$$Mean(X)=Mean(I_{X})+Mean(N_{X})$$ (12)

$$Variance(X)=Variance(I_{X})+Variance(N_{X})$$ (13)

The mean of the distribution decides the location of the distribution and addition or subtraction of the mean does not change the shape of the distribution. To avoid estimating the actual mean and variance of all the four distributions, we suggest normalizing both the variables before calculating BPA.

Variance of *I*_*X*_ and *N*_*X*_ are indicative of the amount of information and noise in the variable respectively. As the total variance after normalization is 1, a proportion, $\alpha_{X}^{\prime }$ can be estimated to be the variance of information and $\left(1-\alpha_{X}^{\prime }\right)$ can be estimated to be the variance to noise. Various combinations of $\alpha_{X}^{\prime }$ and $\alpha_{Y}^{\prime }$ can be tested to detect associations. Note that in this manuscript we will refer to the actual proportions of variance to be *α*_*X*_ and *α*_*Y*_.

A good approximation to the relationship function can be found by curve fitting approaches and the domain knowledge of the variables being tested. For instance, consider the example consisting of various squares. While the sides have a dimension of length, area has a dimension of length square. This clearly suggests that the underlying relationship function is quadratic in nature.

## Results

We tested the approach on 64 associated synthetic datasets, 64 unassociated synthetic datasets, 75 associated real world datasets, 15 unassociated real world datasets and five synthetic datasets with non-linear relationships. Interestingly, Bayesian Probability of Association was found to perform better than existing approaches in most datasets.

**Fig 1:**
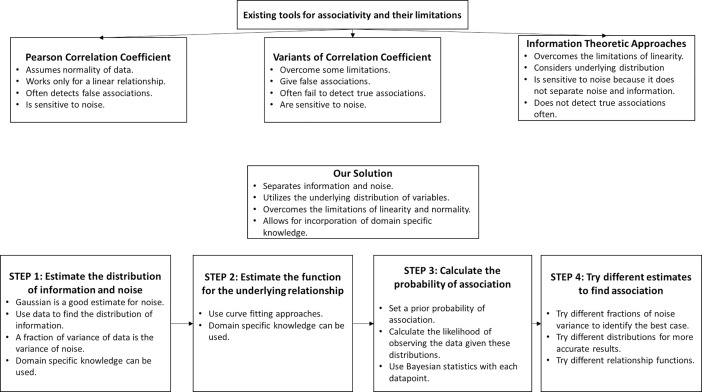
Summarization of the the existing tools and their limitations and a brief introduction to our approach.

Fig 1 summarizes the need for associative measures, major current approaches and briefly introduces the new approach suggested in this paper.

### Results on synthetic data

**Fig 2:**
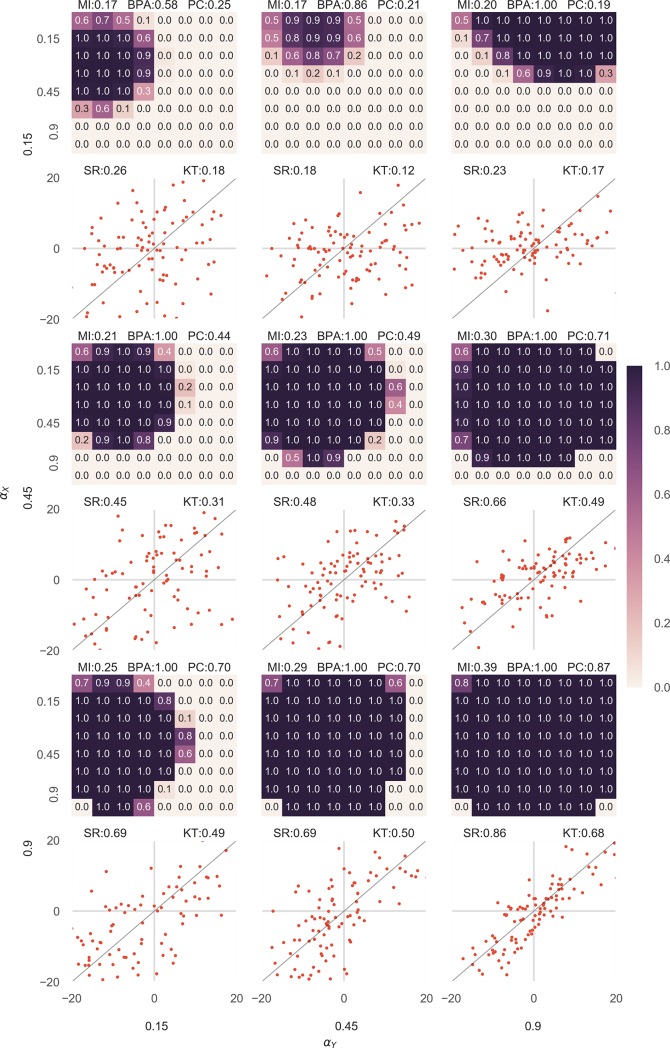
Results given for various combinations of *α*_*X*_ and *α*_*Y*_ in associated datasets were better than Pearson correlation coefficient in the majority of cases. 9 heatmaps are shown in the figure, each representing one combination of *α*_*X*_ and *α*_*Y*_. Each heatmap, calculates BPA for different combinations of $\alpha_{X}^{\prime }$ and $\alpha_{Y}^{\prime }.$ *I*_*X*_ was kept equal to *I*_*Y*_ for the dataset to be associated and *X* was calculated by the addition of *I*_*X*_ and *N*_*X*_ and similarly Y. The value of BPA obtained is encoded in color, the darker being higher. Further, below every heatmap the corresponding scatterplot for X and Y is shown. The straight black line shows the relationship function. On the top of every heatmap the normalized mutual information content (MI), the Bayesian Probability of Association (BPA, for the correct choice of $\alpha_{X}^{\prime }$ and $\alpha_{Y}^{\prime }$ which is equal to *α*_*X*_ and *α*_*Y*_ respectively) and the Pearson correlation value (PC) has been displayed. On the top of every scatter plot, the values of the Spearman Rank Correlation (SR) and the Kendall Tau Rank Correlation (KT) is also shown. It can be observed that when $\alpha_{X}^{\prime }$ and $\alpha_{Y}^{\prime }$ exceed *α*_*X*_ and *α*_*Y*_ respectively the results start to approach 0.

To test the approach, we generated 100 data points for two variables, *X* and *Y* using a random number generator (of the specifically chosen probability distribution) in the python programming language. In the first case which tested for associated data, *I*_*X*_, *N*_*X*_, *I*_*Y*_, *N*_*Y*_ were generated by a normally distributed random variable of mean 0 and *I*_*X*_ was kept equal to *I*_*Y*_ (thus defining the relationship function as the identity function). *X* was calculated by the addition of *I*_*X*_ and *N*_*X*_ and similarly Y. The variance of the three variables were varied in a way that all combinations of *α*_*X*_ and *α*_*Y*_ in the range 0.01 to 0.99 at a gap of 0.15 were tested for all possible combinations of $\alpha_{X}^{\prime }$ and $\alpha_{Y}^{\prime }$ in the same range. It was observed that as long as $\alpha_{X}^{\prime }$ and $\alpha_{Y}^{\prime }$ (estimated proportions of information in both the variables) were less than *α*_*X*_ and *α*_*Y*_ (actual proportions of information in both the variables), the association was strongly predicted while the probability of association quickly dropped almost to 0 once the $\alpha_{X}^{\prime }$ and $\alpha_{Y}^{\prime }$ exceeded *α*_*X*_ and *α*_*Y*_ (Fig 2, S2 File). This can be explained as when the variance of information is estimated less than the actual value, probability of values far from the mean get multiplied twice in *L*(*x*,*y*|*H*_2_) thus reducing it. Also, in this scenario as the variance of the noise variable is high, large values of noise do not have a very low probability and thus do not reduce *L*(*x*,*y*|*H*_1_). Both these effects are reversed when the variance of information is estimated to be higher than it actually is and the results quickly fall towards 0. This property can be used to study causal directions as generally most of the noise of the cause is reflected in the effect, hence the *α* for cause would likely be higher than the *α* for effect [20].

For the sake of clarity, Fig 2 shows the results for 9 out of the 64 datasets that were tested. The detailed results are shown in S2 File. Further, for all the 64 datasets, the correct Bayesian Probability of Association (BPA for $\alpha_{X}^{\prime }=\alpha_{X}$ and $\alpha_{Y}^{\prime }=\alpha_{Y}$), the normalized mutual information content, the Pearson Correlation Coefficient, Spearman Rank Correlation and Kendall Tau Rank Correlation Coefficient were also calculated (S2 File).

**Fig 3:**
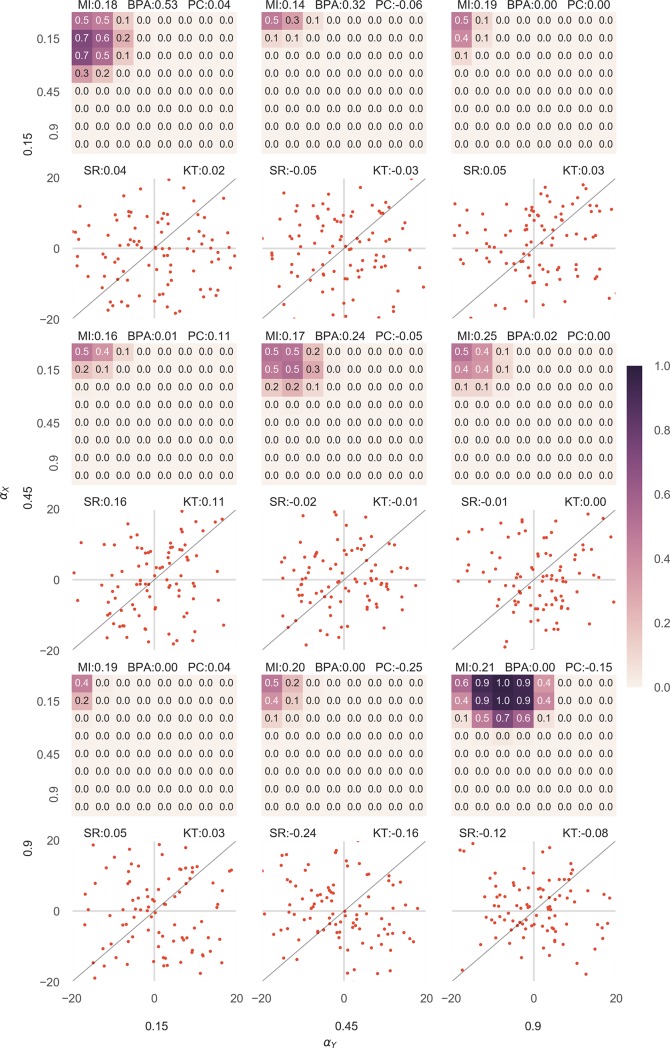
Results given for various combinations of *α*_*X*_ and *α*_*Y*_ in unassociated datasets outperformed Pearson correlation coefficient in the majority of cases. The figure is arranged in the exact same way as Fig 2. *I*_*X*_ and *I*_*Y*_ were calculated independently for the dataset to be unassociated and *X* was calculated by the addition of *I*_*X*_ and *N*_*X*_ and similarly Y. The value of BPA obtained is encoded in color, the darker being higher. As in Fig 2, below every heatmap the corresponding scatterplot for X and Y is shown. The straight black line shows the relationship function. On the top of every heatmap the normalized mutual information content (MI), the Bayesian Probability of Association (BPA, for the correct choice of $\alpha_{X}^{\prime }$ and $\alpha_{Y}^{\prime }$ which is equal to *α*_*X*_ and *α*_*Y*_ respectively) and the Pearson correlation value (PC) has been displayed. On the top of every scatter plot, the values of the Spearman Rank Correlation (SR) and the Kendall Tau Rank Correlation (KT) is also shown.

In the second case, we tested the results of the approach for unassociated datasets. All the four variables were generated in the same way, except in this case *I*_*X*_ and *I*_*Y*_ were generated independently thus leaving no associated component in the two variables. It was also observed that in the second case when the data was not associated, the approach had a tendency to suggest false associations if $\alpha_{X}^{\prime }$ and $\alpha_{Y}^{\prime }$ are kept very low but not otherwise (Fig 3, S2 File). This can be explained by the same argument due to which true associations were strongly suggested even when the variance of information was estimated to be lower than its actual value. But false associations were suggested much less than true associations (Fig 3).

Similar to Fig 2, Fig 3 shows the results for 9 out of 64 unassociated datasets. S2 File shows the detailed results and lists the values of other correlation coefficients along with the correct BPA (BPA for $\alpha_{X}^{\prime }=\alpha_{X}$ and $\alpha_{Y}^{\prime }=\alpha_{Y}$).

**Fig 4:**
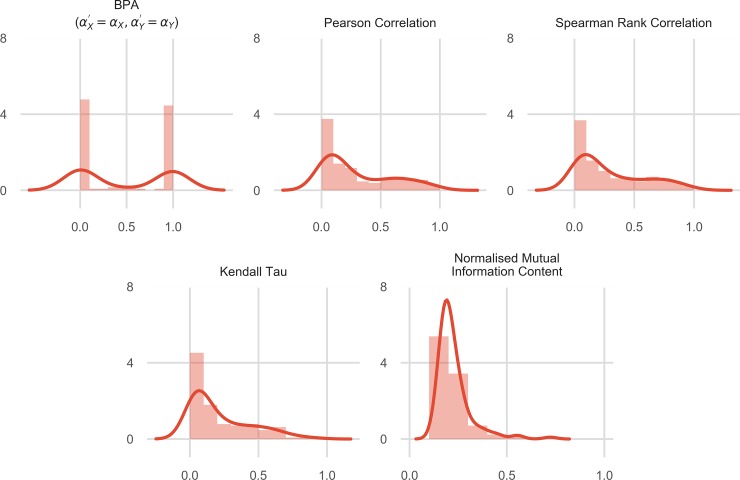
Distribution of various associational measures over the range of 0 to 1 for all the synthetic datasets. The distribution of results of all the five measures over all the synthetic datasets is shown. For every dataset, only the correct BPA was chosen. Further, absolute values were taken for Pearson Correlation Coefficient, Spearman Rank Correlation Coefficient and Kendall Tau Rank Correlation to bring all the measures on the same scale. Observe how a more negative value also suggests a stronger correlation.

The distribution of the various associational measures was tested for the 128 synthetic datasets (Fig 4). It was observed that while other measures had a more smoother distribution over 0 to 1, BPA (for $\alpha_{X}^{\prime }=\alpha_{X}$ and $\alpha_{Y}^{\prime }=\alpha_{Y}$) had a sharp peak around both 1 and 0. This suggested that BPA can be a better tool if our purpose is to observe the magnitude of correlation, but to classify two variables to be associated or not as it leaves a wider space (over 0 to 1) between the two classes and hence improving the confidence of our results. It was also observed that even though an equal number of datasets were associated and unassociated all the distributions were skewed towards 0, with the least skew in BPA (as even BPA fails for associated datasets with very low *α*_*X*_ and *α*_*Y*_).

### Results on real world data

The new approach was tested on 75 datasets from a cause-effect database [21] and 69 datasets were predicted to be associated. All 75 datasets had variables that had defined causal relationships (S3 File). It was also tested on 15 unassociated datasets and no false associations were found (S4 File). The datasets that were used for this study covered a wide range of disciplines. Some associated datasets included geographical variables like the relationship between altitude and temperature, and altitude and precipitation. Further, some associated datasets were taken from the abalone dataset that explored various biological associations. Also, physical associations were explored for instance the association between horsepower and mileage of a vehicle. Such a wide range of discipline shows that the approach is not limited to any branch and can be used for associational analysis in all branches of science.

**Fig 5:**
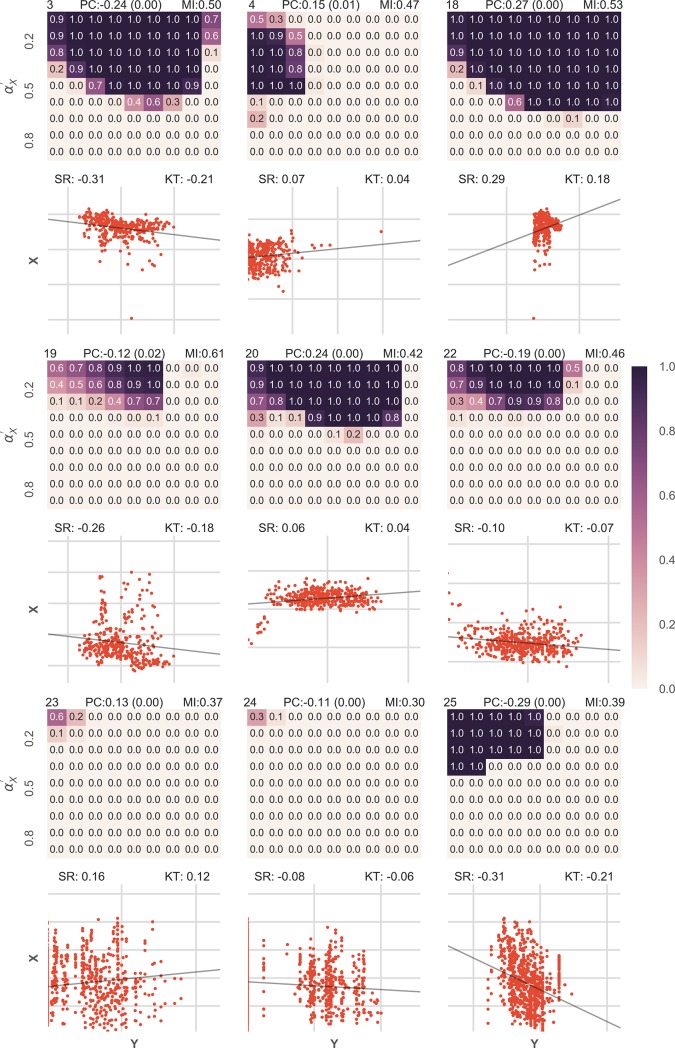
Association was detected in 19 out of 25 datasets with underlying association in the low Pearson correlation coefficient (<0.35) group: BPA for combinations of various $\alpha_{X}^{\prime }$ and $\alpha_{Y}^{\prime }$ are shown as a heatmap for 9 datasets. Below every heatmap, a scatterplot of the actual data is shown. Also, a black line shows the relationship function that was calculated by curve fitting approaches. On the top left of the heatmap, the unique index of every dataset is presented. On the top of the heatmap Pearson correlation coefficient (PC) along with the p-value in brackets and the normalized mutual information content (MI) for the dataset is given. On the top of the scatterplot, the Spearman rank correlation coefficient (SR) and the Kendall tau rank correlation coefficient (KT) are given.

**Fig 6:**
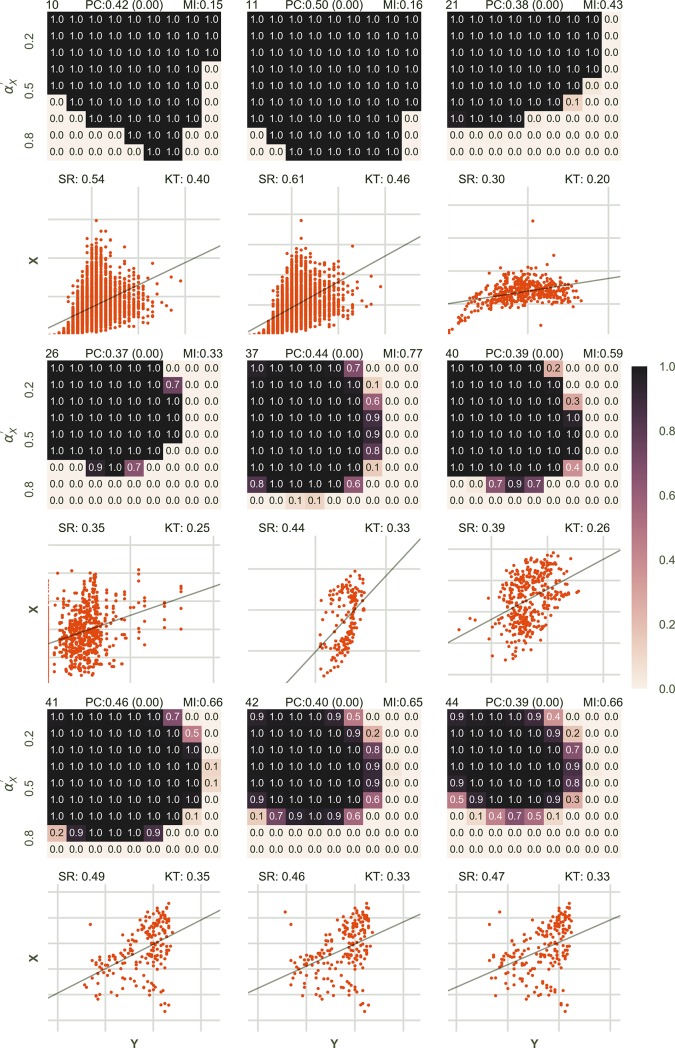
Association was detected in 25 out of 25 datasets with underlying association in the moderate Pearson correlation coefficient (0.35–0.63) group: The figure is arranged in exactly the same way as Figs 5 and 9 datasets have been shown. BPA for combinations of various $\alpha_{X}^{\prime }$ and $\alpha_{Y}^{\prime }$ are shown as a heatmap for 9 datasets. Below every heatmap, a scatterplot of the actual data is shown. Also, a black line shows the relationship function that was calculated by curve fitting approaches. On the top left of the heatmap, the unique index of every dataset is presented. On the top of the heatmap Pearson correlation coefficient (PC) along with the p-value in brackets and the normalized mutual information content (MI) for the dataset is given. On the top of the scatterplot, the Spearman rank correlation coefficient (SR) and the Kendall tau rank correlation coefficient (KT) are given.

**Fig 7:**
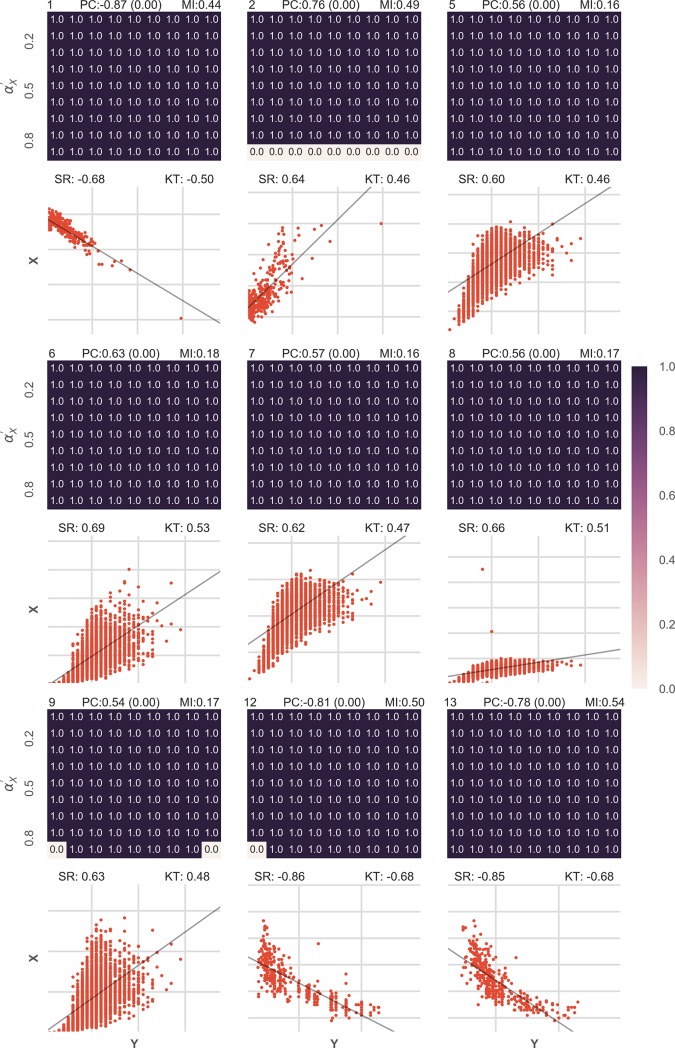
Association was detected in 25 out of 25 datasets with underlying association in the high Pearson correlation coefficient (>0.63) group: The figure is arranged in exactly the same way as Fig 5. BPA for combinations of various $\alpha_{X}^{\prime }$ and $\alpha_{Y}^{\prime }$ are shown as a heatmap for 9 datasets. Below every heatmap, a scatterplot of the actual data is shown. Also, a black line shows the relationship function that was calculated by curve fitting approaches. On the top left of the heatmap, the unique index of every dataset is presented. On the top of the heatmap Pearson correlation coefficient (PC) along with the p-value in brackets and the normalized mutual information content (MI) for the dataset is given. On the top of the scatterplot, the Spearman rank correlation coefficient (SR) and the Kendall tau rank correlation coefficient (KT) are given.

**Fig 8:**
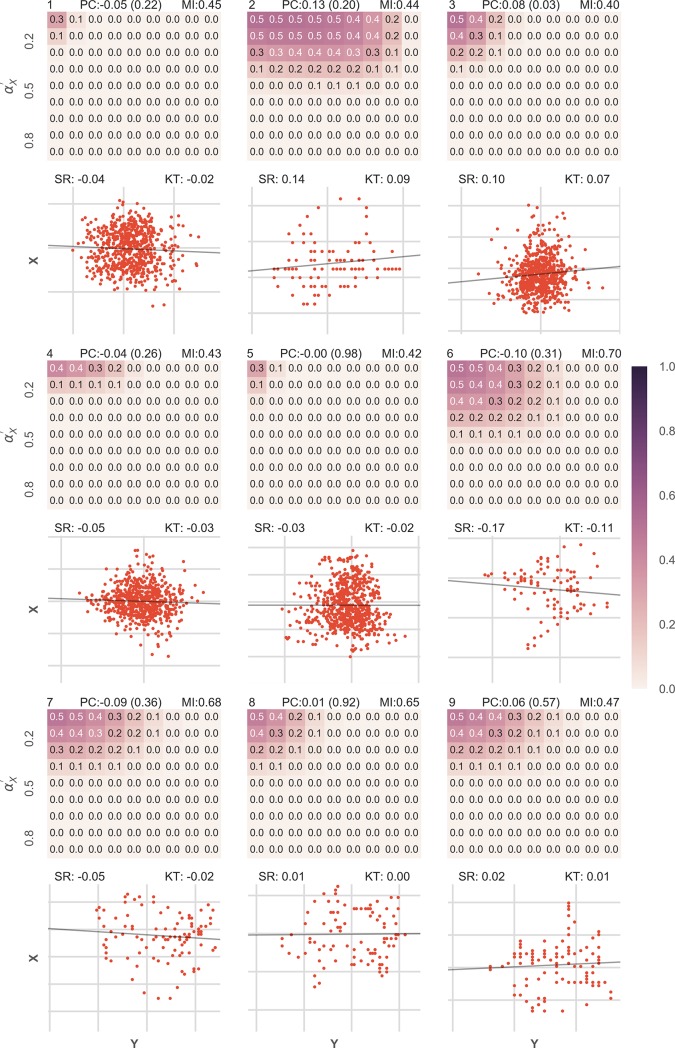
Results for 15 datasets with no underlying association: Data from different EEG recordings was taken and the channel (electrode position) was picked at random. As two different recordings must not have any underlying association, BPA was checked for different combinations of $\alpha_{X}^{\prime }$ and $\alpha_{Y}^{\prime }$ and it was found that the results were pretty low. The figure is arranged in exactly the same way as Figs 5 and 9 datasets have been shown. Below every heatmap, a scatterplot of the actual data is shown. Also, a black line shows the relationship function that was calculated by curve fitting approaches. On the top left of the heatmap, the unique index of every dataset is presented. On the top of the heatmap Pearson correlation coefficient (PC) along with the p-value in brackets and the normalized mutual information content (MI) for the dataset is given. On the top of the scatterplot, the Spearman rank correlation coefficient (SR) and the Kendall tau rank correlation coefficient (KT) are given.

The new approach was applied by estimating that all the four variables, *I*_*X*_, *N*_*X*_, *I*_*Y*_, *N*_*Y*_ have a normal distribution. The mean of the variables was subtracted from the respective variable so that the mean can be estimated to be 0 for all the four variables. Further, variance of both the variables were calculated and all combinations of $\alpha_{X}^{\prime }$ and $\alpha_{Y}^{\prime }$ in the range 0.1 to 0.9 at a gap of 0.1 were tested. The underlying relationship was calculated by finding the linear regression function in the data. The fitted regression line has been shown in the figures (Figs 2–3, Figs 5–8). S3 and S4 Files should be referred for the slope and intercept values for all the datasets. Further, to predict the causal direction in the dataset we calculated two parameters,

$$S_{X}=\sum_{\alpha_{Y}^{\prime }=0.1}^{0.9}\sum_{\alpha_{X}^{\prime }=0.1}^{0.9}\alpha_{X}^{\prime }*P(H_{1})$$

$$S_{Y}=\sum_{\alpha_{Y}^{\prime }=0.1}^{\alpha_{Y}^{\prime }=0.9}\sum_{\alpha_{X}^{\prime }=0.1}^{\alpha_{X}^{\prime }=0.9}\alpha_{Y}^{\prime }*P(H_{1})$$

As observed, when $\alpha_{X}^{\prime }$ or $\alpha_{Y}^{\prime }$ exceeds *α*_*X*_ and *α*_*Y*_ respectively, BPA tends to drop towards 0. Hence, the suggested parameters were used to identify which of the two variables had a higher information content, in other words which of the two was higher, *α*_*X*_ or *α*_*Y*_. As explained, this information can help distinguish the cause from the effect as generally the noise content in the effect is higher than that in the cause [20].

The results on 27 selected datasets divided into three equal sized groups are presented (Figs 5, 6 and 7). Interested readers can refer to a detailed report of all the 75 datasets which can also be divided into three equal sized groups based on the absolute values of Pearson Correlation Coefficient (S3 File). BPA was assumed to be suggesting association if it suggested a probability of 1 for any combination of $\alpha_{X}^{\prime }$ or $\alpha_{Y}^{\prime }$.

The first group had datasets with the absolute Pearson Correlation coefficient values of less than 0.35 (Fig 5) which is the low correlation group. Out of all the datasets that had a low correlation, it was observed that for 19 out of 25 datasets, BPA could identify the underlying association. The cases where BPA could not identify the association had Pearson Correlation coefficient values less than 0.18.

The second group had datasets with the Pearson correlation coefficient values from 0.35 to 0.63 (Fig 6) which was the moderate correlation group. Please note that we have taken the value of 0.63 for the sake equally dividing the three groups. Association was identified in all the 25 datasets that had a moderate Pearson Correlation value. It was also observed that in some cases where the scatterplot showed a tight relationship, Pearson Correlation Coefficient was low just because of the non-linearity of the association. BPA could identify association in all such scenarios.

The third group had datasets with high Pearson Correlation values higher than 0.63 (Fig 7). This group is the high correlation group. It was observed that association was identified for all the 25 datasets and for almost all combinations of $\alpha_{X}^{\prime }$ or $\alpha_{Y}^{\prime }$. This suggests that for stronger associations, BPA will identify the association even with much more approximate estimations.

Overall, it was observed that association were predicted for 69 out of 75 datasets, whereas the absolute value of Pearson correlation coefficient was more than 0.5 for only 35 datasets (Fig 4). Further, Normalized Mutual Information Content was usually lower than Pearson Correlation Coefficient. Spearman Rank Correlation Coefficient and Kendall Tau Rank Correlation performed similar to the Pearson Correlation Coefficient. It was also observed that for datasets which had high Pearson correlation coefficient (more than 0.5), most of the combinations of $\alpha_{X}^{\prime }$ or $\alpha_{Y}^{\prime }$ in the tested range predicted the probability of association to be close to 1. Further, for 31 of the datasets, the causal direction could not be predicted because of high association between the variables because of which highest tested values of $\alpha_{X}^{\prime }$ or $\alpha_{Y}^{\prime }$ probably remained less than *α*_*X*_ and *α*_*Y*_. This led to *BPA* ≈ 1 for all combinations of $\alpha_{X}^{\prime }$ or $\alpha_{Y}^{\prime }$. Hence, for these cases *S*_*X*_ ≈ *S*_*Y*_. For such cases, higher resolution of the combinations of $\alpha_{X}^{\prime }$ or $\alpha_{Y}^{\prime }$ are expected to reveal the correct causal direction. In the remaining cases 35 times the causal direction was predicted correctly, and 9 times incorrectly.

To show that the suggested approach identifies cases of independence correctly, we took 15 examples from an EEG dataset [22–24]. We took combinations of data from different recordings and calculated the results in exactly the same way as for the cause-effect dataset. The results were quite low in all the fifteen cases for all the combinations of $\alpha_{X}^{\prime }$ or $\alpha_{Y}^{\prime }$ (Fig 8, S4 File) and no false associations were detected.

### Results with non-linear relationship

**Fig 9:**
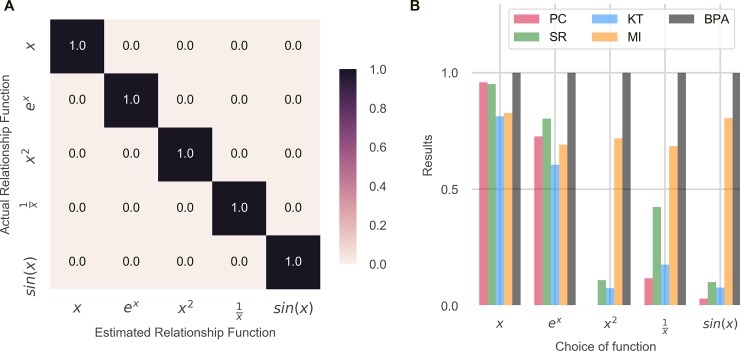
**The new approach could identify associations in cases of non-linearity**: A), Five different relationship functions were taken and the results of the new approach were tested for various combinations of actual and estimated relationship functions. It was observed that the new approach could identify associations when the correct relationship function was estimated. B), Results were compared with the Pearson Correlation Coefficient (PC), the Spearman Rank Correlation Coefficient (SR), the Kendall Tau Rank Correlation Coefficient (KT) and the normalized mutual information content (MI). It was observed that while the three correlation coefficients could not identify associations in all the cases, Bayesian Probability of Association (BPA) could. Normalized Mutual Information Content was lower than the suggested approach but as seen it was higher than Pearson Correlation Coefficient.

We tested BPA for various underlying functions and checked if the new approach could identify non-linear associations any better than other measures (Fig 9). It was observed that BPA performed better than all the Pearson Correlation Coefficient, the Normalized Mutual Information Content, Spearman Rank Correlation Coefficient and Kendall Tau Rank Correlation Coefficient in identifying non-linear associations. We took five relationship functions and checked if the new approach could identify associations with different estimations of the relationship function. It was observed in all the cases the approach could identify association when the correct relationship function was estimated (Fig 9A). The variance of noise was kept as half the variance of information so that other measures could work well. It was also observed that the Normalized Mutual Information Content was higher than the three correlation coefficients and was more robust to different functions but was still lesser than BPA (Fig 9B).

However, it should be noted that although the correlation coefficients do not work very good in identifying non-linear relationships, the same way that we estimate the relationship function for BPA, we can estimate a relationship function and transform the data with the estimated relationship function before calculating the coefficients.

Please note that to apply the approach to different relationship functions, one needs to change the integral limits in the likelihood calculation according to the domain and the range of the relationship function.

## Conclusion and discussion

The new approach calculates the probability of two given datasets being associated by utilizing the underlying probability distributions of information and noise in the variables. Further, to take into account all the data points, it uses Bayesian ideas to update the beliefs on association in the dataset. Results show that the suggested approach makes good predictions in the absence of any knowledge about the underlying system and further knowledge of the underlying system will only further improve the results. The suggested approach was found to be performing better than both Pearson Correlation Coefficient and Normalized Mutual Information Content. Also, the suggested approach provides a method where externally known information about the system can be incorporated in the calculation of the probability of association for better results.

External information about the system can be incorporated at basically three points in the given system. Firstly, in the underlying distributions. As explained, probability distributions of the underlying variables provide valuable information in the prediction of association. By leaving the option of choosing an underlying distribution, this approach also overcomes the limitation of the assumption of normality faced by Pearson correlation coefficient. Secondly, external information can also be incorporated into the underlying relationship function and hence BPA can identify associations that are non-linear in nature. However, it must be noted that the same can also be done for Pearson Correlation Coefficient by transforming the variables based on the known function. Lastly, in the initial probabilities assigned to *H*_1_ and *H*_2_. If there is a reason to believe one over the other, the prior belief can be incorporated in the system by changing the initial probabilities of *H*_1_ and *H*_2_.

While associations have been studied for a long time, they have never been studied by considering noise and information separately in a variable. Our approach, by differentiating between noise and information leads to a better estimation of association as the sensitivity to noise in the coefficient can be controlled by setting the distribution of noise. We expect this approach to be developed on the same lines as other correlation coefficients. Other correlation approaches are used in canonical correlation analysis [25], cross-correlation analysis and time lagged correlation analysis [26]. These approaches apply correlation coefficients in different ways to identify associations in a system. Our approach is valid for all of the given methods. Further, we expect this approach to become the primary tool in the analysis of variables with a large amount of noise and where external domain specific inputs can be given to improve results.

To understand the properties of BPA, we analyzed all combinations of $\alpha_{X}^{\prime }$ and $\alpha_{Y}^{\prime }$ but to just study associations, faster algorithmic approaches like binary search [27] and randomized searches [28] can be used to find the optimal values of *α*_*X*_ and *α*_*Y*_. However, there cannot be a fixed approach to choose values of *α*_*X*_ and *α*_*X*_ that reveal true association among variables. The noise to information ratio varies in variables in different branches of science and hence the results of BPA depend on domain specific knowledge. We expect such methods to be used when the suggested approach gains popularity. Further, such methods will support higher resolutions of the values $\alpha_{X}^{\prime }$ and $\alpha_{Y}^{\prime }$ and in turn also improve the prediction of causal directions.

To predict causal directions more efficiently some information in this approach remains unexploited. As $\alpha_{X}^{\prime }$ and $\alpha_{Y}^{\prime }$ exceed *α*_*X*_ and *α*_*Y*_, we observe that BPA starts to tend to 0, a property which can be exploited to get an idea of the value of *α*_*X*_ and *α*_*Y*_. While in many cases, the final value of BPA might be almost 1 for all tested values of *α*_*X*_ and *α*_*Y*_, hence failing to predict causal directions. In such scenarios, the rate of increase of BPA with respect to the number of data points consumed can help us predict which of the two, *α*_*X*_ or *α*_*Y*_ are higher.

However, there are some limitations of BPA that we have identified. Firstly, it is more computationally intensive than the Pearson Correlation Coefficient. With the advances in computing technologies, we strongly believe that this will hardly be a hindrance in the development of BPA. Secondly, the approach inherently requires some brute-force searching for an appropriate estimation of the underlying distributions and the relationship function. But we have also shown that the approach gives accurate results even in cases where approximate estimations are made. Lastly, just like the Pearson Correlation Coefficient, BPA is not devoid of false positives or false negatives and some false results are inevitable.

We expect BPA to have applications in all branches of science. Further, we expect that BPA will help advance the study of associations by extending them beyond correlation analysis. It is worth noting that even though association and correlation are related concepts, they do not imply each other. Two variables which are associated may not have a high correlation between them due to a large amount of noise. Hence, we expect associational analysis to have another layer of analysis beyond correlations. It should also be noted that while correlation by definition is a continuous variable, association is binary. Two variables either will be associated or not. We believe BPA is a good measure of association as it classifies results into 0 or 1 for most cases (Fig 4). We expect BPA to be developed further to quantify the amount of noise in the variables and to study causal directions in all cases. Further, with the advancement in computing power we expect BPA to become fast and be widely used.

## Supporting information

S1 File Code for calculating Bayesian Probability of association. The file shows the code for calculating Bayesian Probability of Association written in python 3.5.
(PDF)

S2 File Detailed results for synthetic datasets. The detailed results for synthetic datasets are presented as heatmaps and also as a table.
(PDF)

S3 File Detailed results for associated real world datasets. The detailed results for the real world associated datasets are presented including a heatmap of BPA results, a scatter plot and the numerical values of all the measures compared in the manuscript.
(PDF)

S4 File Detailed results for unassociated real world datasets. The detailed results for the real world unassociated datasets are presented in the same manner as S3 File.
(PDF)
