# Supplementary material for: A Bayesian measure of association that utilizes the underlying distributions of noise and information
Source: PLoS One. 2018 Aug 17;13(8):e0201185. doi: 10.1371/journal.pone.0201185 (PMC6097650; doi:10.1371/journal.pone.0201185)
Supplement: S1 File — The file shows the code for calculating Bayesian Probability of Association written in python 3.5. (PDF) [file pone.0201185.s001.pdf]

```
from scipy.integrate import quad
from scipy.stats import norm
import numpy as np
import scipy.stats as stats

def likelihood_h1(x, data1, data2, f_X, f_e1, f_e2, param_X, param_e1, param_e2, f):
	return f_X(x, param_X)*f_e1(data1 - x, param_e1)*f_e2(data2 - f(x), param_e2)

def likelihood_h2(x, data1, f_X, f_e1,param_X, param_e1):
	return f_X(x, param_X)*f_e1(data1 - x,param_e1)

def calculatePosterior(prior_h1, prior_h2, data, f_X, f_Y, f_e1, f_e2, param_X, param_Y,
param_e1, param_e2, f1, f2):
	X = data[0]
	Y = data[1]

	likelihood_givenh1_X = quad(likelihood_h1, -np.inf, np.inf, args = (X,Y, f_X, f_e1,
f_e2, param_X, param_e1, param_e2,f1))[0]
	likelihood_givenh1_Y = quad(likelihood_h1, -np.inf, np.inf, args = (Y,X, f_Y, f_e2,
f_e1, param_Y, param_e2, param_e1, f2))[0]
	likelihood_givenh1 = 0.5*likelihood_givenh1_X + 0.5*likelihood_givenh1_Y
	likelihood_givenh2 = quad(likelihood_h2, -np.inf, np.inf, args = (X,f_X, f_e1,
param_X, param_e1))[0]*quad(likelihood_h2, -np.inf, np.inf, args = (Y, f_Y, f_e2, param_Y,
param_e2))[0]

	posterior_h1 = (prior_h1*likelihood_givenh1)/ ((prior_h1*likelihood_givenh1) +
(prior_h2*likelihood_givenh2))
	posterior_h2 = (prior_h2*likelihood_givenh2)/ ((prior_h1*likelihood_givenh1) +
(prior_h2*likelihood_givenh2))
	return [posterior_h1, posterior_h2]

# X is a list containing the data for the first variable
# Y is a list containing the data for the second variable
# f_X is the probability density function for X. param_X are the parameters of that function.
# f_Y is the probability density function for X. param_Y are the parameters of that function.
# f_e1 is the probability density function for X. param_e1 are the parameters of that
function.
# f_e2 is the probability density function for X. param_e2 are the parameters of that
function.
#f1 is the relationship function Y = f1(X)
#f2 is the inverse of f1. X = f2(Y)
#use this function as the entrypoint to the code.

def calculateProbability(X,Y, f_X, f_Y, f_e1, f_e2, param_X, param_Y, param_e1, param_e2,
f1, f2):
	size_X = len(X)
	size_Y = len(Y)
```

```
	size = min(size_X, size_Y)
	prob_h1 = 0.5
	prob_h2 = 0.5
	list_probh1 = [0.5]
	for i in range(size):
		prob_h1, prob_h2 = calculatePosterior(prob_h1, prob_h2, [X[i], Y[i]], f_X, f_Y, 
f_e1, f_e2, param_X, param_Y, param_e1, param_e2,f1,f2)
		print(prob_h1, prob_h2)
		list_probh1.append(prob_h1)
		if((prob_h1 == 0)or(prob_h1 == 1)):
			break
		if((prob_h2 == 0)or(prob_h2 == 1)):
			break
	return prob_h1, list_probh1
```
