# Supplementary material for: A Bayesian measure of association that utilizes the underlying distributions of noise and information
Source: PLoS One. 2018 Aug 17;13(8):e0201185. doi: 10.1371/journal.pone.0201185 (PMC6097650; doi:10.1371/journal.pone.0201185)
Supplement: S2 File — The detailed results for synthetic datasets are presented as heatmaps and also as a table. (PDF) [file pone.0201185.s002.pdf]

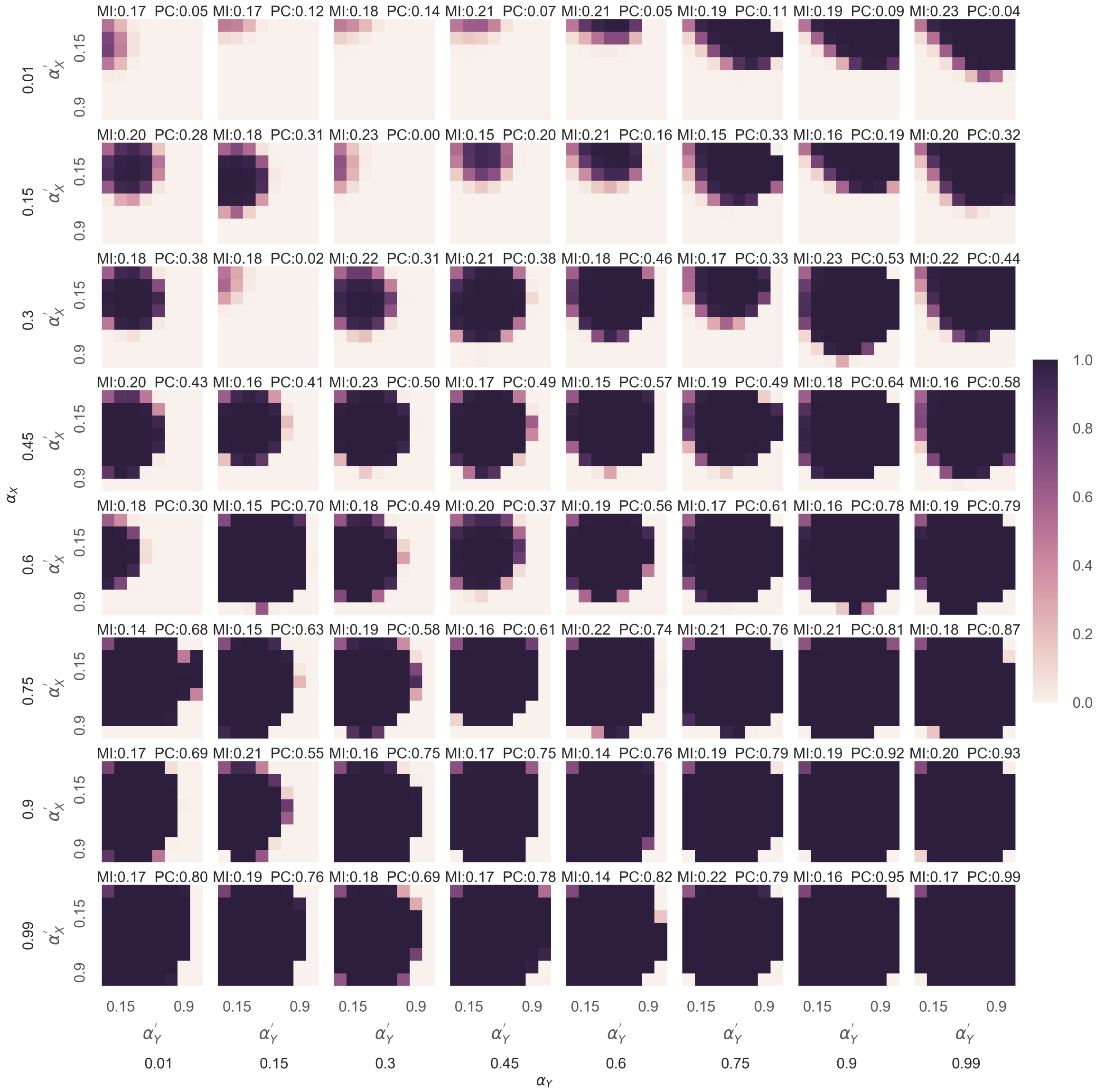

Fig S1: Results given for various combinations of α_X and α_Y in associated datasets were better than Pearson Correlation Coefficient in the majority of cases. 964 heatmaps are shown in the figure, each representing one combination of α_X and α_Y. Each heatmap, calculates BPA for different combinations of α'_X and α'_Y. I_X was kept equal to I_Y for the dataset to be associated and X was calculated by the addition of I_X and N_X and similarly Y. The value of BPA obtained is encoded in color, the darker being higher. On the top right of every heatmap the Pearson correlation value (PC) and the normalized mutual information content (MI) has been displayed. It can be observed that when α'_X and α'_Y exceed α_X and α_Y respectively the results start to approach 0.

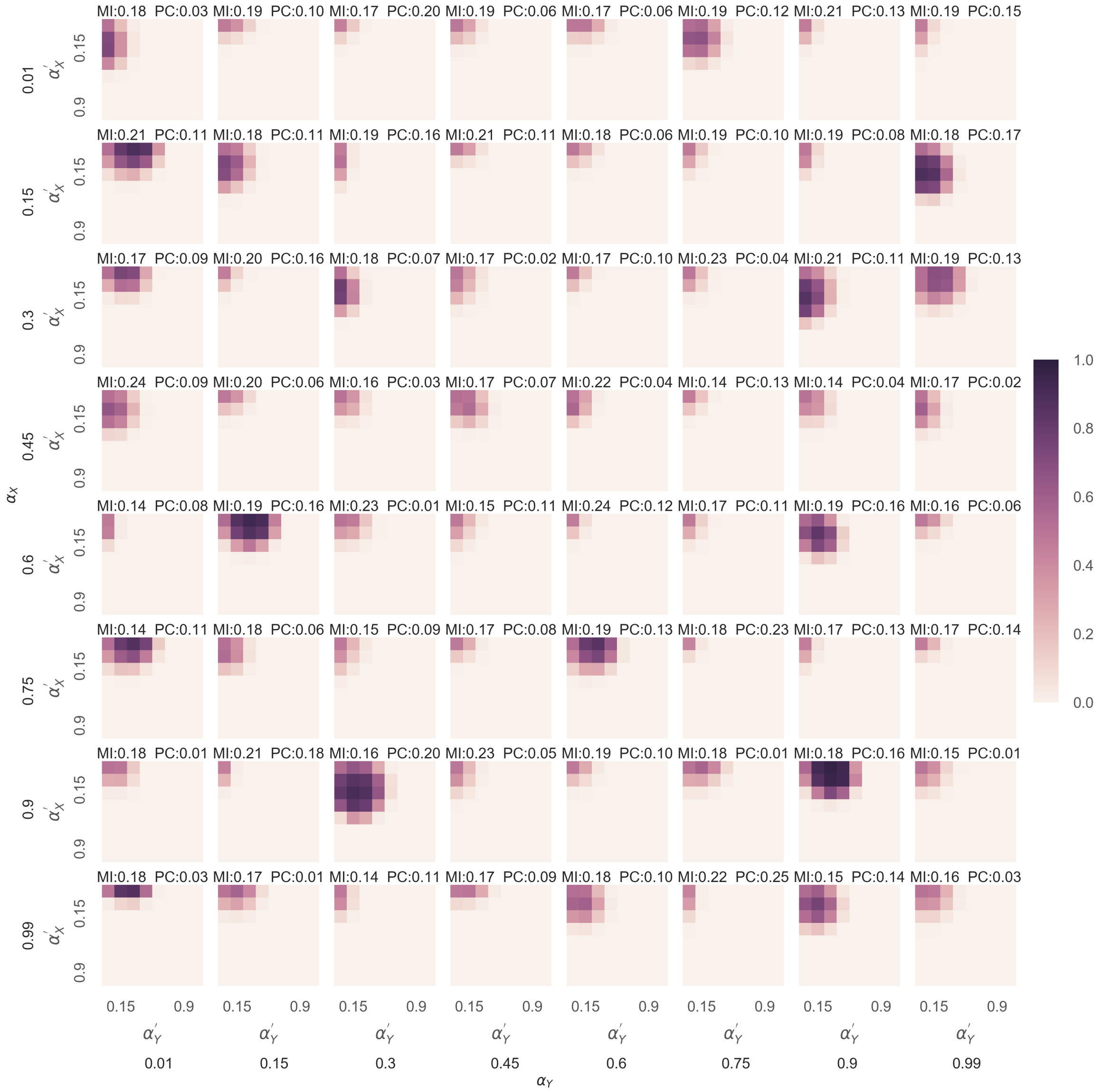

Fig S2: Results given for various combinations of α_X and α_Y in unassociated datasets were low for most cases. 64 heatmaps are shown in the figure, each representing one combination of α_X and α_Y. Each heatmap, calculates BPA for different combinations of α'_X and α'_Y in the same way as Fig S1. To keep the datasets unassociated, I_X and I_Y were generated independently. The value of BPA obtained is encoded in color, the darker being higher. On the top of every heatmap the Pearson correlation value (PC) and the mutual information content (MI) has been displayed. It can be observed that for very low values of α'_X and α'_Y the approach starts to give false associations.

Results on Associated Synthetic Datasets

Ratio X	Ratio Y	BPA	MI	PC	SR	KT
0.01	0.01	0.51	0.18	-0.01	-0.04	-0.03
0.01	0.15	0.45	0.13	0.08	0.10	0.06
0.01	0.30	0.21	0.16	-0.13	-0.15	-0.10
0.01	0.45	0.44	0.22	-0.14	-0.16	-0.11
0.01	0.60	0.99	0.17	0.02	-0.09	-0.05
0.01	0.75	1.00	0.19	0.03	0.02	0.01
0.01	0.90	1.00	0.19	0.09	0.12	0.09
0.01	0.99	1.00	0.17	0.08	0.08	0.06
0.15	0.01	0.87	0.18	0.22	0.18	0.12
0.15	0.15	0.98	0.22	0.18	0.17	0.12
0.15	0.30	0.02	0.19	0.12	0.04	0.03
0.15	0.45	0.91	0.19	0.35	0.37	0.26
0.15	0.60	0.99	0.18	0.22	0.23	0.15
0.15	0.75	1.00	0.22	0.21	0.27	0.19
0.15	0.90	1.00	0.19	0.36	0.38	0.26
0.15	0.99	1.00	0.22	0.35	0.28	0.19
0.30	0.01	0.99	0.19	0.21	0.21	0.15
0.30	0.15	0.09	0.20	0.36	0.36	0.24
0.30	0.30	0.99	0.19	0.25	0.28	0.19
0.30	0.45	1.00	0.17	0.28	0.27	0.19
0.30	0.60	1.00	0.22	0.45	0.41	0.27
0.30	0.75	1.00	0.20	0.40	0.37	0.26
0.30	0.90	1.00	0.20	0.49	0.48	0.33
0.30	0.99	1.00	0.29	0.59	0.54	0.39
0.45	0.01	1.00	0.22	0.37	0.36	0.25
0.45	0.15	1.00	0.17	0.28	0.30	0.20
0.45	0.30	1.00	0.23	0.42	0.40	0.27
0.45	0.45	1.00	0.25	0.45	0.41	0.28
0.45	0.60	1.00	0.26	0.55	0.54	0.37
0.45	0.75	1.00	0.24	0.58	0.48	0.34
0.45	0.90	1.00	0.28	0.60	0.62	0.44
0.45	0.99	1.00	0.25	0.67	0.68	0.48
0.60	0.01	1.00	0.16	0.36	0.36	0.24
0.60	0.15	1.00	0.26	0.63	0.62	0.42
0.60	0.30	1.00	0.24	0.52	0.46	0.32
0.60	0.45	1.00	0.27	0.54	0.58	0.41
0.60	0.60	1.00	0.27	0.62	0.54	0.38
0.60	0.75	1.00	0.34	0.70	0.69	0.51
0.60	0.90	1.00	0.24	0.68	0.67	0.49
0.60	0.99	1.00	0.29	0.77	0.75	0.55
0.75	0.01	1.00	0.24	0.53	0.45	0.31
0.75	0.15	1.00	0.30	0.62	0.65	0.47
0.75	0.30	1.00	0.25	0.61	0.59	0.41

0.75	0.45	1.00	0.25	0.62	0.63	0.44
0.75	0.60	1.00	0.33	0.78	0.76	0.57
0.75	0.75	1.00	0.31	0.74	0.78	0.58
0.75	0.90	1.00	0.39	0.83	0.83	0.65
0.75	0.99	1.00	0.45	0.89	0.87	0.70
0.90	0.01	1.00	0.25	0.55	0.54	0.38
0.90	0.15	1.00	0.25	0.56	0.49	0.35
0.90	0.30	1.00	0.27	0.70	0.68	0.48
0.90	0.45	1.00	0.26	0.66	0.70	0.50
0.90	0.60	1.00	0.30	0.81	0.81	0.63
0.90	0.75	1.00	0.37	0.84	0.79	0.61
0.90	0.90	1.00	0.46	0.90	0.90	0.73
0.90	0.99	1.00	0.56	0.96	0.94	0.80
0.99	0.01	1.00	0.27	0.71	0.71	0.53
0.99	0.15	1.00	0.26	0.74	0.71	0.53
0.99	0.30	1.00	0.33	0.80	0.80	0.60
0.99	0.45	1.00	0.37	0.81	0.78	0.60
0.99	0.60	1.00	0.38	0.85	0.84	0.65
0.99	0.75	1.00	0.42	0.88	0.87	0.69
0.99	0.90	1.00	0.55	0.96	0.94	0.80
0.99	0.99	1.00	0.72	0.99	0.99	0.92

BPA: Bayesian Probability of Association
MI: Normalized Mutual Information Content
PC: Pearson Correlation Coefficient
SR: Spearman Rank Correlation Coefficient
KT: Kendall Tau Rank Correlation Coefficient

Please note that BPA has been reported it has been reported for the correct alpha values

Results on Unassociated Synthetic Datasets

Ratio X	Ratio Y	BPA	MI	PC	SR	KT
0.01	0.01	0.51	0.17	0	-0.02	-0.02
0.01	0.15	0.38	0.19	0	-0.03	-0.02
0.01	0.3	0.01	0.17	-0.1	-0.1	-0.06
0.01	0.45	0	0.19	0.07	0.06	0.04
0.01	0.6	0	0.18	-0.02	0	-0.01
0.01	0.75	0	0.14	0.04	0.02	0.02
0.01	0.9	0	0.21	-0.04	0.01	0
0.01	0.99	0	0.16	0.03	0	0
0.15	0.01	0.35	0.23	-0.06	-0.04	-0.03
0.15	0.15	0.62	0.17	-0.02	-0.08	-0.05
0.15	0.3	0	0.17	0.07	0.04	0.03
0.15	0.45	0	0.14	0	-0.01	-0.01
0.15	0.6	0	0.22	-0.16	-0.19	-0.13
0.15	0.75	0	0.17	-0.03	-0.03	-0.02
0.15	0.9	0	0.17	-0.05	-0.06	-0.03
0.15	0.99	0	0.19	0.01	0.04	0.03
0.3	0.01	0.02	0.2	0.1	0.09	0.06
0.3	0.15	0	0.24	-0.06	-0.09	-0.06
0.3	0.3	0.02	0.21	0.02	-0.02	-0.01
0.3	0.45	0	0.21	-0.1	-0.05	-0.03
0.3	0.6	0	0.16	0.02	0.03	0.01
0.3	0.75	0	0.16	0.09	0.15	0.1
0.3	0.9	0	0.21	-0.12	-0.07	-0.05
0.3	0.99	0	0.19	0.27	0.29	0.19
0.45	0.01	0.12	0.2	0.16	0.17	0.11
0.45	0.15	0	0.17	0.1	0.09	0.06
0.45	0.3	0	0.19	0.16	0.17	0.11
0.45	0.45	0	0.17	0.06	0.1	0.07
0.45	0.6	0	0.2	-0.1	-0.12	-0.08
0.45	0.75	0	0.19	0	0.02	0.01
0.45	0.9	0	0.17	-0.08	-0.1	-0.07
0.45	0.99	0	0.19	0.03	0.04	0.03
0.6	0.01	0	0.18	0.11	0.11	0.08
0.6	0.15	0	0.13	0.08	0.09	0.06
0.6	0.3	0	0.23	0.12	0.13	0.09
0.6	0.45	0	0.18	0.08	0.08	0.05
0.6	0.6	0	0.23	0.21	0.23	0.14
0.6	0.75	0	0.2	-0.09	-0.13	-0.08
0.6	0.9	0	0.2	0.29	0.32	0.22
0.6	0.99	0	0.18	-0.25	-0.28	-0.19
0.75	0.01	0	0.22	-0.06	-0.06	-0.04
0.75	0.15	0	0.19	-0.18	-0.19	-0.12
0.75	0.3	0	0.2	-0.15	-0.21	-0.14

0.75	0.45	0	0.17	-0.1	-0.1	-0.07
0.75	0.6	0	0.17	0.03	-0.02	-0.01
0.75	0.75	0	0.16	0	0.01	0.01
0.75	0.9	0	0.23	-0.01	0.02	0.01
0.75	0.99	0	0.17	-0.21	-0.21	-0.14
0.9	0.01	0	0.18	0.03	0.07	0.05
0.9	0.15	0	0.2	-0.15	-0.15	-0.1
0.9	0.3	0	0.22	-0.21	-0.22	-0.16
0.9	0.45	0	0.17	0.04	0.05	0.03
0.9	0.6	0	0.19	0.04	0	0.01
0.9	0.75	0	0.19	-0.09	-0.02	-0.01
0.9	0.9	0	0.18	-0.03	-0.06	-0.04
0.9	0.99	0	0.2	0.04	0.05	0.04
0.99	0.01	0	0.17	-0.22	-0.19	-0.13
0.99	0.15	0	0.19	-0.15	-0.13	-0.09
0.99	0.3	0	0.14	0.18	0.2	0.14
0.99	0.45	0	0.2	-0.06	-0.09	-0.06
0.99	0.6	0	0.2	0.06	0.05	0.03
0.99	0.75	0	0.15	-0.2	-0.16	-0.11
0.99	0.9	0	0.21	0.1	0.11	0.07
0.99	0.99	0	0.17	0.05	0.06	0.03

BPA: Bayesian Probability of Association
MI: Normalized Mutual Information Content
PC: Pearson Correlation Coefficient
SR: Spearman Rank Correlation Coefficient
KT: Kendall Tau Rank Correlation Coefficient

Please note that BPA has been reported it has been reported for the correct alpha values
