# Supplementary material for: A Bayesian measure of association that utilizes the underlying distributions of noise and information
Source: PLoS One. 2018 Aug 17;13(8):e0201185. doi: 10.1371/journal.pone.0201185 (PMC6097650; doi:10.1371/journal.pone.0201185)
Supplement: S3 File — The detailed results for the real world associated datasets are presented including a heatmap of BPA results, a scatter plot and the numerical values of all the measures compared in the manuscript. (PDF) [file pone.0201185.s003.pdf]

S3 File - Detailed Results on Associated datasets

All the datasets were taken from http://archive.ics.uci.edu/ml/index.php. The datasets were already known to have a causal relationship between them. We have calculated Pearson Correlation (PC), Spearman Rank Correlation (SR), Kendall Tau Rank Correlation (KT), Normalized Mutual Information Content (MI) and the Bayesian Probability Of Association (BPA) for all the datasets. Further, BPA has also been used to predict causal directions in the dataset and it has been reported if the causal direction has been predicted correctly or not.

1 Dataset-1

x: altitude
y: temperature (average over 1961-1990)
ground truth:
x --> y

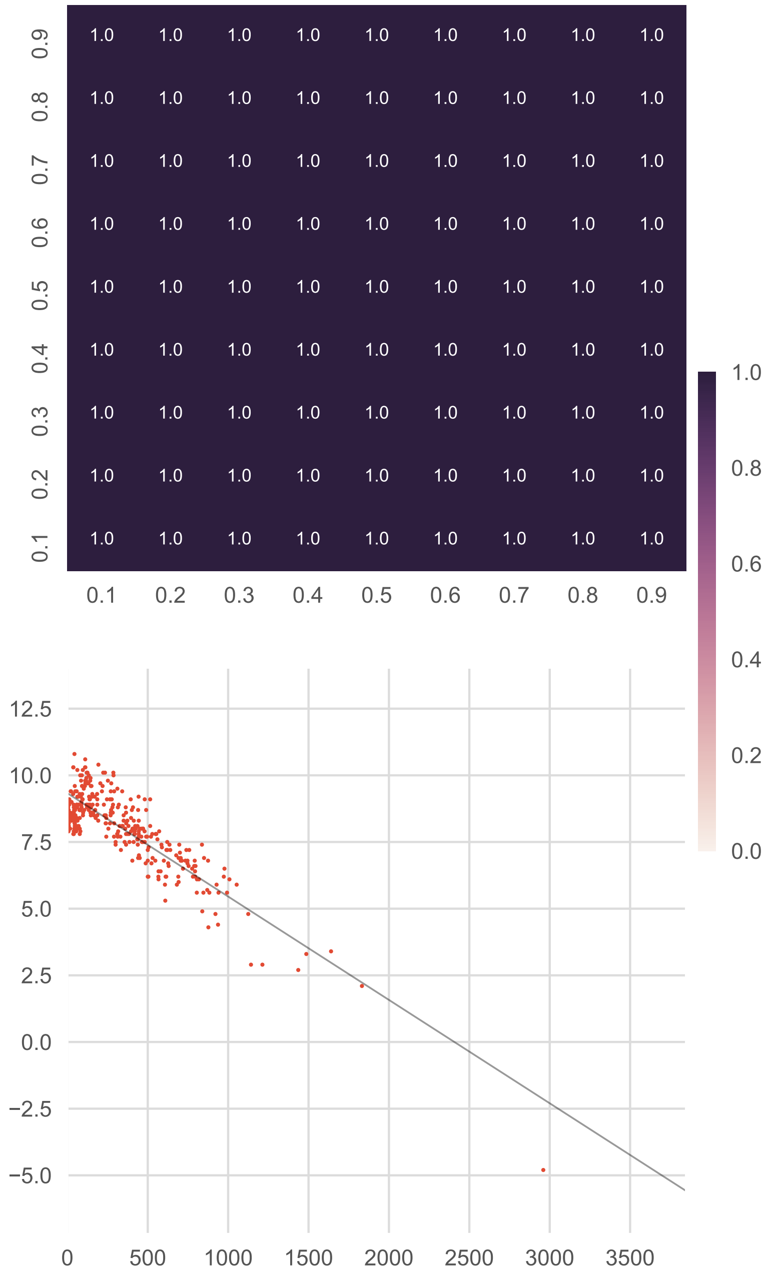

Figure 1: The fitted line was y = 9.330 + -0.004x. The Pearson Correlation Coefficient for the dataset was -0.866 with a p-value of 0.000. The Spearman Rank Correlation Coefficient for the dataset was -0.683. The Kendall Tau Rank Correlation Coefficient for the dataset was -0.500). The normalized mutual information content was 0.436. Causal Direction for this dataset could not be predicted.

2 Dataset-2

x: altitude
y: precipitation (yearly value averaged over 1961-1990)
ground truth:
x --> y

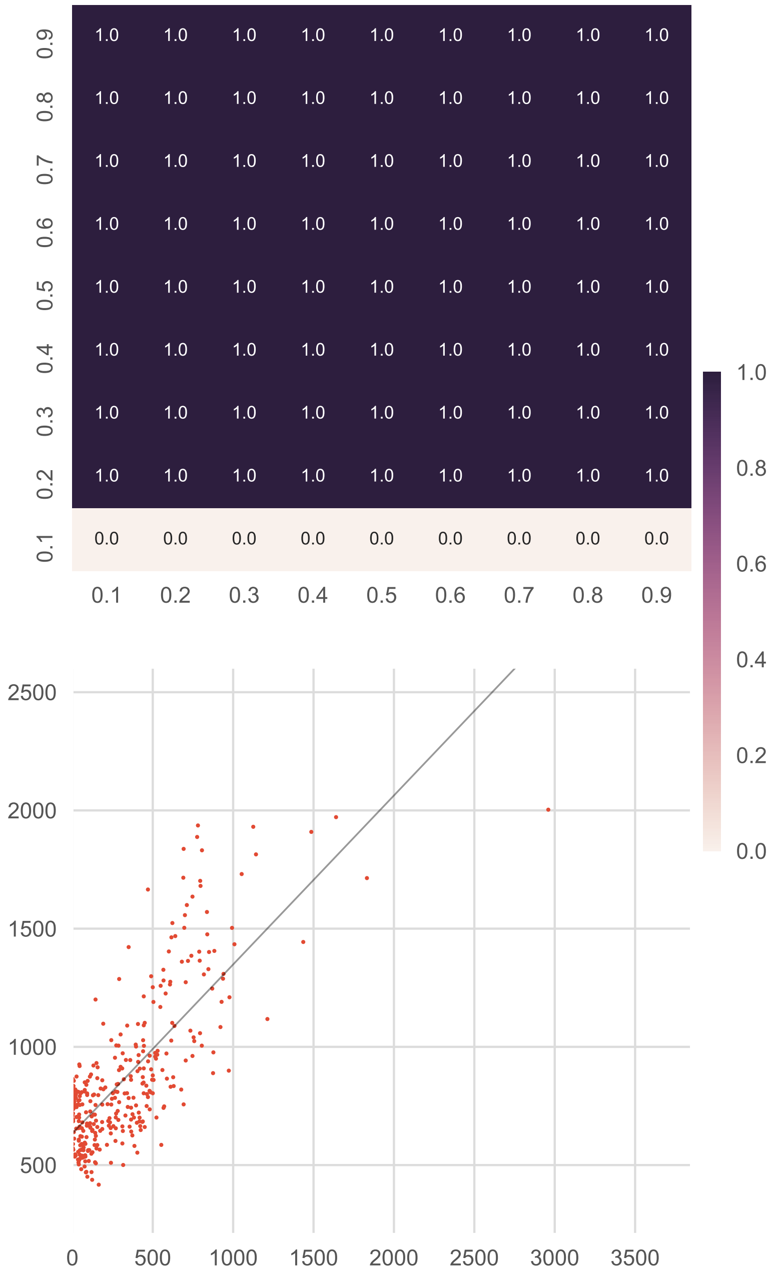

Figure 2: The fitted line was y = 633.878 + 0.715x. The Pearson Correlation Coefficient for the dataset was 0.760 with a p-value of 0.000. The Spearman Rank Correlation Coefficient for the dataset was 0.644. The Kendall Tau Rank Correlation Coefficient for the dataset was 0.455). The normalized mutual information content was 0.490. Causal Direction for this dataset was correctly predicted.

3 Dataset-3

x: longitude
y: temperature (averaged over 1961-1990)
ground truth:
x --> y

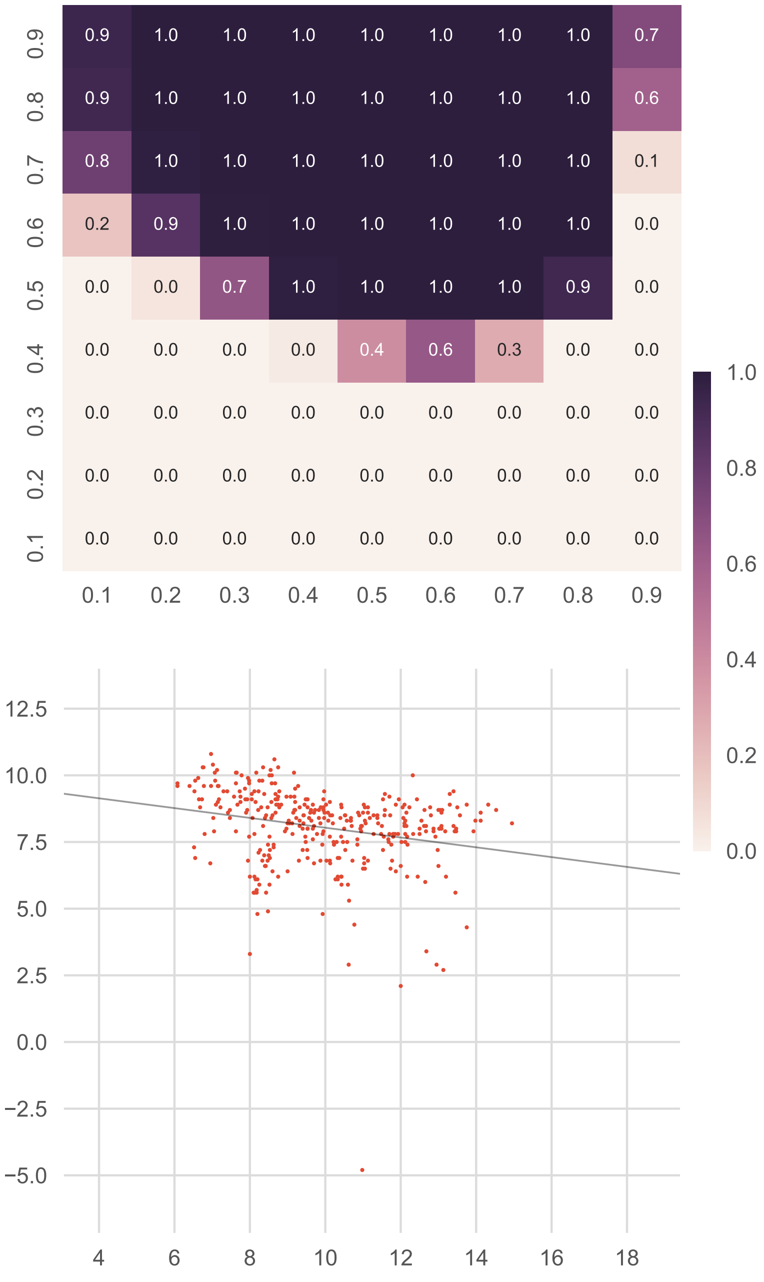

Figure 3: The fitted line was y = 9.873 + -0.184x. The Pearson Correlation Coefficient for the dataset was -0.238 with a p-value of 0.000. The Spearman Rank Correlation Coefficient for the dataset was -0.311. The Kendall Tau Rank Correlation Coefficient for the dataset was -0.211). The normalized mutual information content was 0.504. Causal Direction for this dataset was correctly predicted.

4 Dataset-4

x: altitude
y: sunshine (yearly value averaged over 1961-1990)
ground truth:
x --> y

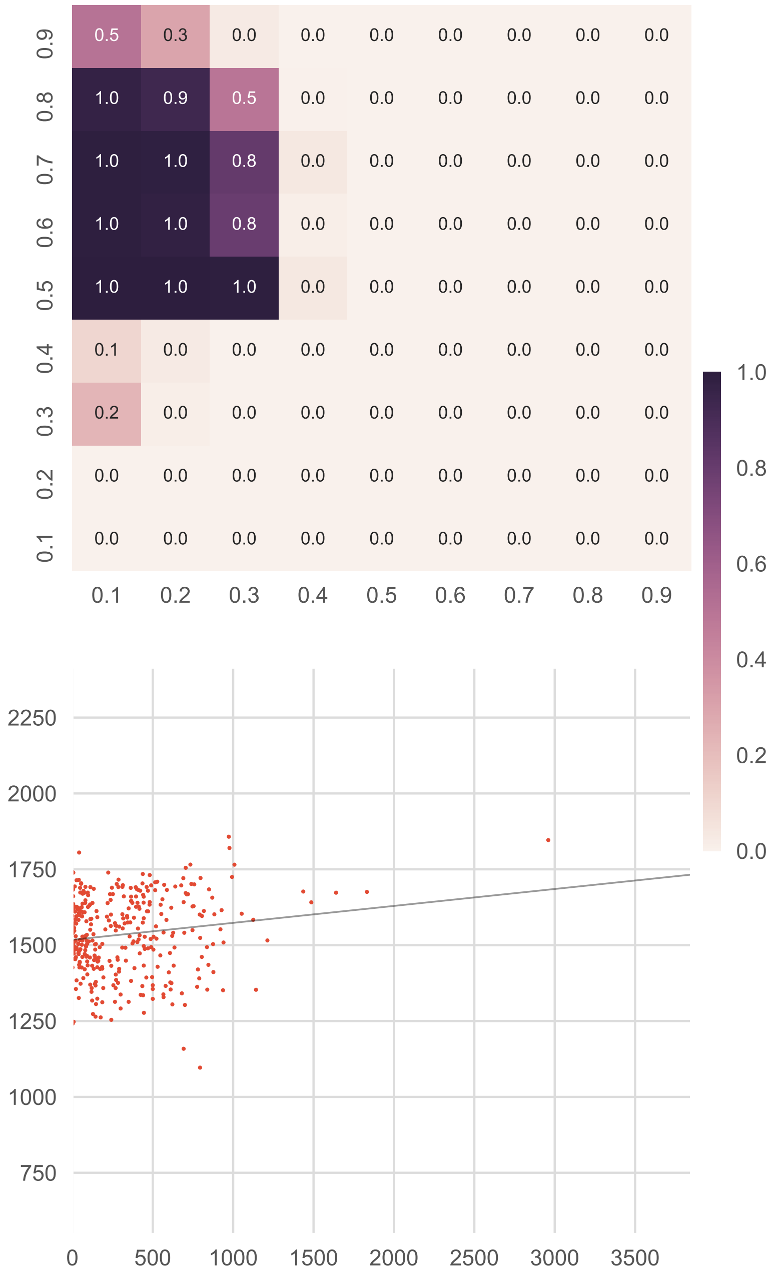

Figure 4: The fitted line was y = 1517.788 + 0.056x. The Pearson Correlation Coefficient for the dataset was 0.148 with a p-value of 0.006. The Spearman Rank Correlation Coefficient for the dataset was 0.074. The Kendall Tau Rank Correlation Coefficient for the dataset was 0.044). The normalized mutual information content was 0.473. Causal Direction for this dataset was incorrectly predicted.

5 Dataset-5

x: Rings integer +1.5 gives the age in years
y: Length continuous mm Longest shell measurement
x --> y

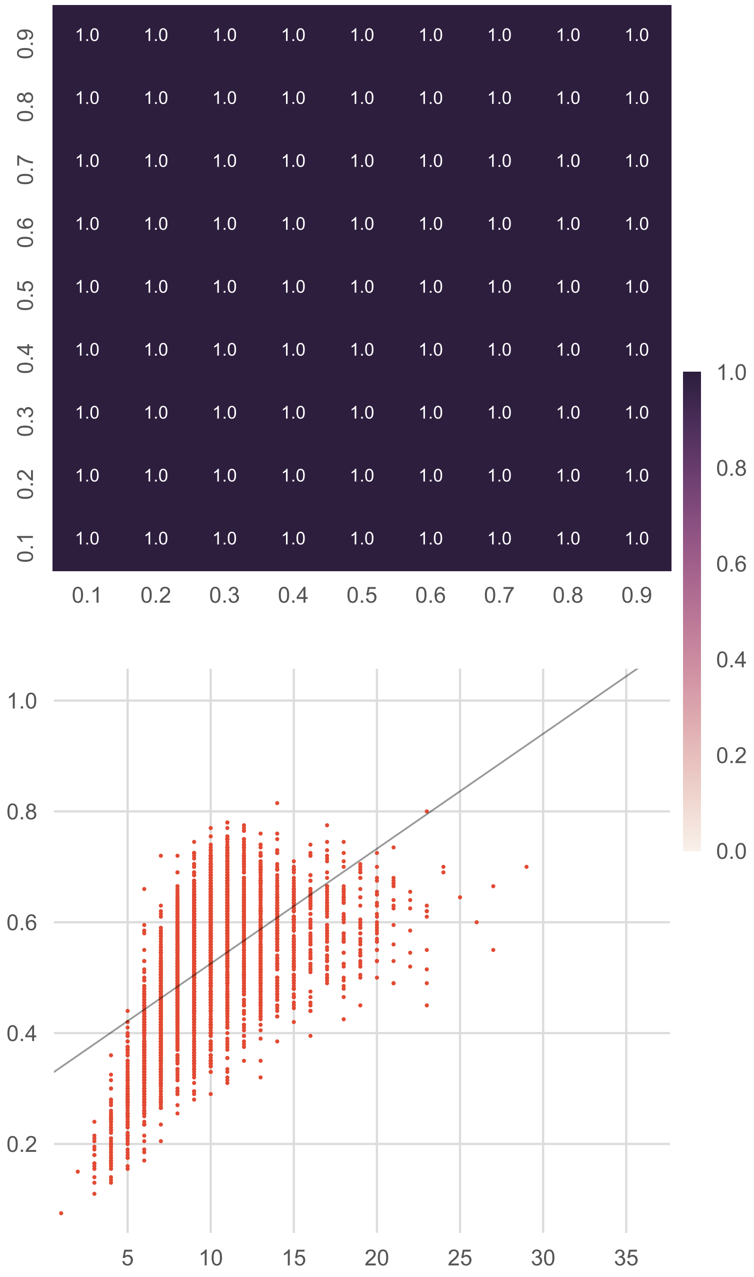

Figure 5: The fitted line was y = 0.318 + 0.021x. The Pearson Correlation Coefficient for the dataset was 0.557 with a p-value of 0.000. The Spearman Rank Correlation Coefficient for the dataset was 0.604. The Kendall Tau Rank Correlation Coefficient for the dataset was 0.457). The normalized mutual information content was 0.156. Causal Direction for this dataset could not be predicted.

6 Dataset-6

x: Rings integer +1.5 gives the age in years
y: Shell weight continuous grams after being dried
x --> y

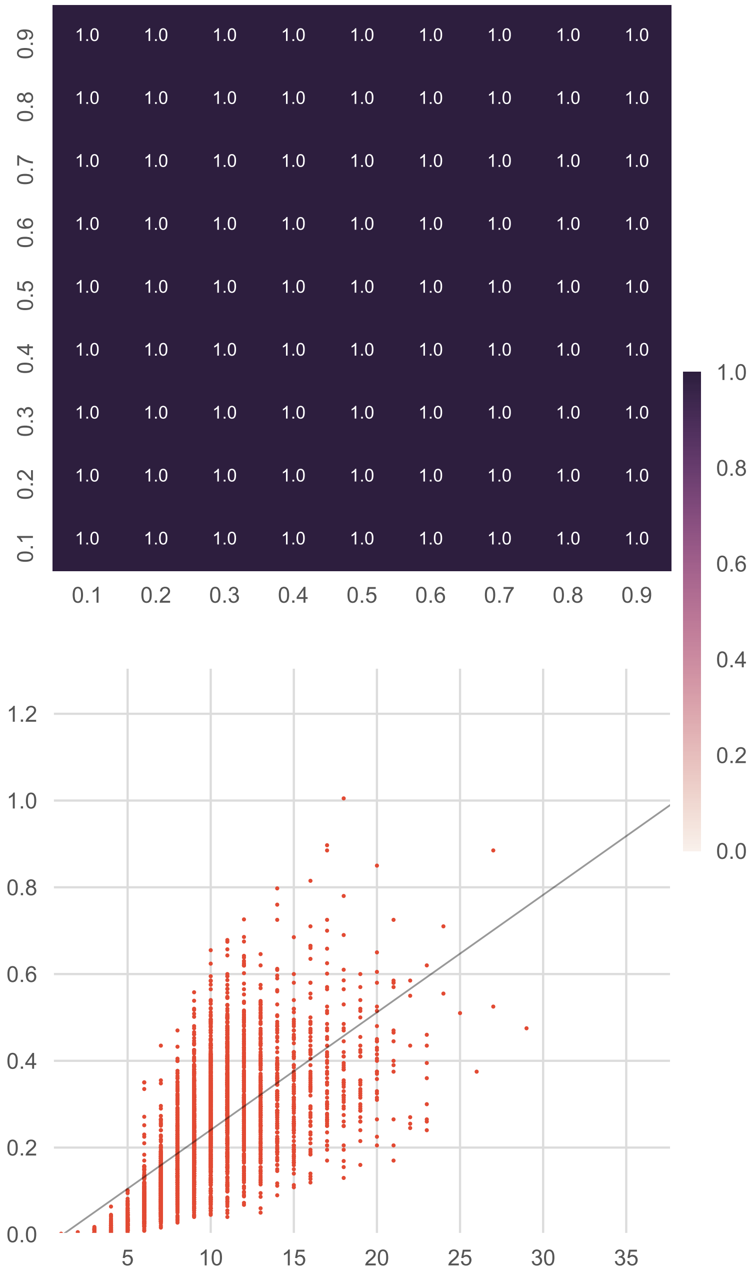

Figure 6: The fitted line was y = -0.030 + 0.027x. The Pearson Correlation Coefficient for the dataset was 0.628 with a p-value of 0.000. The Spearman Rank Correlation Coefficient for the dataset was 0.692. The Kendall Tau Rank Correlation Coefficient for the dataset was 0.535). The normalized mutual information content was 0.181. Causal Direction for this dataset could not be predicted.

7 Dataset-7

x: Rings integer +1.5 gives the age in years
y: Diameter continuous mm perpendicular to length
x --> y

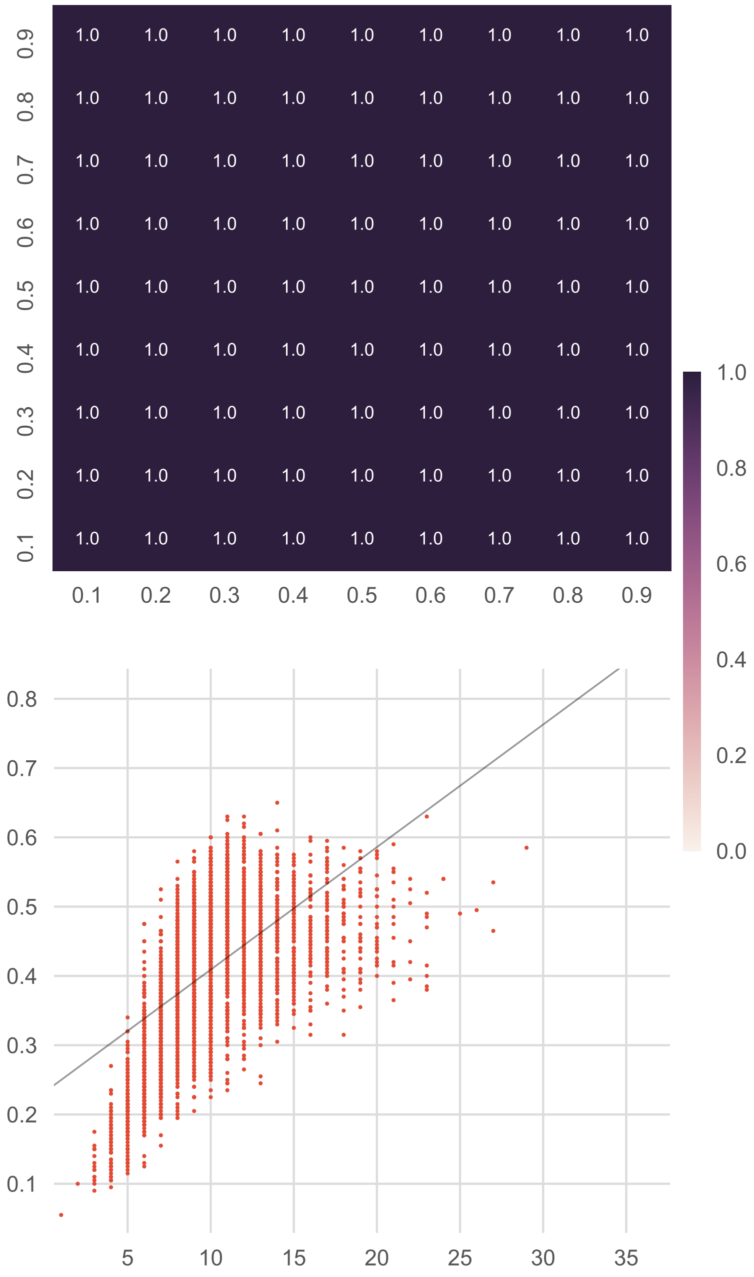

Figure 7: The fitted line was y = 0.232 + 0.018x. The Pearson Correlation Coefficient for the dataset was 0.575 with a p-value of 0.000. The Spearman Rank Correlation Coefficient for the dataset was 0.623. The Kendall Tau Rank Correlation Coefficient for the dataset was 0.474). The normalized mutual information content was 0.164. Causal Direction for this dataset could not be predicted.

8 Dataset-8

x: Rings integer +1.5 gives the age in years
y: Height continuous mm with meat in shell
x --> y

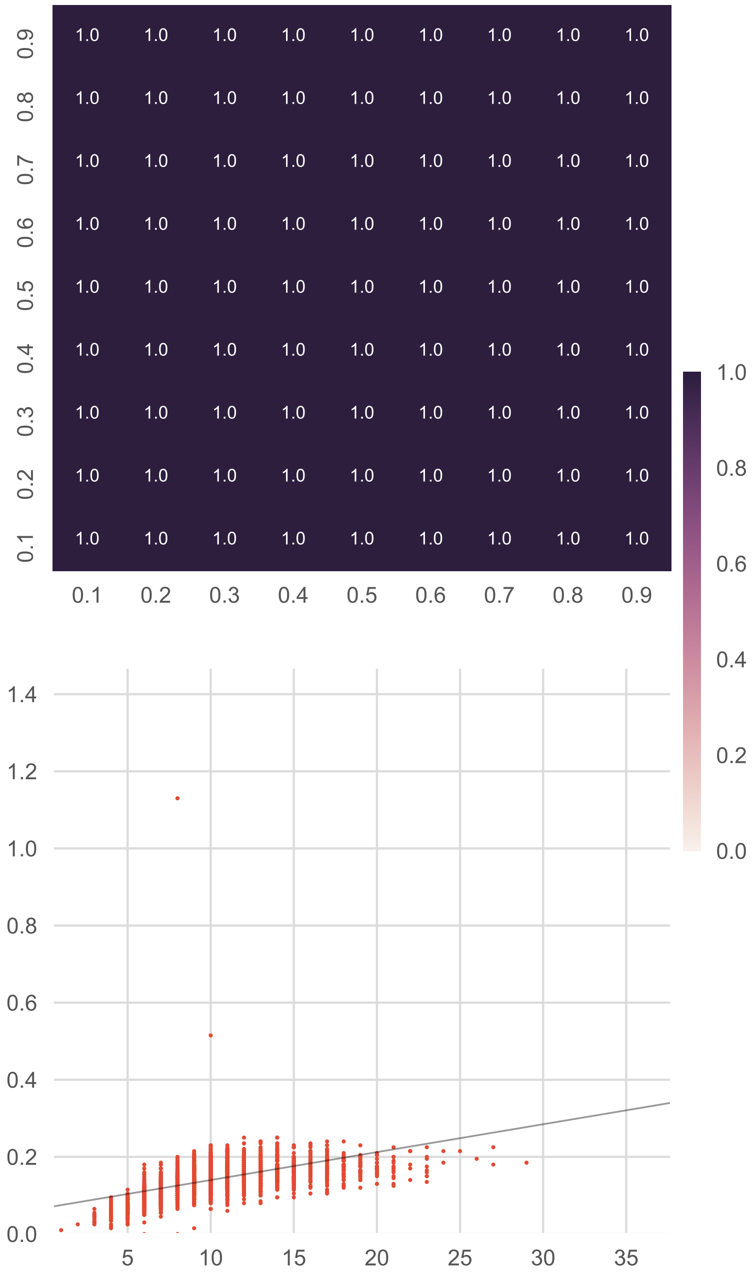

Figure 8: The fitted line was y = 0.068 + 0.007x. The Pearson Correlation Coefficient for the dataset was 0.557 with a p-value of 0.000. The Spearman Rank Correlation Coefficient for the dataset was 0.658. The Kendall Tau Rank Correlation Coefficient for the dataset was 0.509). The normalized mutual information content was 0.165. Causal Direction for this dataset could not be predicted.

9 Dataset-9

x: Rings integer +1.5 gives the age in years
y: Whole weight continuous grams whole abalone
x --> y

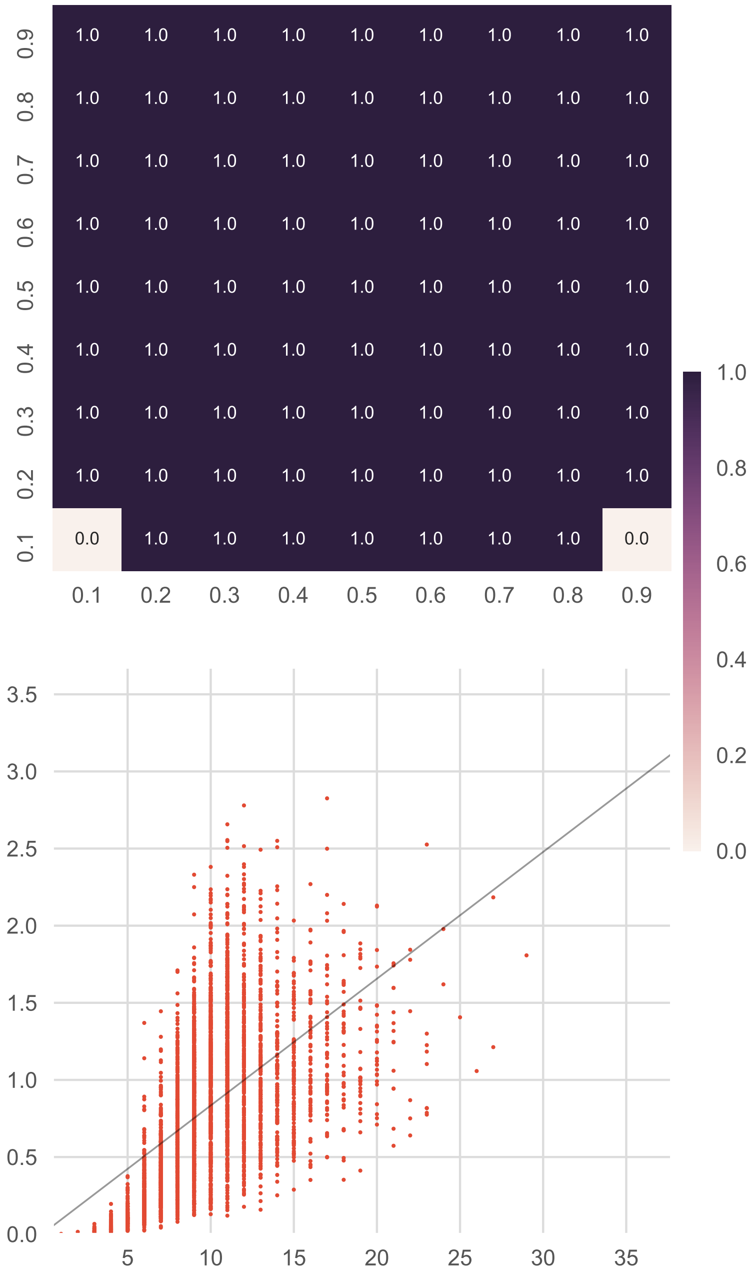

Figure 9: The fitted line was y = 0.012 + 0.082x. The Pearson Correlation Coefficient for the dataset was 0.540 with a p-value of 0.000. The Spearman Rank Correlation Coefficient for the dataset was 0.631. The Kendall Tau Rank Correlation Coefficient for the dataset was 0.476). The normalized mutual information content was 0.166. Causal Direction for this dataset was correctly predicted.

10 Dataset-10

x: Rings integer +1.5 gives the age in years
y: Shucked weight continuous grams weight of meat
x --> y

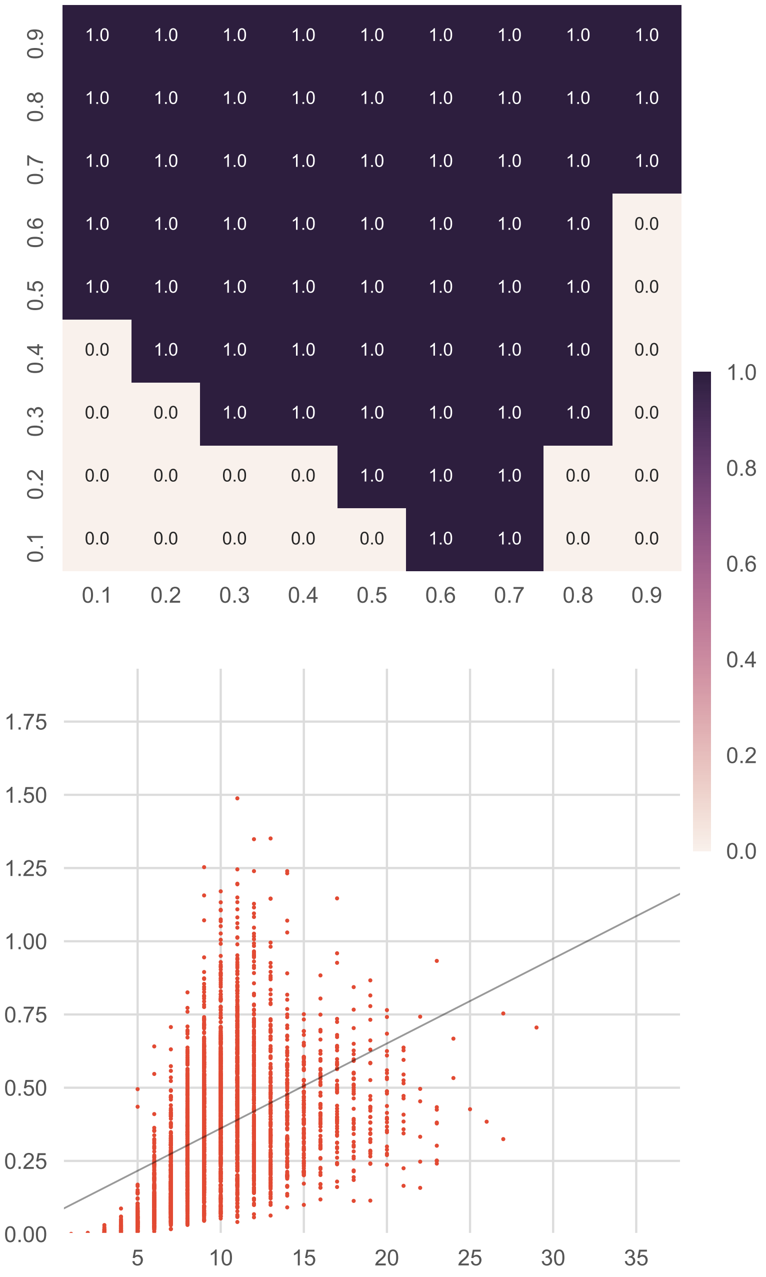

Figure 10: The fitted line was y = 0.072 + 0.029x. The Pearson Correlation Coefficient for the dataset was 0.421 with a p-value of 0.000. The Spearman Rank Correlation Coefficient for the dataset was 0.539. The Kendall Tau Rank Correlation Coefficient for the dataset was 0.399). The normalized mutual information content was 0.146. Causal Direction for this dataset was correctly predicted.

11 Dataset-11

x: Rings integer +1.5 gives the age in years
y: Viscera weight continuous grams gut weight (after bleeding)
x --> y

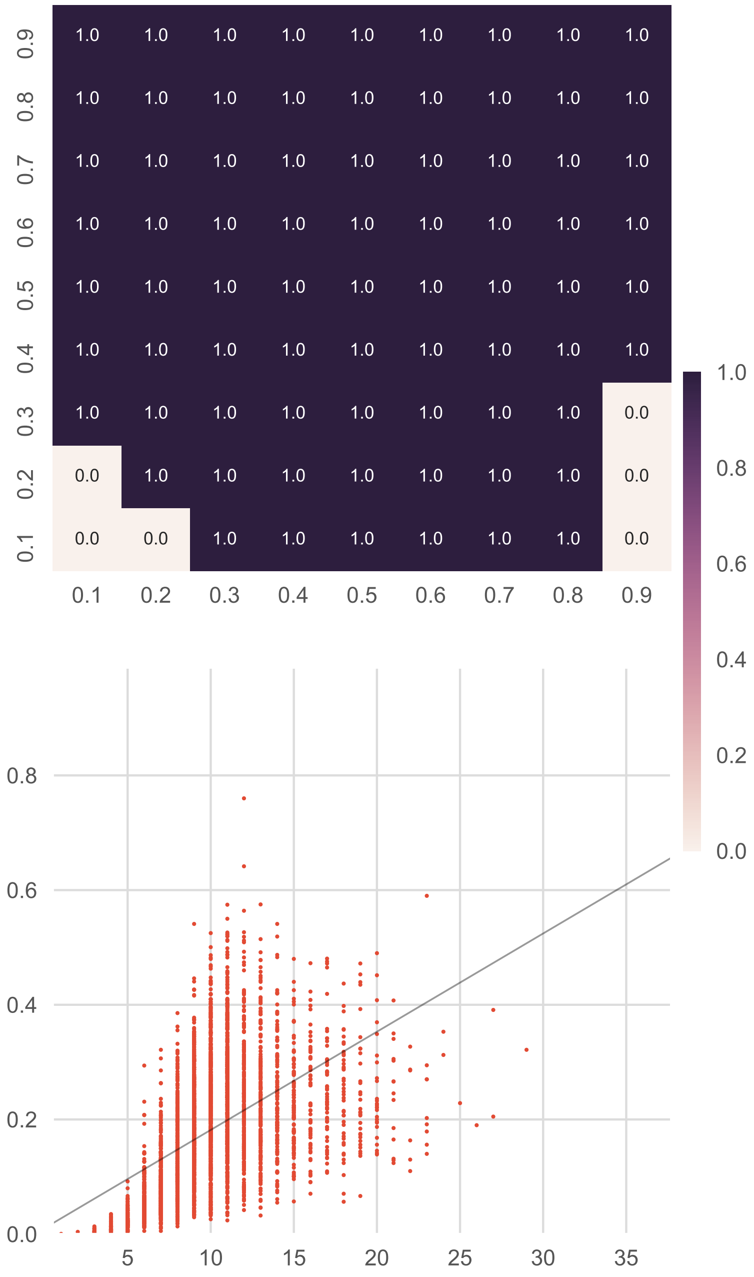

Figure 11: The fitted line was y = 0.010 + 0.017x. The Pearson Correlation Coefficient for the dataset was 0.504 with a p-value of 0.000. The Spearman Rank Correlation Coefficient for the dataset was 0.614. The Kendall Tau Rank Correlation Coefficient for the dataset was 0.461). The normalized mutual information content was 0.158. Causal Direction for this dataset was correctly predicted.

12 Dataset-12

x: displacement
y: mpg
ground truth:
x --> y

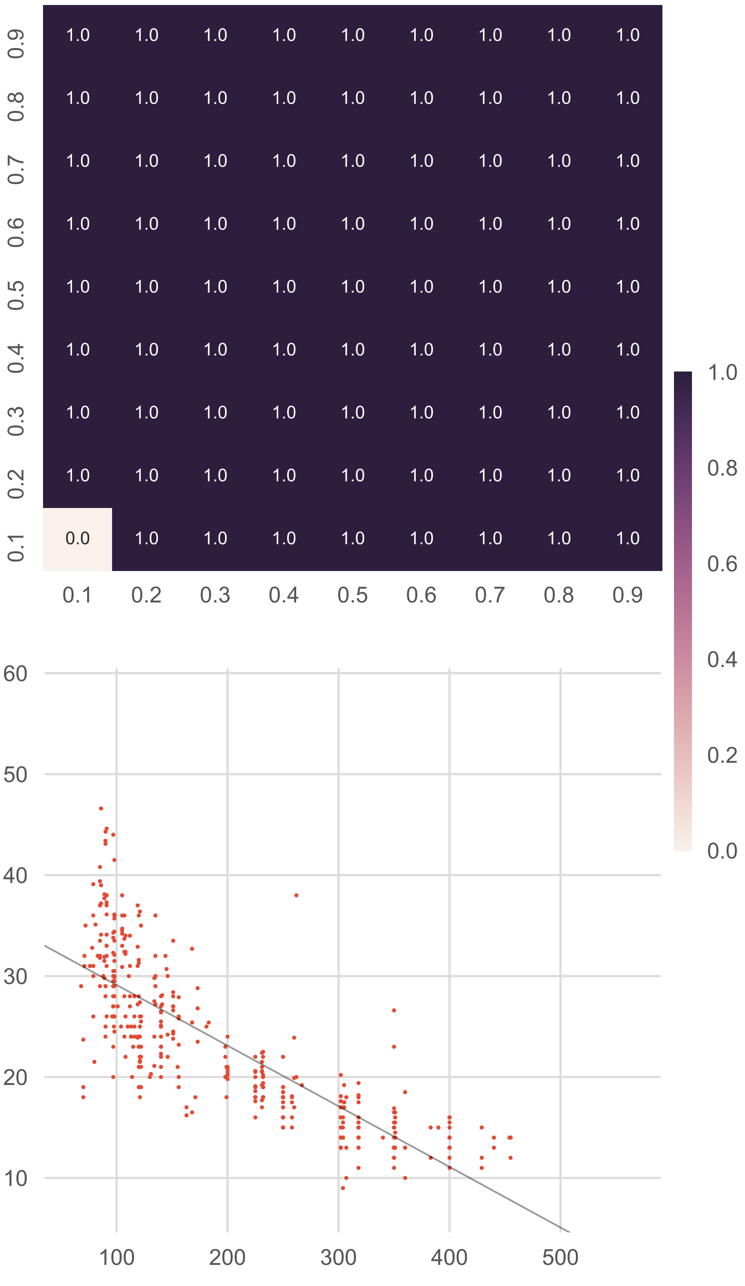

Figure 12: The fitted line was y = 35.121 + -0.060x. The Pearson Correlation Coefficient for the dataset was -0.805 with a p-value of 0.000. The Spearman Rank Correlation Coefficient for the dataset was -0.855. The Kendall Tau Rank Correlation Coefficient for the dataset was -0.679). The normalized mutual information content was 0.500. Causal Direction for this dataset was correctly predicted.

13 Dataset-13

x: horsepower
y: mpg
ground truth:
x --> y

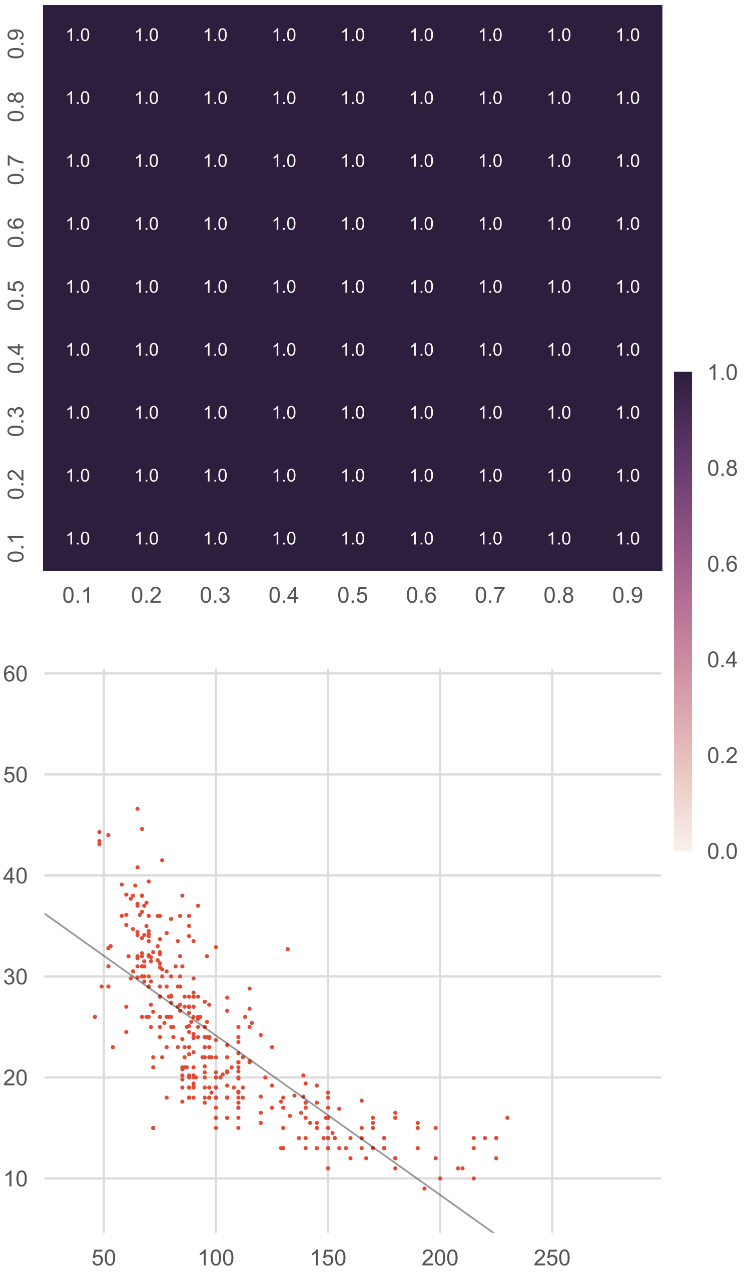

Figure 13: The fitted line was y = 39.936 + -0.158x. The Pearson Correlation Coefficient for the dataset was -0.778 with a p-value of 0.000. The Spearman Rank Correlation Coefficient for the dataset was -0.854. The Kendall Tau Rank Correlation Coefficient for the dataset was -0.679). The normalized mutual information content was 0.542. Causal Direction for this dataset could not be predicted.

14 Dataset-14

x: weight
y: mpg
ground truth:
x --> y

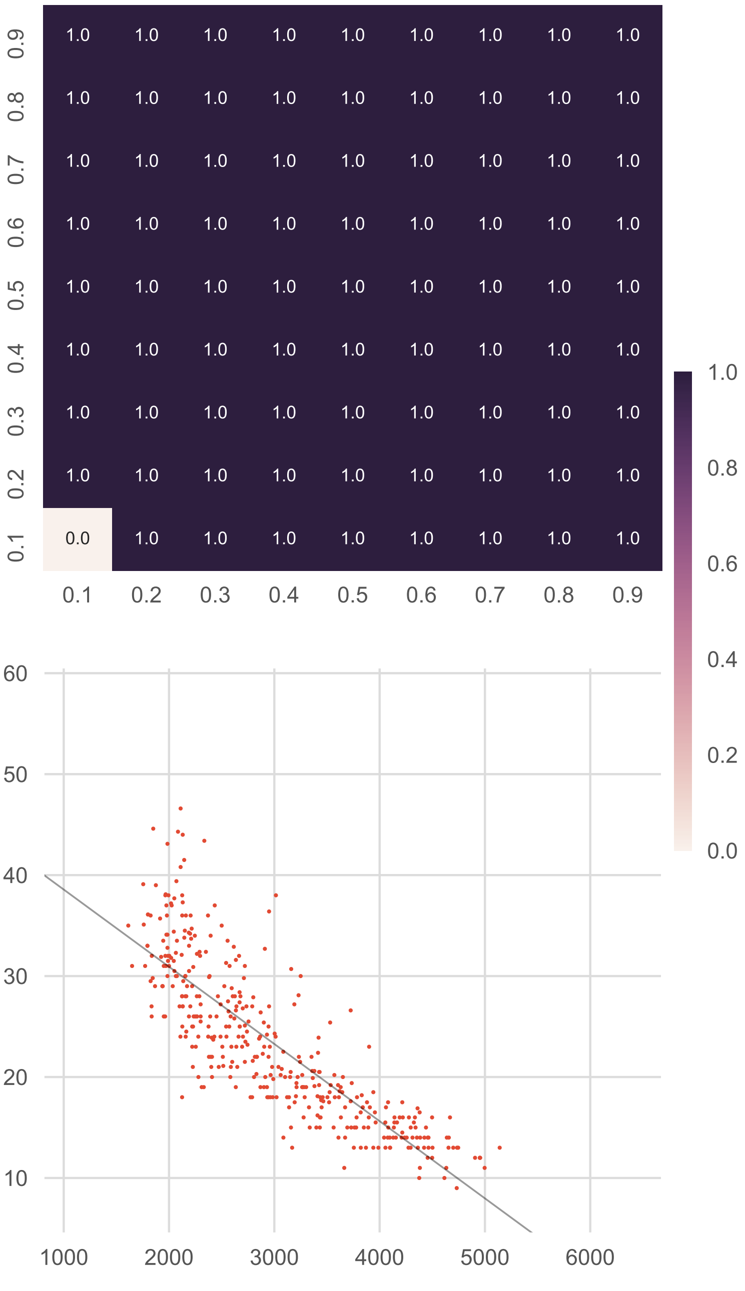

Figure 14: The fitted line was y = 46.217 + -0.008x. The Pearson Correlation Coefficient for the dataset was -0.832 with a p-value of 0.000. The Spearman Rank Correlation Coefficient for the dataset was -0.876. The Kendall Tau Rank Correlation Coefficient for the dataset was -0.694). The normalized mutual information content was 0.585. Causal Direction for this dataset was correctly predicted.

15 Dataset-15

x: horsepower
y: acceleration
ground truth:
x --> y

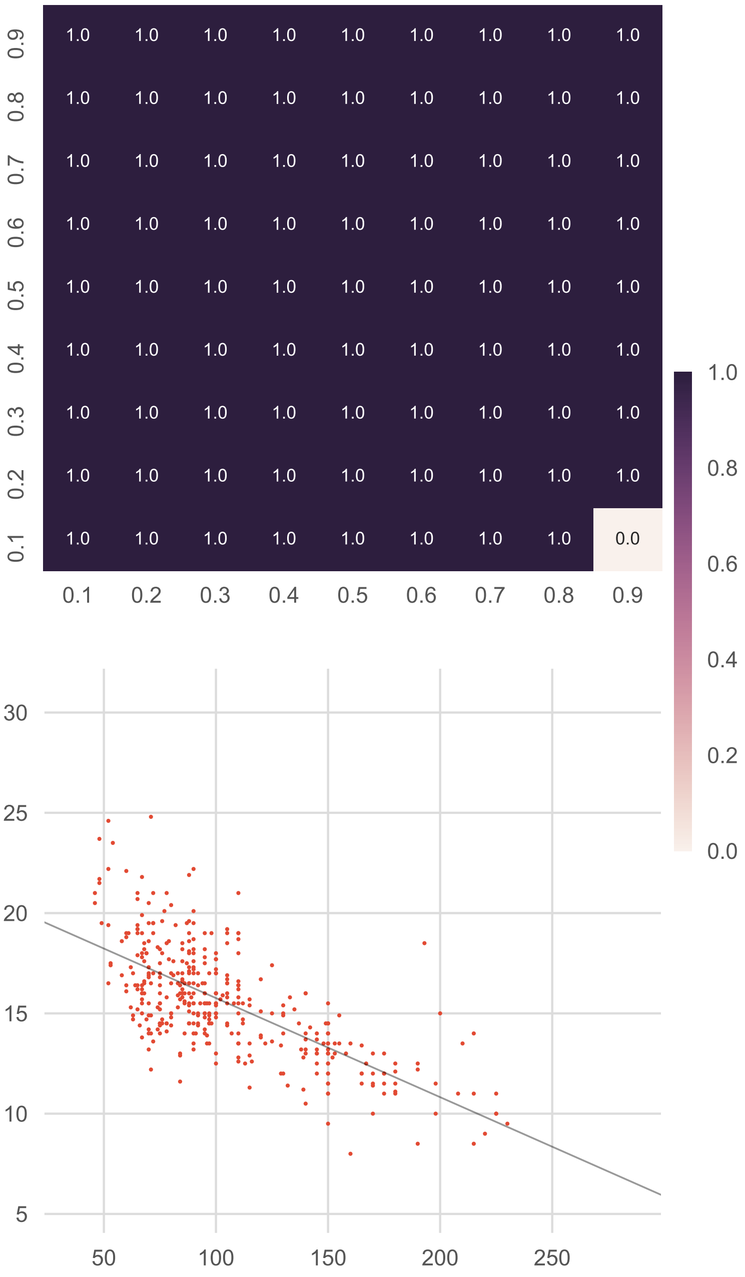

Figure 15: The fitted line was y = 20.702 + -0.049x. The Pearson Correlation Coefficient for the dataset was -0.689 with a p-value of 0.000. The Spearman Rank Correlation Coefficient for the dataset was -0.658. The Kendall Tau Rank Correlation Coefficient for the dataset was -0.488). The normalized mutual information content was 0.497. Causal Direction for this dataset could not be predicted.

16 Dataset-16

x: age of child in years.
y: concentration of GAG (the units have been lost).
ground truth:
x-->y

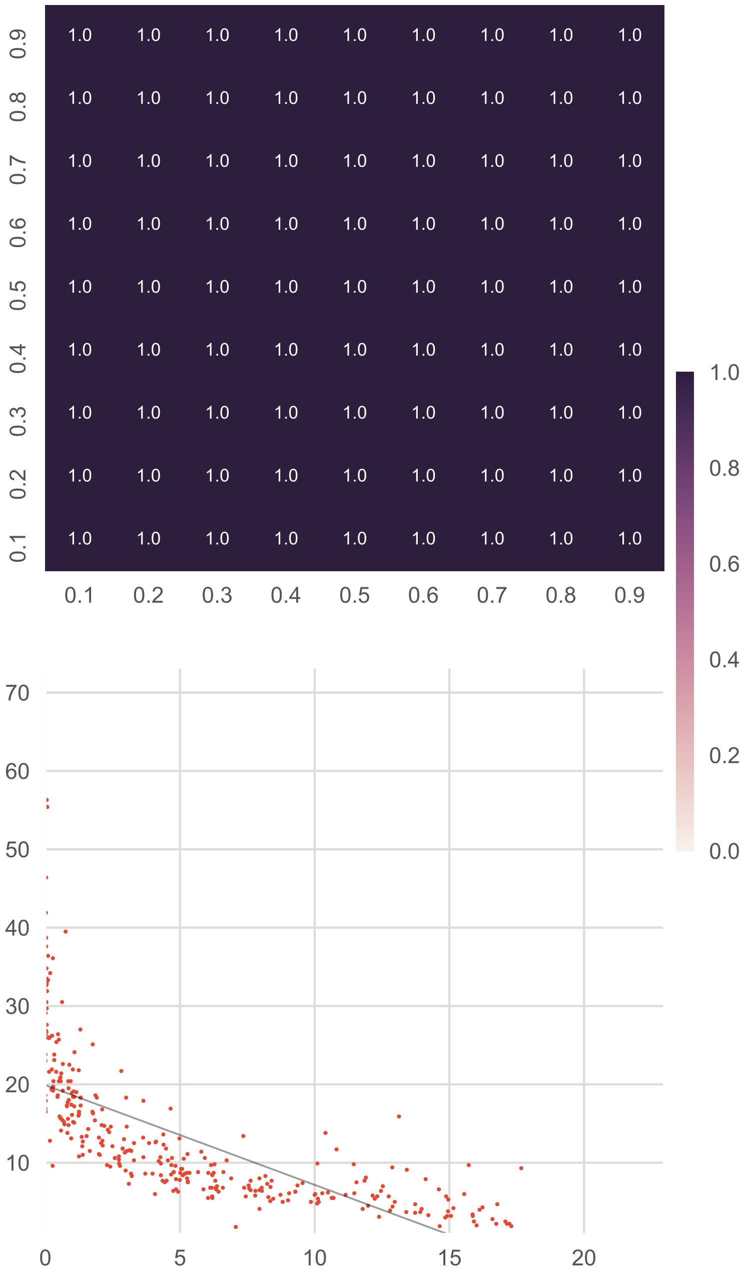

Figure 16: The fitted line was y = 19.894 + -1.273x. The Pearson Correlation Coefficient for the dataset was -0.705 with a p-value of 0.000. The Spearman Rank Correlation Coefficient for the dataset was -0.907. The Kendall Tau Rank Correlation Coefficient for the dataset was -0.743). The normalized mutual information content was 0.599. Causal Direction for this dataset could not be predicted.

17 Dataset-17

x: duration of erruption in minutes
y: time to the next erruption in minutes
ground truth:
x-->y

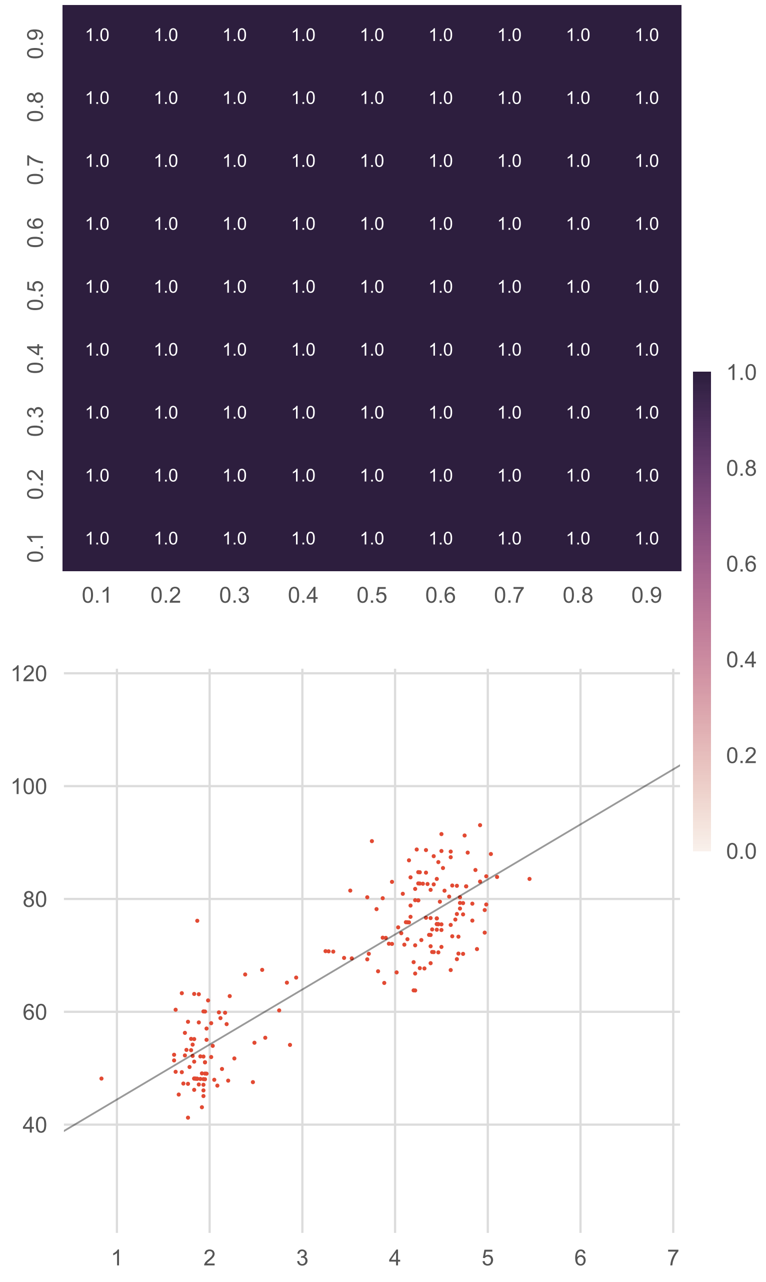

Figure 17: The fitted line was y = 34.689 + 9.755x. The Pearson Correlation Coefficient for the dataset was 0.882 with a p-value of 0.000. The Spearman Rank Correlation Coefficient for the dataset was 0.792. The Kendall Tau Rank Correlation Coefficient for the dataset was 0.575). The normalized mutual information content was 0.717. Causal Direction for this dataset could not be predicted.

18 Dataset-18

x: latitude
y: temperature (averaged over 1961-1990)
ground truth:
x --> y

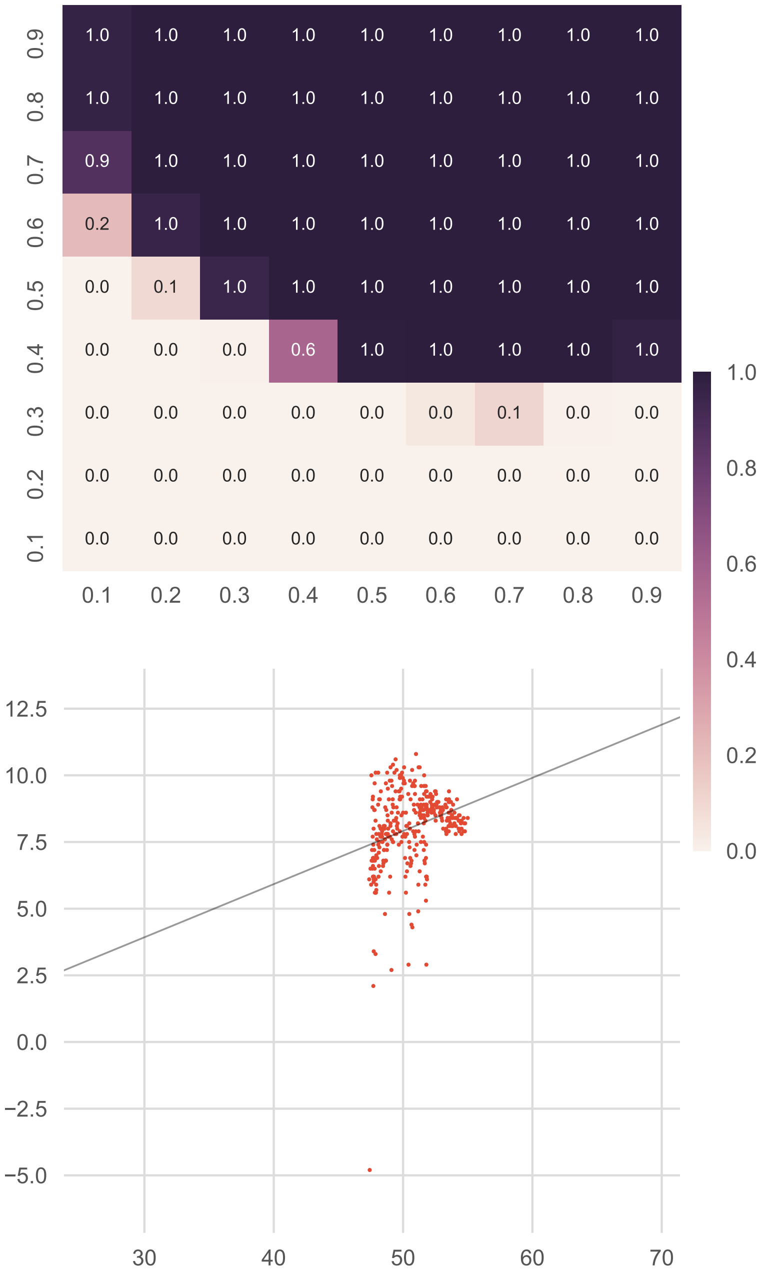

Figure 18: The fitted line was y = -2.053 + 0.199x. The Pearson Correlation Coefficient for the dataset was 0.274 with a p-value of 0.000. The Spearman Rank Correlation Coefficient for the dataset was 0.293. The Kendall Tau Rank Correlation Coefficient for the dataset was 0.177). The normalized mutual information content was 0.529. Causal Direction for this dataset was correctly predicted.

19 Dataset-19

x: longitude
y: precipitation (yearly value averaged over 1961-1990)
ground truth:
x --> y

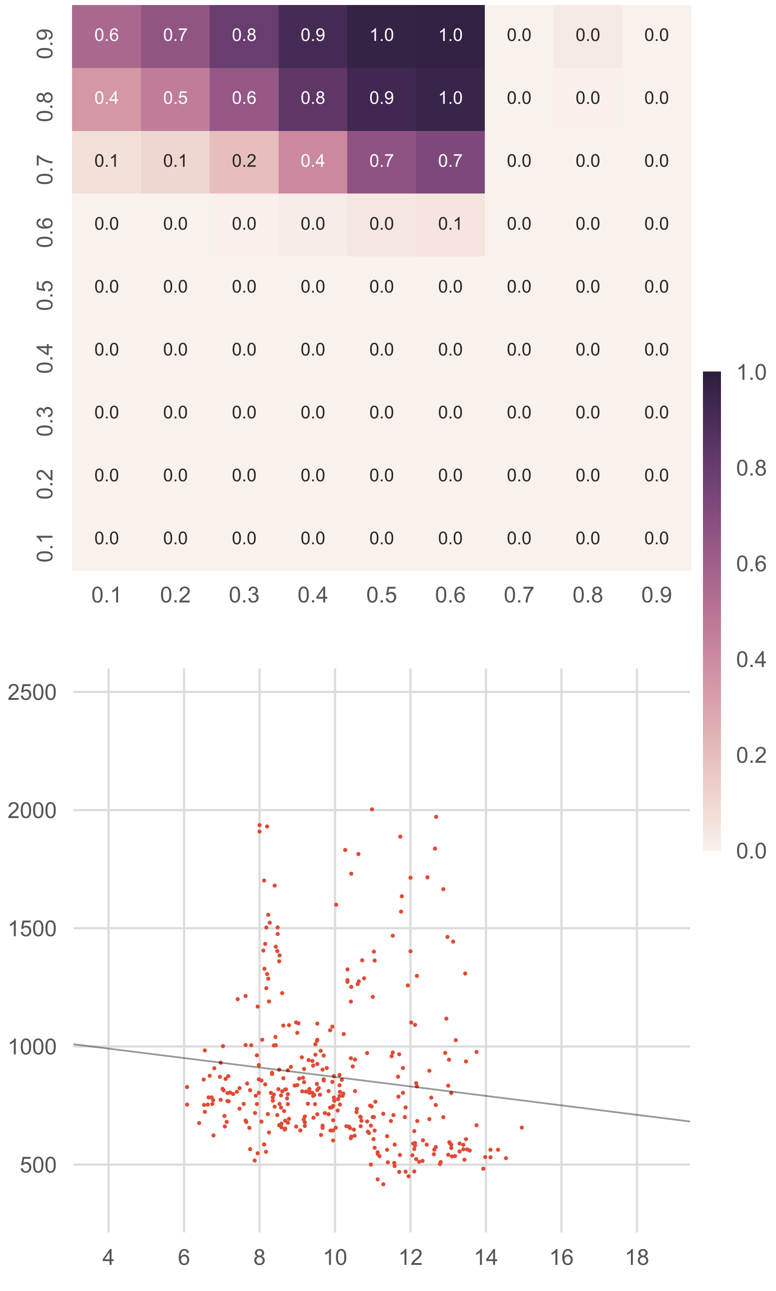

Figure 19: The fitted line was y = 1071.035 + -20.018x. The Pearson Correlation Coefficient for the dataset was -0.123 with a p-value of 0.021. The Spearman Rank Correlation Coefficient for the dataset was -0.261. The Kendall Tau Rank Correlation Coefficient for the dataset was -0.176). The normalized mutual information content was 0.612. Causal Direction for this dataset was correctly predicted.

20 Dataset-20

x: age
y: height
ground truth:
x --> y

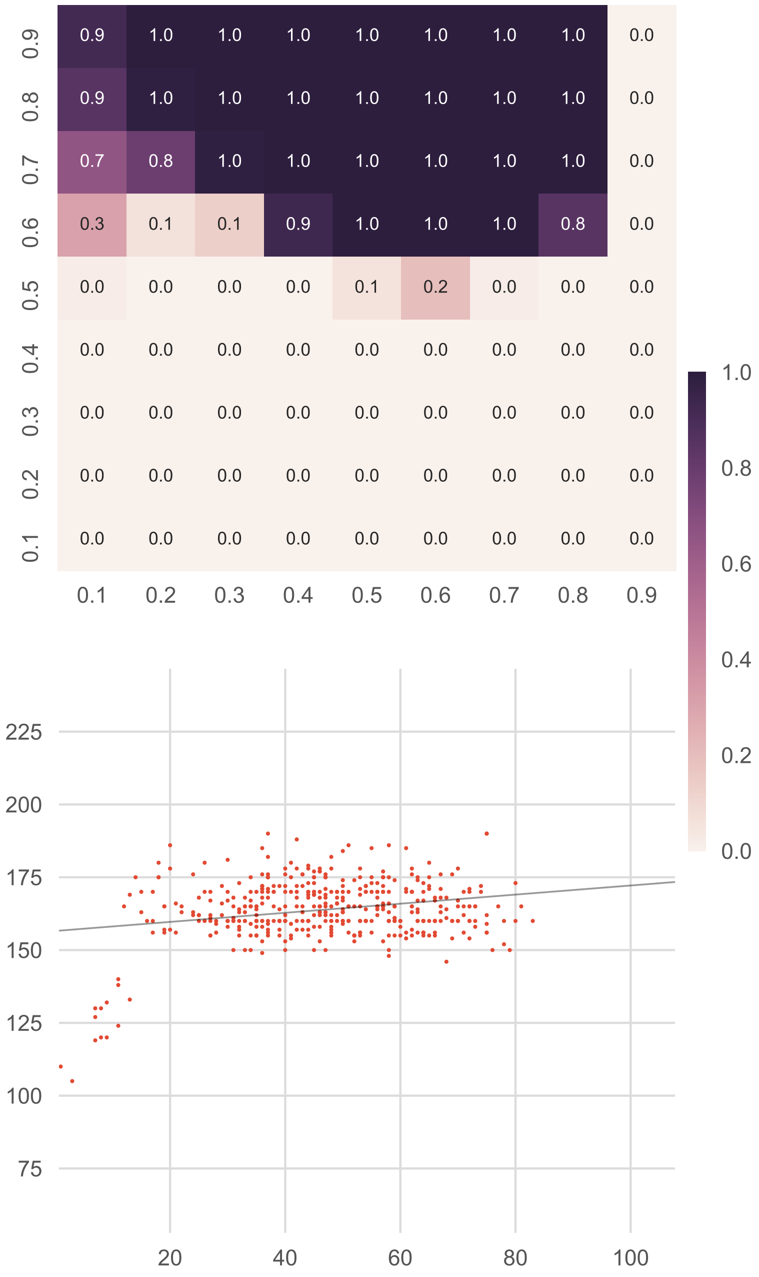

Figure 20: The fitted line was y = 156.550 + 0.156x. The Pearson Correlation Coefficient for the dataset was 0.243 with a p-value of 0.000. The Spearman Rank Correlation Coefficient for the dataset was 0.062. The Kendall Tau Rank Correlation Coefficient for the dataset was 0.042). The normalized mutual information content was 0.425. Causal Direction for this dataset was correctly predicted.

21 Dataset-21

x: age
y: weight
ground truth:
x --> y

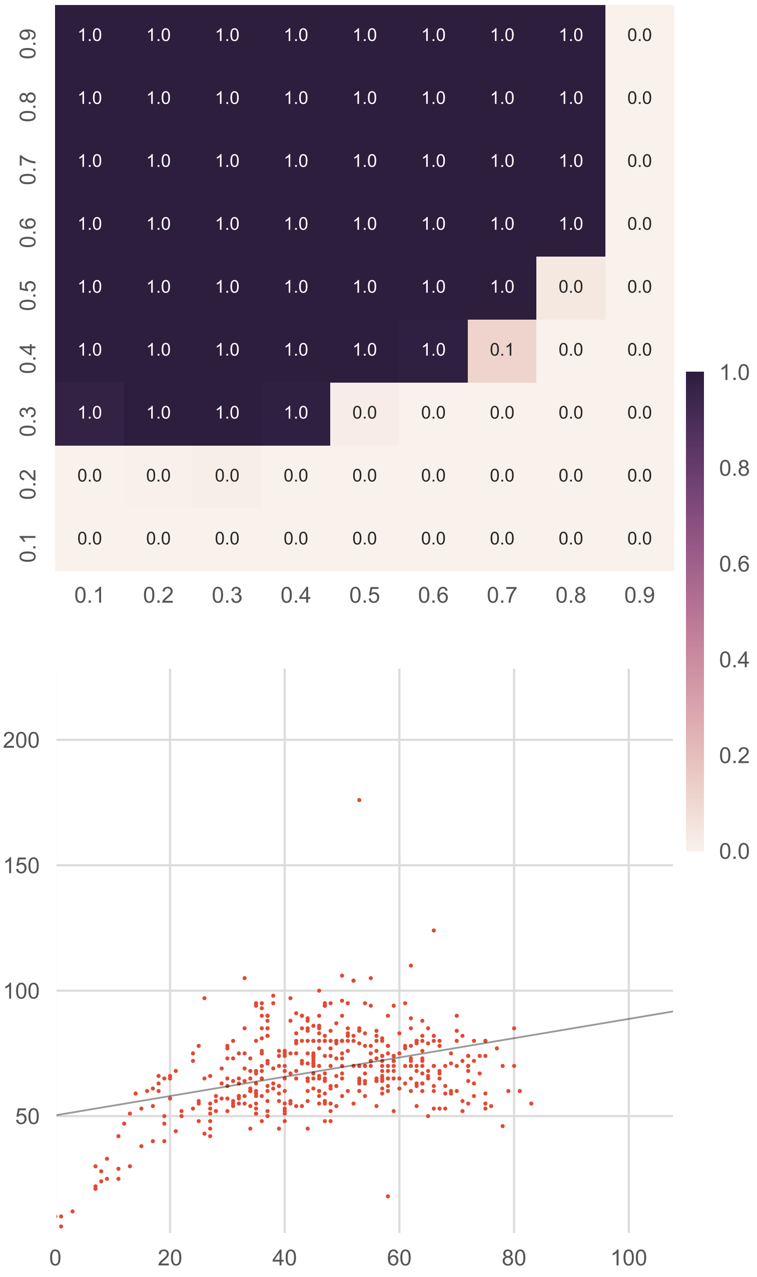

Figure 21: The fitted line was y = 50.305 + 0.384x. The Pearson Correlation Coefficient for the dataset was 0.382 with a p-value of 0.000. The Spearman Rank Correlation Coefficient for the dataset was 0.295. The Kendall Tau Rank Correlation Coefficient for the dataset was 0.201). The normalized mutual information content was 0.432. Causal Direction for this dataset was correctly predicted.

22 Dataset-22

x: age
y: heart rate
ground truth:
x --> y

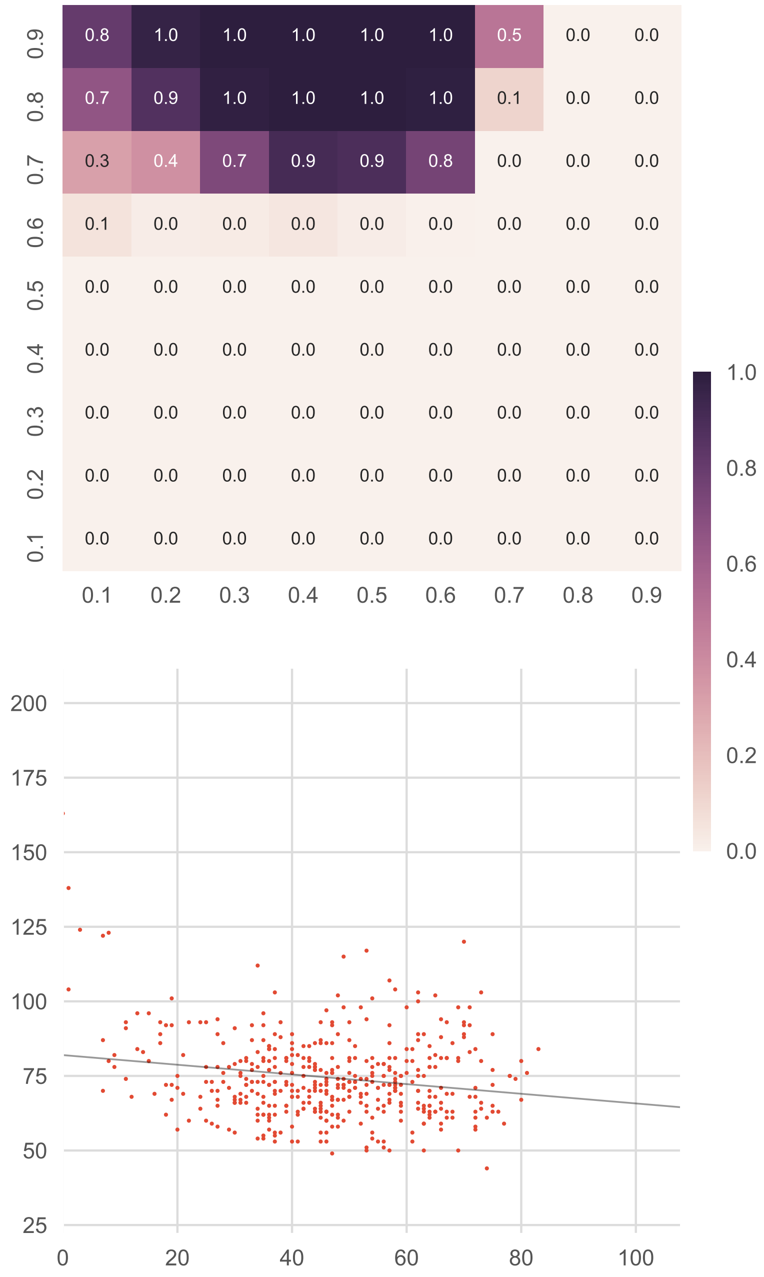

Figure 22: The fitted line was y = 81.989 + -0.162x. The Pearson Correlation Coefficient for the dataset was -0.192 with a p-value of 0.000. The Spearman Rank Correlation Coefficient for the dataset was -0.098. The Kendall Tau Rank Correlation Coefficient for the dataset was -0.069). The normalized mutual information content was 0.457. Causal Direction for this dataset was correctly predicted.

23 Dataset-23

x: blast furnace slag
y: compressive strength
ground truth:
x --> y

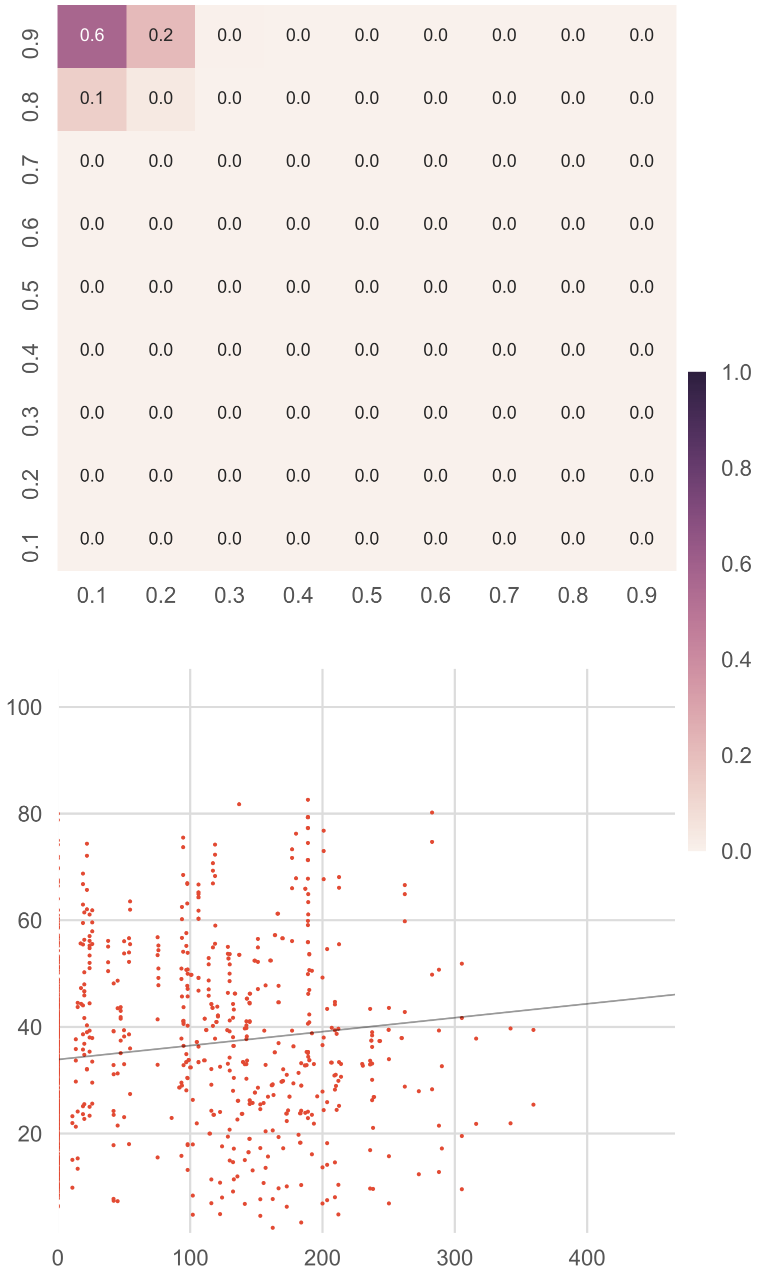

Figure 23: The fitted line was y = 33.889 + 0.026x. The Pearson Correlation Coefficient for the dataset was 0.135 with a p-value of 0.000. The Spearman Rank Correlation Coefficient for the dataset was 0.164. The Kendall Tau Rank Correlation Coefficient for the dataset was 0.119). The normalized mutual information content was 0.366. Causal Direction for this dataset could not be predicted.

24 Dataset-24

x: fly ash
y: compressive strength
ground truth:
x --> y

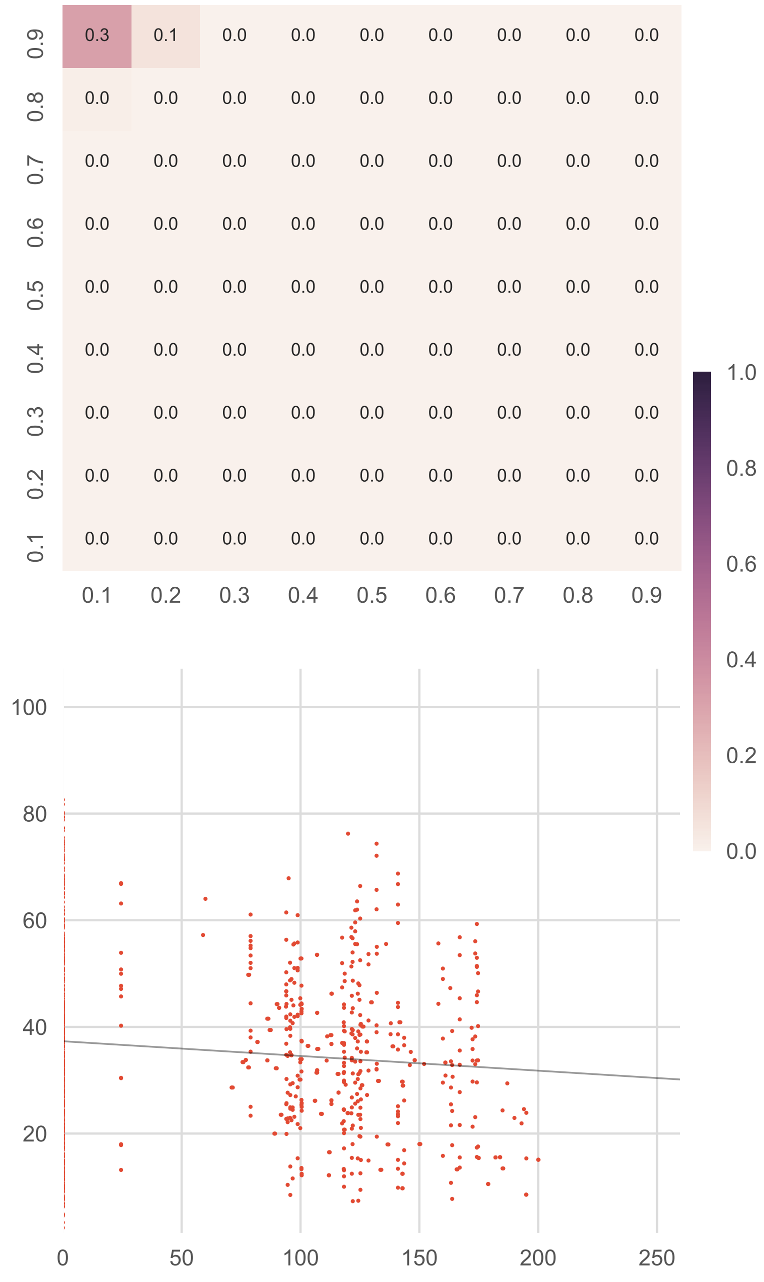

Figure 24: The fitted line was y = 37.314 + -0.028x. The Pearson Correlation Coefficient for the dataset was -0.106 with a p-value of 0.001. The Spearman Rank Correlation Coefficient for the dataset was -0.078. The Kendall Tau Rank Correlation Coefficient for the dataset was -0.060). The normalized mutual information content was 0.296. Causal Direction for this dataset could not be predicted.

25 Dataset-25

x: water
y: compressive strength
ground truth:
x --> y

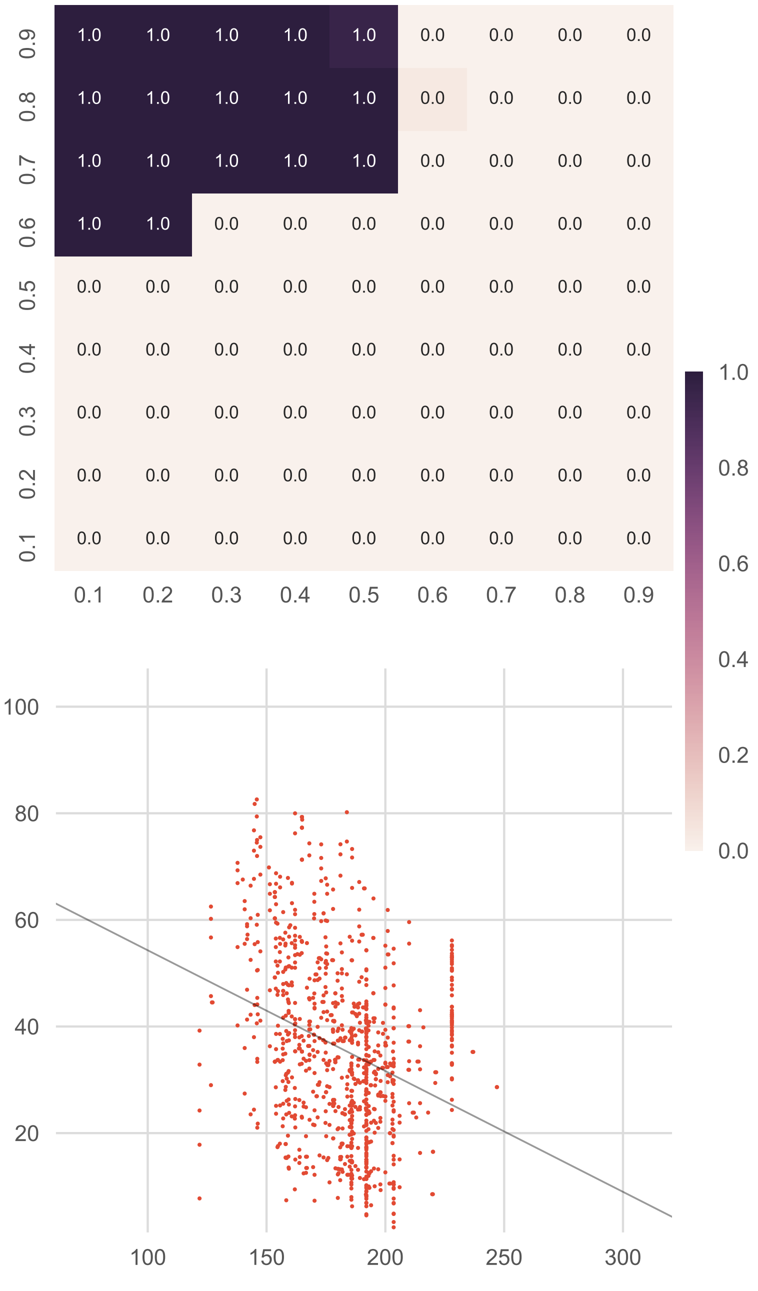

Figure 25: The fitted line was y = 76.958 + -0.227x. The Pearson Correlation Coefficient for the dataset was -0.290 with a p-value of 0.000. The Spearman Rank Correlation Coefficient for the dataset was -0.308. The Kendall Tau Rank Correlation Coefficient for the dataset was -0.206). The normalized mutual information content was 0.392. Causal Direction for this dataset was correctly predicted.

26 Dataset-26

x: superplasticizer
y: compressive strength
ground truth:
x --> y

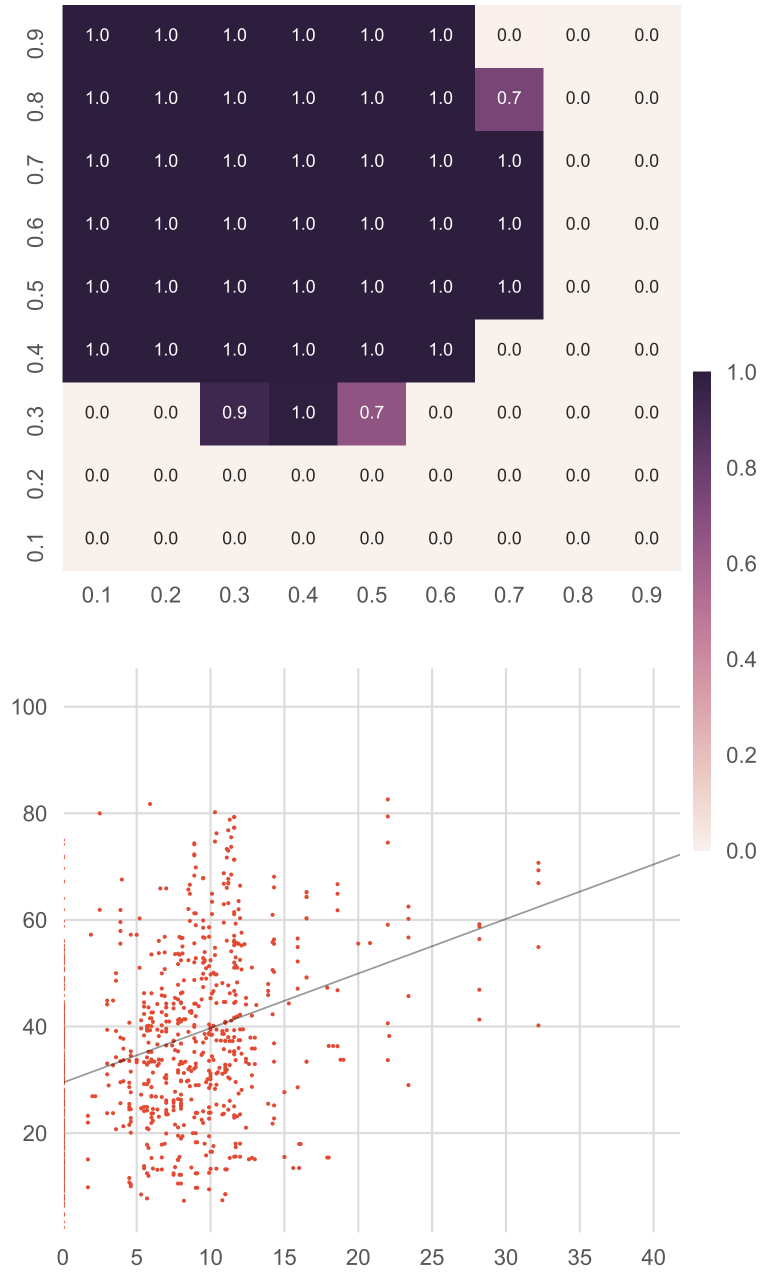

Figure 26: The fitted line was y = 29.466 + 1.024x. The Pearson Correlation Coefficient for the dataset was 0.366 with a p-value of 0.000. The Spearman Rank Correlation Coefficient for the dataset was 0.348. The Kendall Tau Rank Correlation Coefficient for the dataset was 0.250). The normalized mutual information content was 0.331. Causal Direction for this dataset was correctly predicted.

27 Dataset-27

x: coarse aggregate
y: compressive strength
ground truth:
x --> y

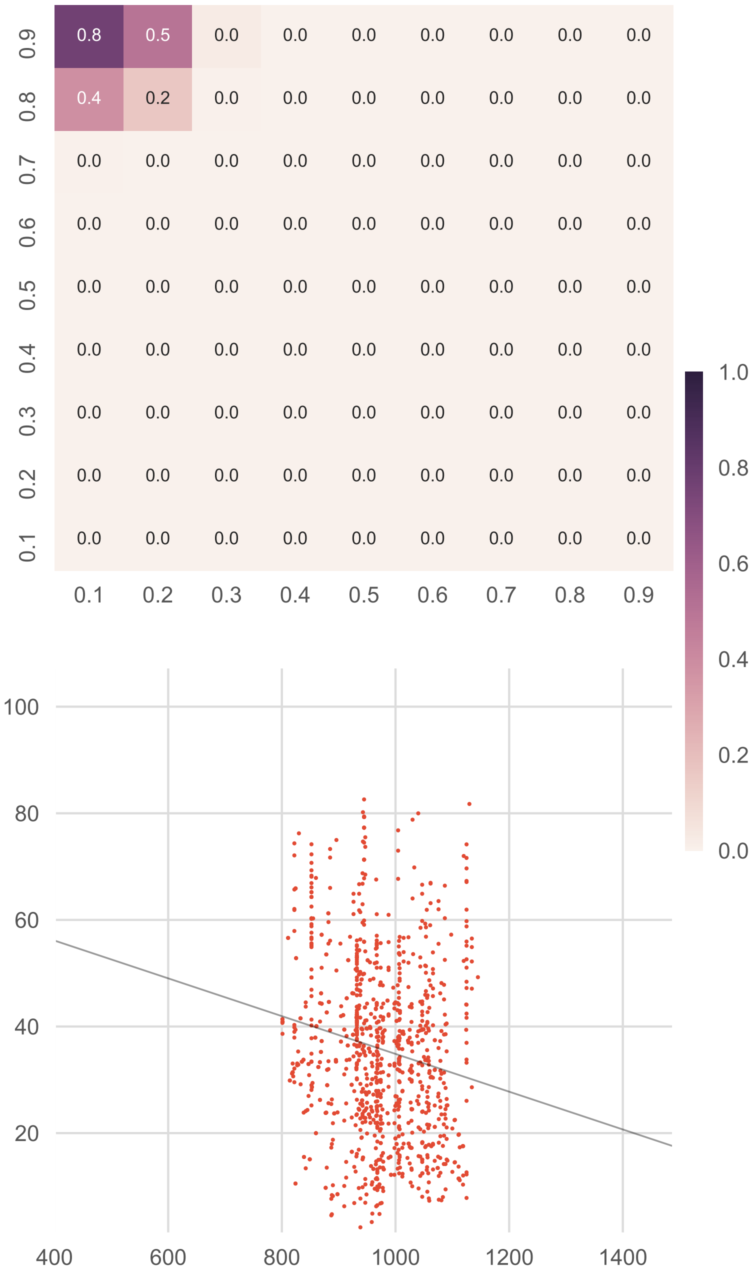

Figure 27: The fitted line was y = 70.295 + -0.035x. The Pearson Correlation Coefficient for the dataset was -0.165 with a p-value of 0.000. The Spearman Rank Correlation Coefficient for the dataset was -0.184. The Kendall Tau Rank Correlation Coefficient for the dataset was -0.124). The normalized mutual information content was 0.441. Causal Direction for this dataset was correctly predicted.

28 Dataset-28

x: drinks number of half-pint equivalents of alcoholic beverages
y: mcv mean corpuscular volume
ground truth:
x-->y

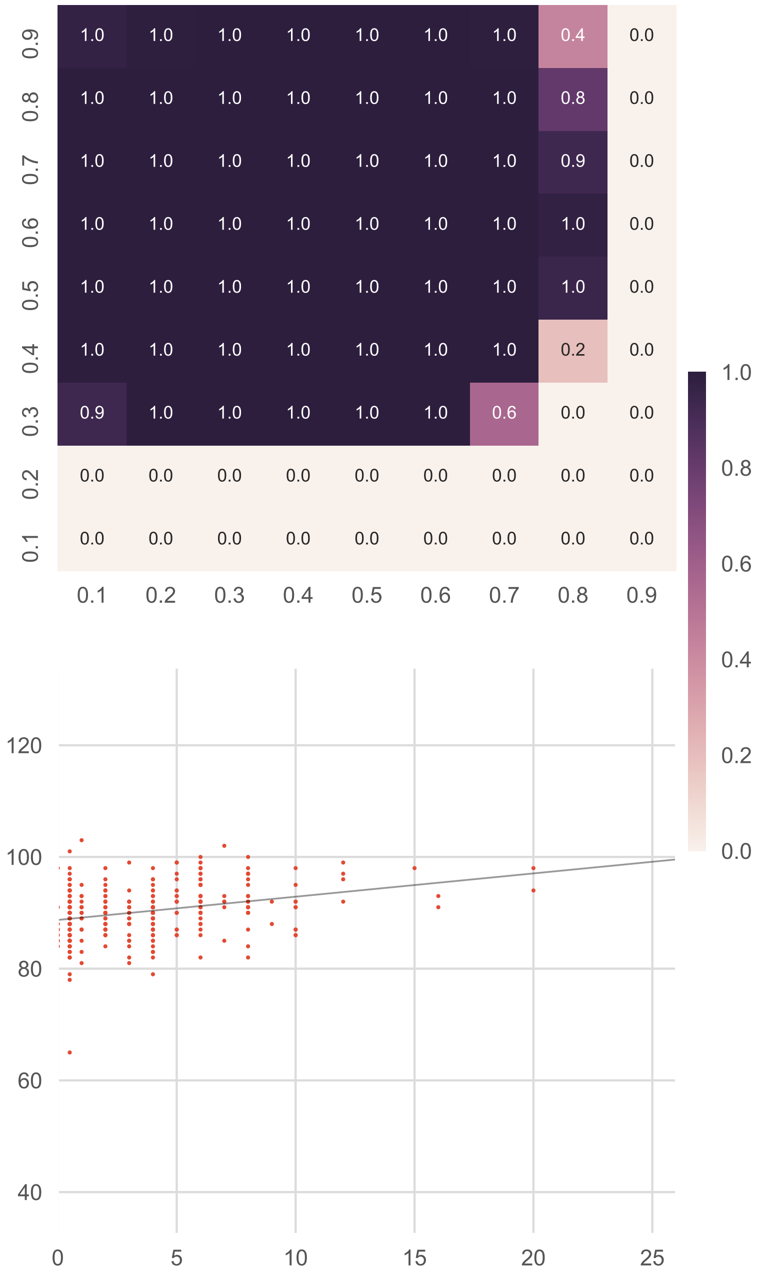

Figure 28: The fitted line was y = 88.720 + 0.417x. The Pearson Correlation Coefficient for the dataset was 0.313 with a p-value of 0.000. The Spearman Rank Correlation Coefficient for the dataset was 0.320. The Kendall Tau Rank Correlation Coefficient for the dataset was 0.240). The normalized mutual information content was 0.168. Causal Direction for this dataset was correctly predicted.

29 Dataset-29

x: drinks number of half-pint equivalents of alcoholic beverages
y: alkphos alkaline phosphotase
ground truth:
x-->y

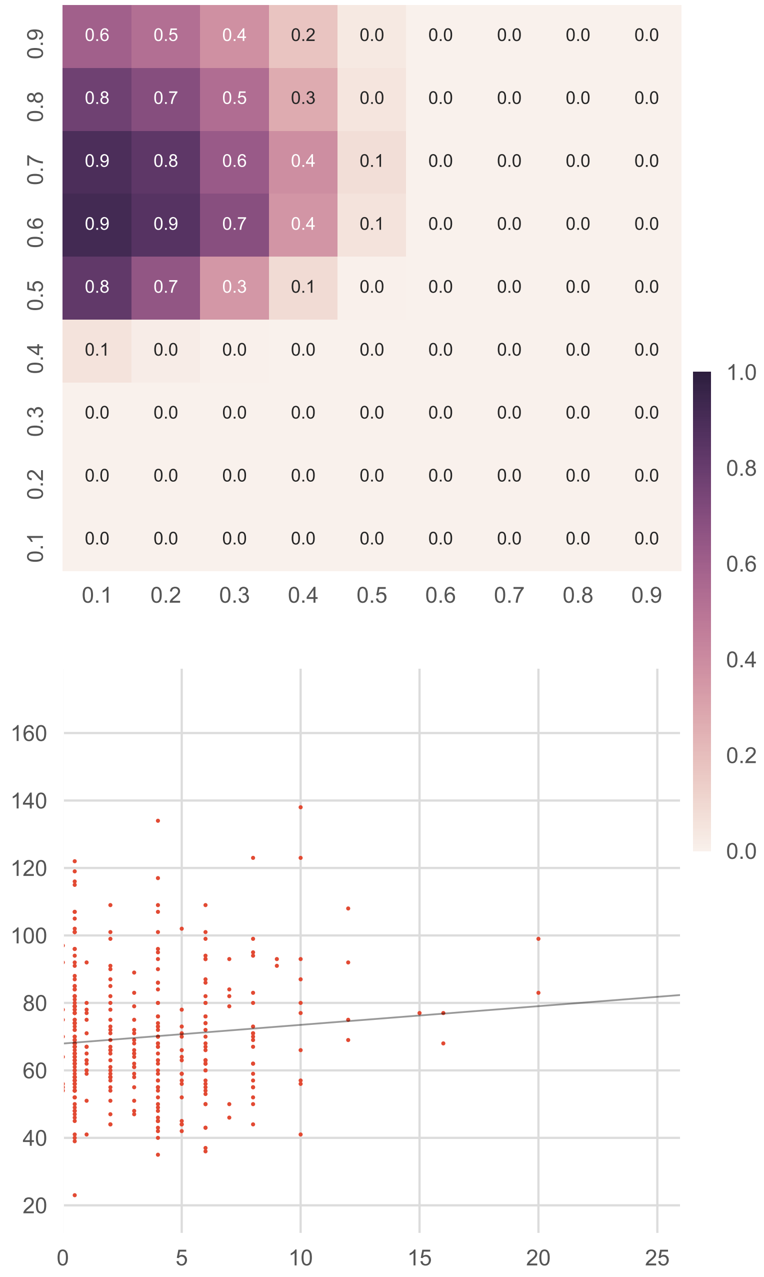

Figure 29: The fitted line was y = 67.955 + 0.554x. The Pearson Correlation Coefficient for the dataset was 0.101 with a p-value of 0.061. The Spearman Rank Correlation Coefficient for the dataset was 0.024. The Kendall Tau Rank Correlation Coefficient for the dataset was 0.015). The normalized mutual information content was 0.273. Causal Direction for this dataset was incorrectly predicted.

30 Dataset-30

x: drinks number of half-pint equivalents of alcoholic beverages
y: sgpt alanine aminotransferase
ground truth:
x-->y

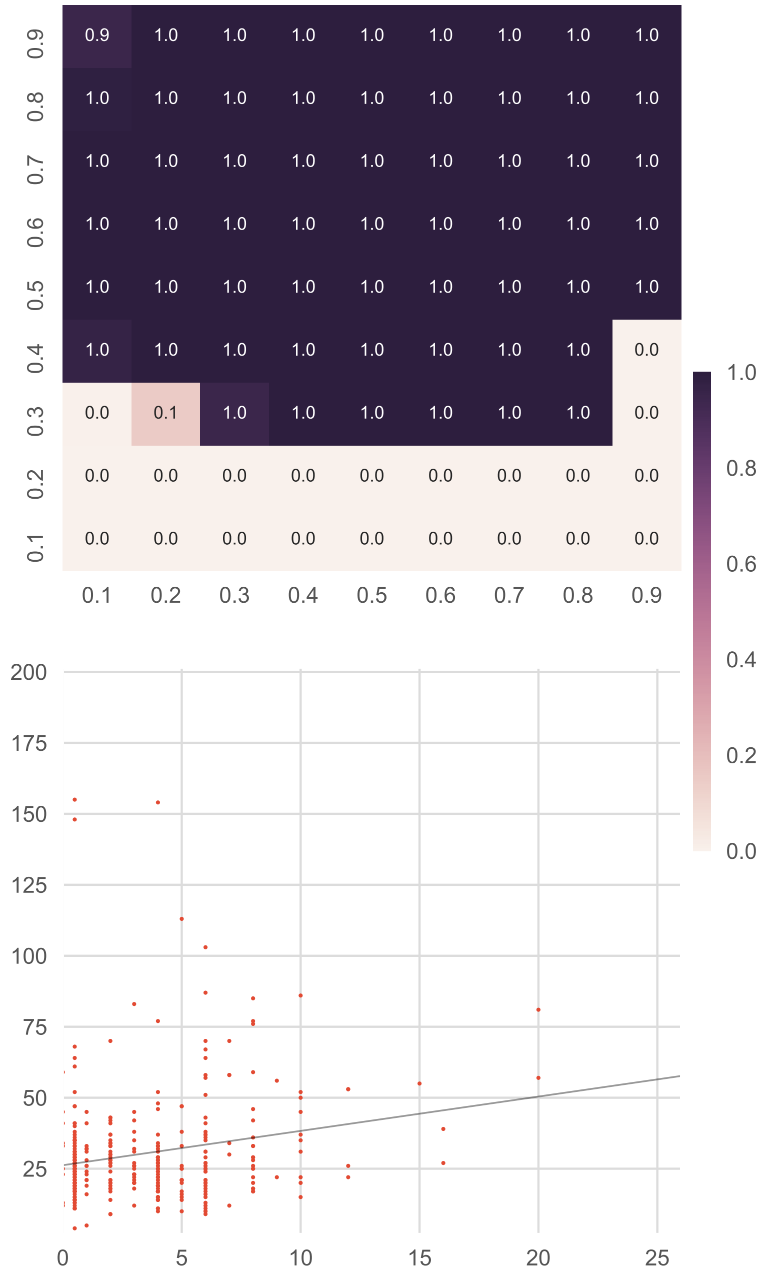

Figure 30: The fitted line was y = 26.228 + 1.209x. The Pearson Correlation Coefficient for the dataset was 0.207 with a p-value of 0.000. The Spearman Rank Correlation Coefficient for the dataset was 0.151. The Kendall Tau Rank Correlation Coefficient for the dataset was 0.107). The normalized mutual information content was 0.244. Causal Direction for this dataset was correctly predicted.

31 Dataset-31

x: drinks number of half-pint equivalents of alcoholic beverages
y: sgot aspartate aminotransferase
ground truth:
x-->y

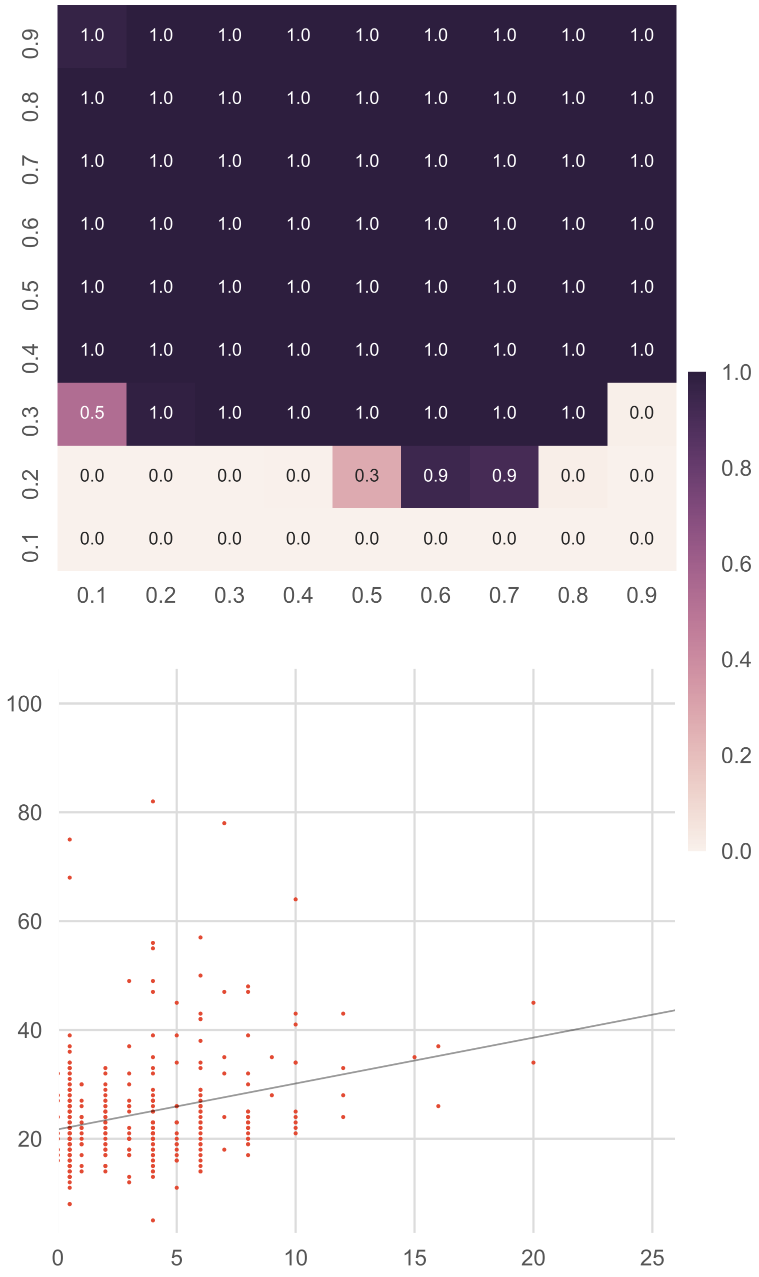

Figure 31: The fitted line was y = 21.731 + 0.843x. The Pearson Correlation Coefficient for the dataset was 0.280 with a p-value of 0.000. The Spearman Rank Correlation Coefficient for the dataset was 0.255. The Kendall Tau Rank Correlation Coefficient for the dataset was 0.185). The normalized mutual information content was 0.213. Causal Direction for this dataset was correctly predicted.

32 Dataset-32

x: drinks number of half-pint equivalents of alcoholic beverages
y: gammagt gamma-glutamyl transpeptdase
ground truth:
x-->y

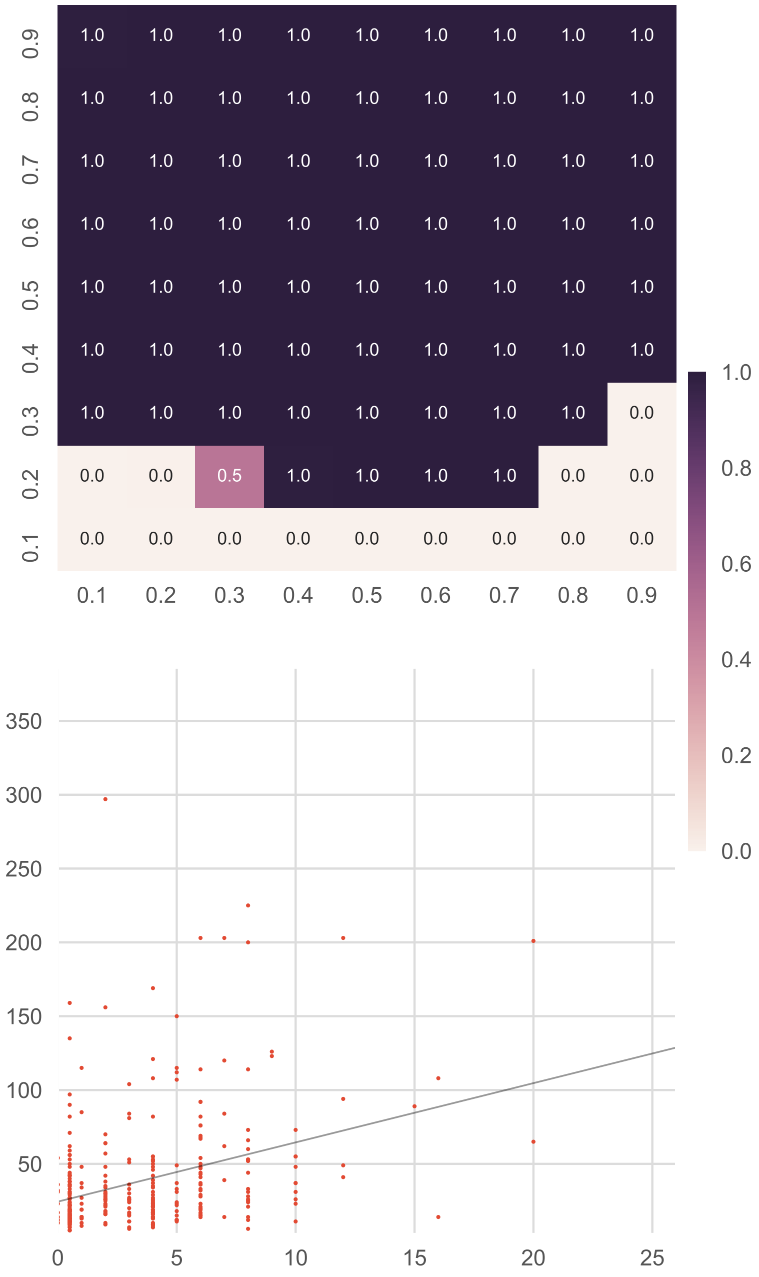

Figure 32: The fitted line was y = 24.419 + 4.013x. The Pearson Correlation Coefficient for the dataset was 0.341 with a p-value of 0.000. The Spearman Rank Correlation Coefficient for the dataset was 0.342. The Kendall Tau Rank Correlation Coefficient for the dataset was 0.249). The normalized mutual information content was 0.243. Causal Direction for this dataset was correctly predicted.

33 Dataset-33

x: age
y: body mass index (weight in kg/(height in m)^2)
ground truth:
x --> y

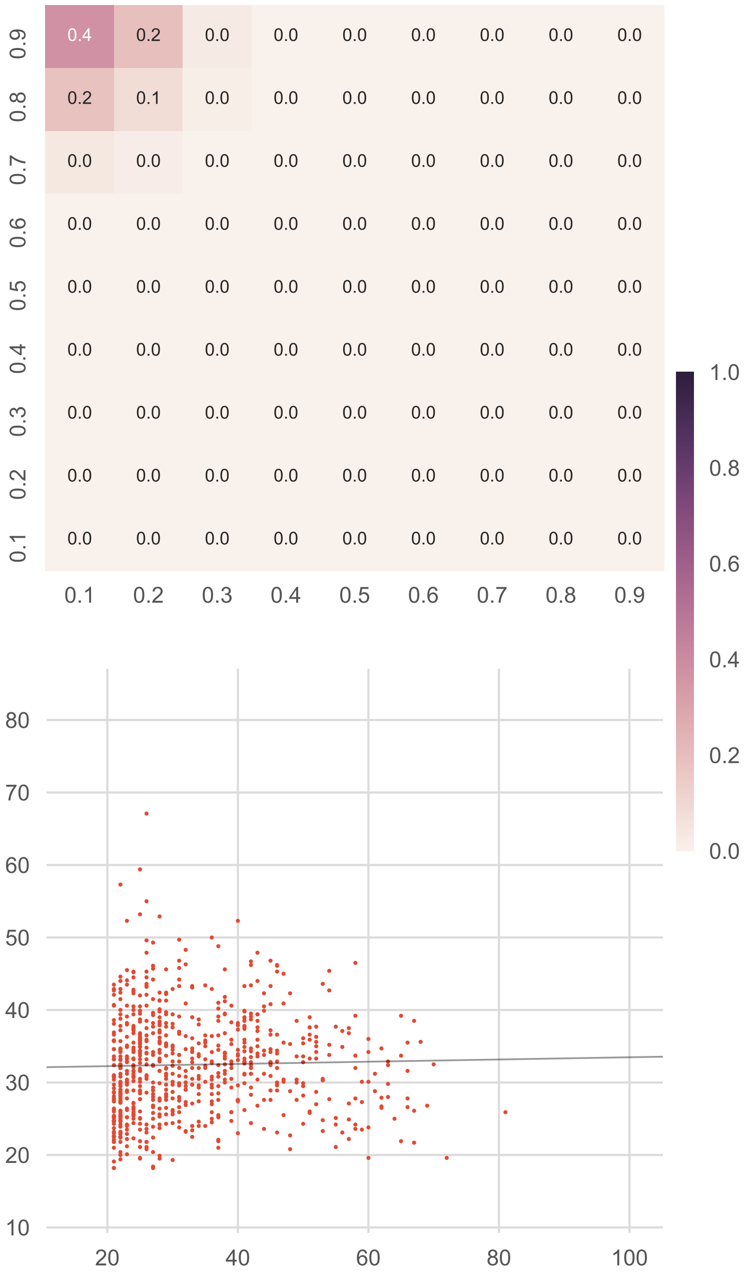

Figure 33: The fitted line was y = 31.948 + 0.015x. The Pearson Correlation Coefficient for the dataset was 0.026 with a p-value of 0.478. The Spearman Rank Correlation Coefficient for the dataset was 0.121. The Kendall Tau Rank Correlation Coefficient for the dataset was 0.082). The normalized mutual information content was 0.318. Causal Direction for this dataset could not be predicted.

34 Dataset-34

x: age
y: diastolic blood pressure (mm Hg)
ground truth:
x --> y

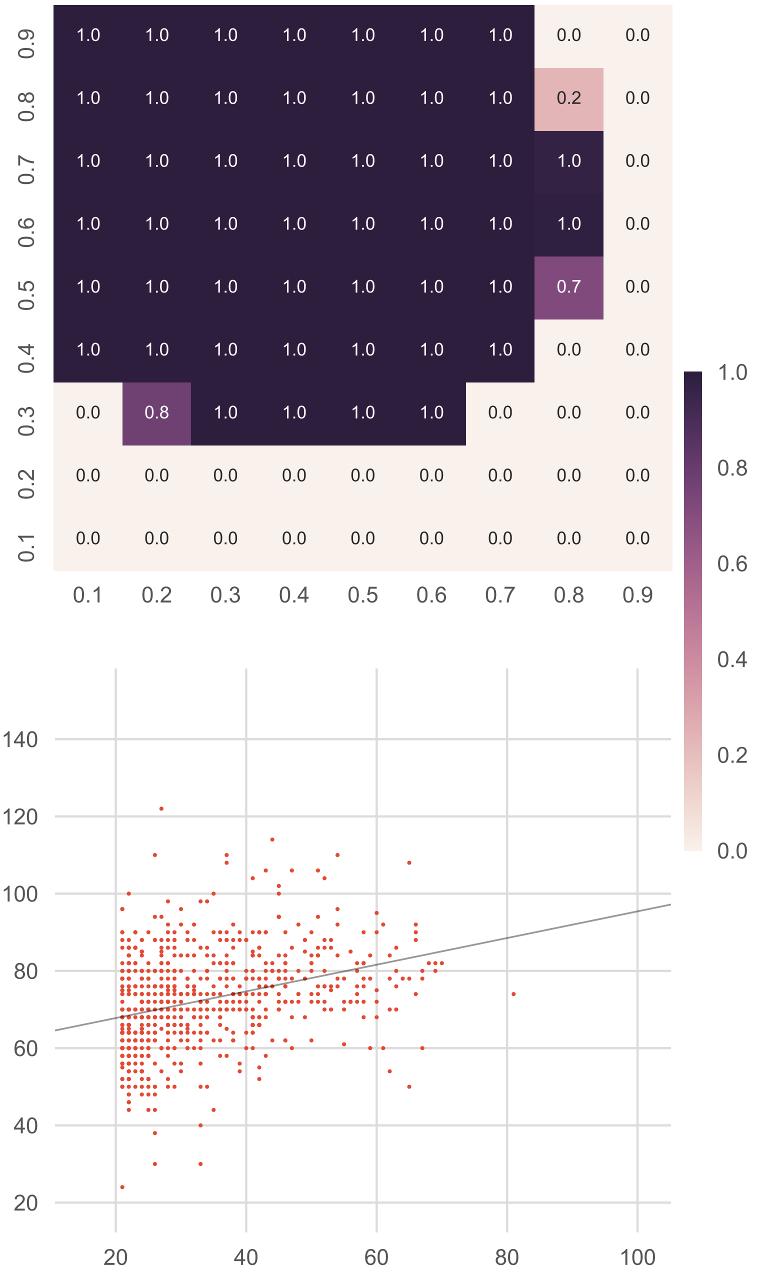

Figure 34: The fitted line was y = 60.885 + 0.345x. The Pearson Correlation Coefficient for the dataset was 0.330 with a p-value of 0.000. The Spearman Rank Correlation Coefficient for the dataset was 0.371. The Kendall Tau Rank Correlation Coefficient for the dataset was 0.262). The normalized mutual information content was 0.252. Causal Direction for this dataset was correctly predicted.

35 Dataset-35

x: age
y: Plasma glucose concentration a 2 hours in an oral glucose tolerance test
ground truth:
x --> y

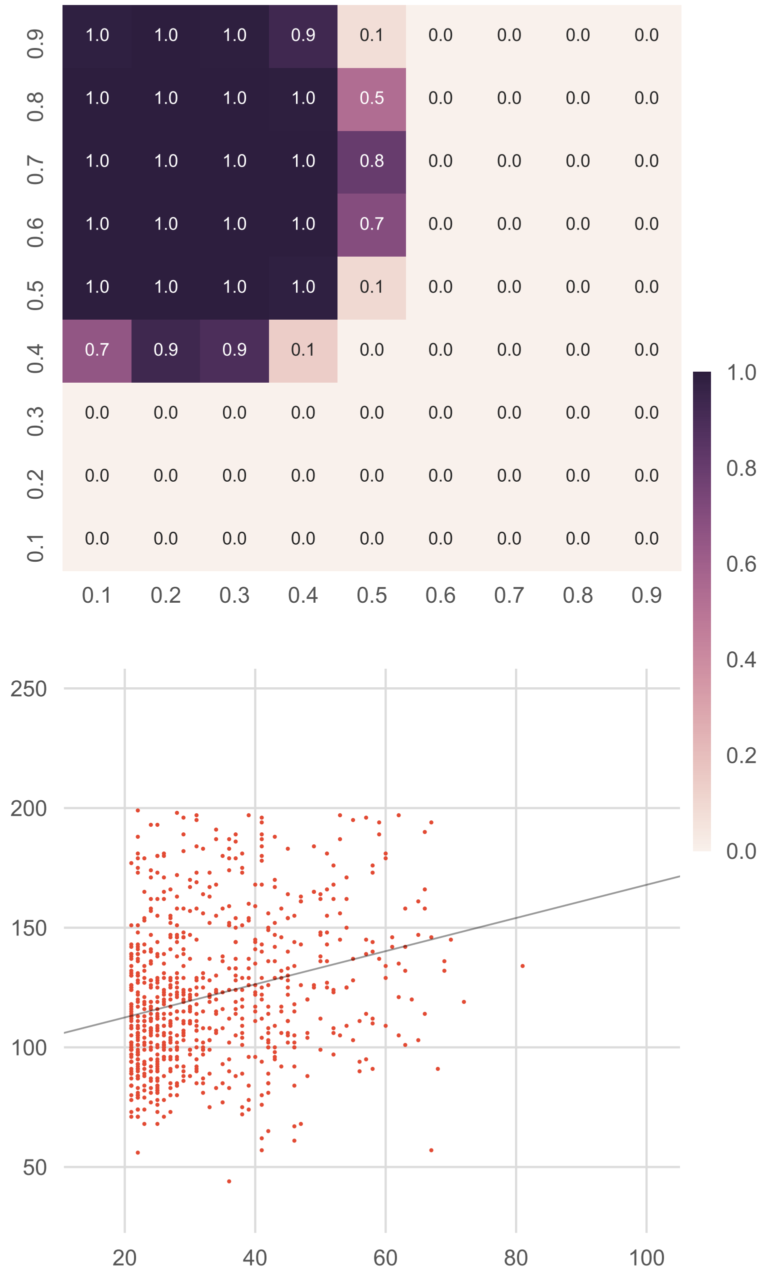

Figure 35: The fitted line was y = 98.632 + 0.693x. The Pearson Correlation Coefficient for the dataset was 0.267 with a p-value of 0.000. The Spearman Rank Correlation Coefficient for the dataset was 0.283. The Kendall Tau Rank Correlation Coefficient for the dataset was 0.195). The normalized mutual information content was 0.355. Causal Direction for this dataset was incorrectly predicted.

36 Dataset-36

x: number of cars per 24h at different counting stations in Oberschwaben, Germany
y: categorical :
ground truth:
y --> x

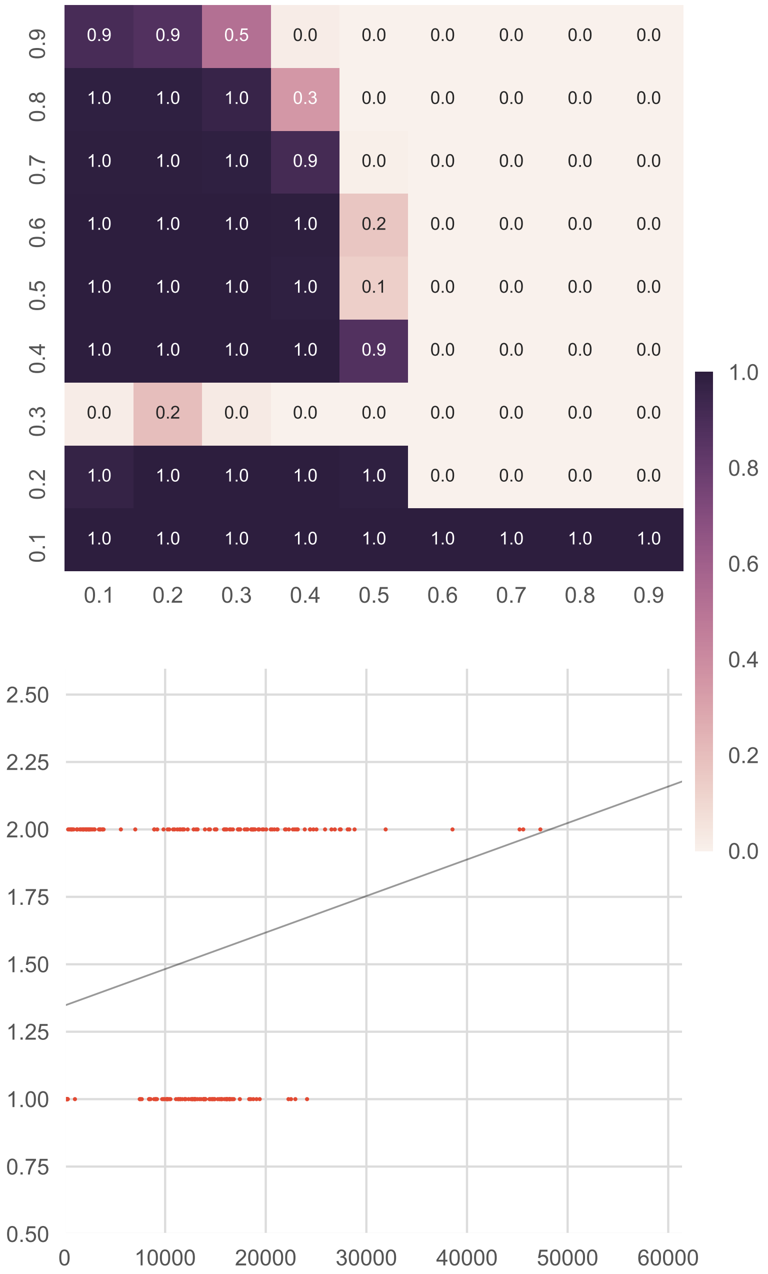

Figure 36: The fitted line was y = 1.347 + 0.000x. The Pearson Correlation Coefficient for the dataset was 0.247 with a p-value of 0.000. The Spearman Rank Correlation Coefficient for the dataset was 0.299. The Kendall Tau Rank Correlation Coefficient for the dataset was 0.245). The normalized mutual information content was 0.231. Causal Direction for this dataset was correctly predicted.

37 Dataset-37

x: Indoor temperature
y: Outdoor temperature
ground truth:
y-->x

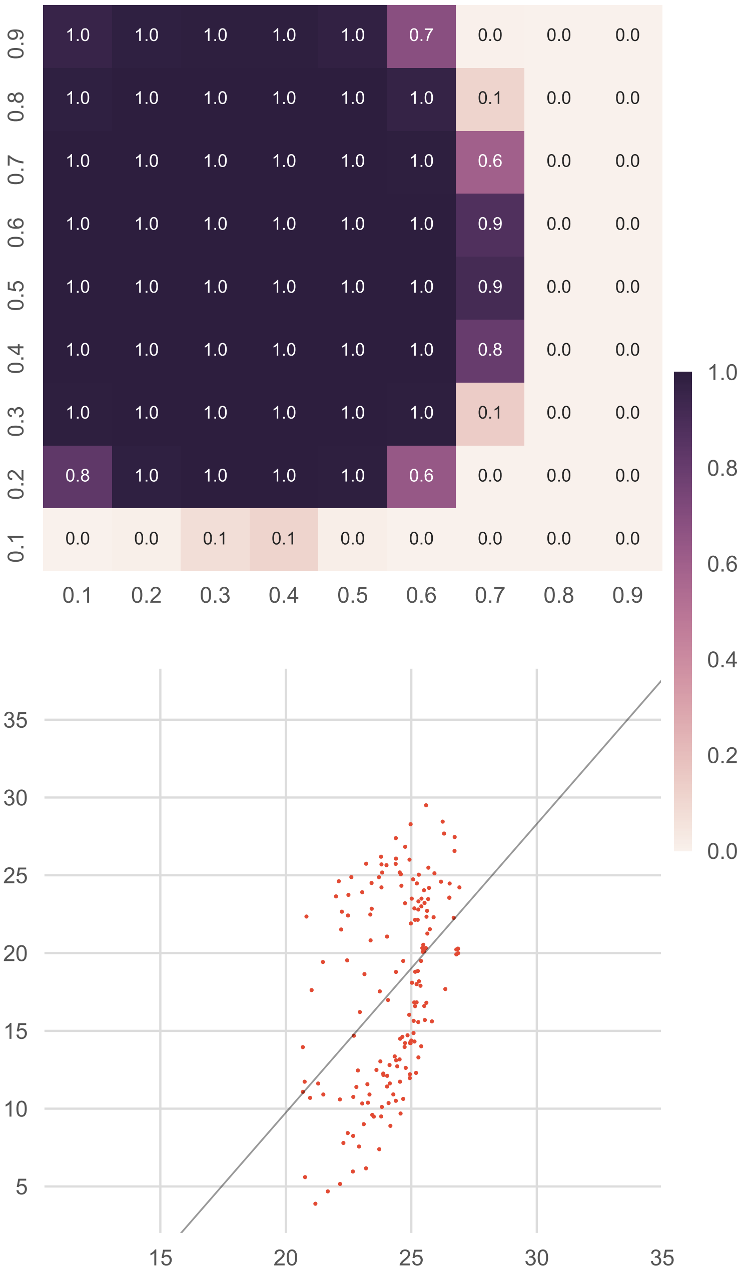

Figure 37: The fitted line was y = -27.357 + 1.855x. The Pearson Correlation Coefficient for the dataset was 0.444 with a p-value of 0.000. The Spearman Rank Correlation Coefficient for the dataset was 0.436. The Kendall Tau Rank Correlation Coefficient for the dataset was 0.327). The normalized mutual information content was 0.770. Causal Direction for this dataset was correctly predicted.

38 Dataset-38

x: Ozone (microgram / cubic meter)
y: Temperature (degree celsius)
ground truth:
y-->x

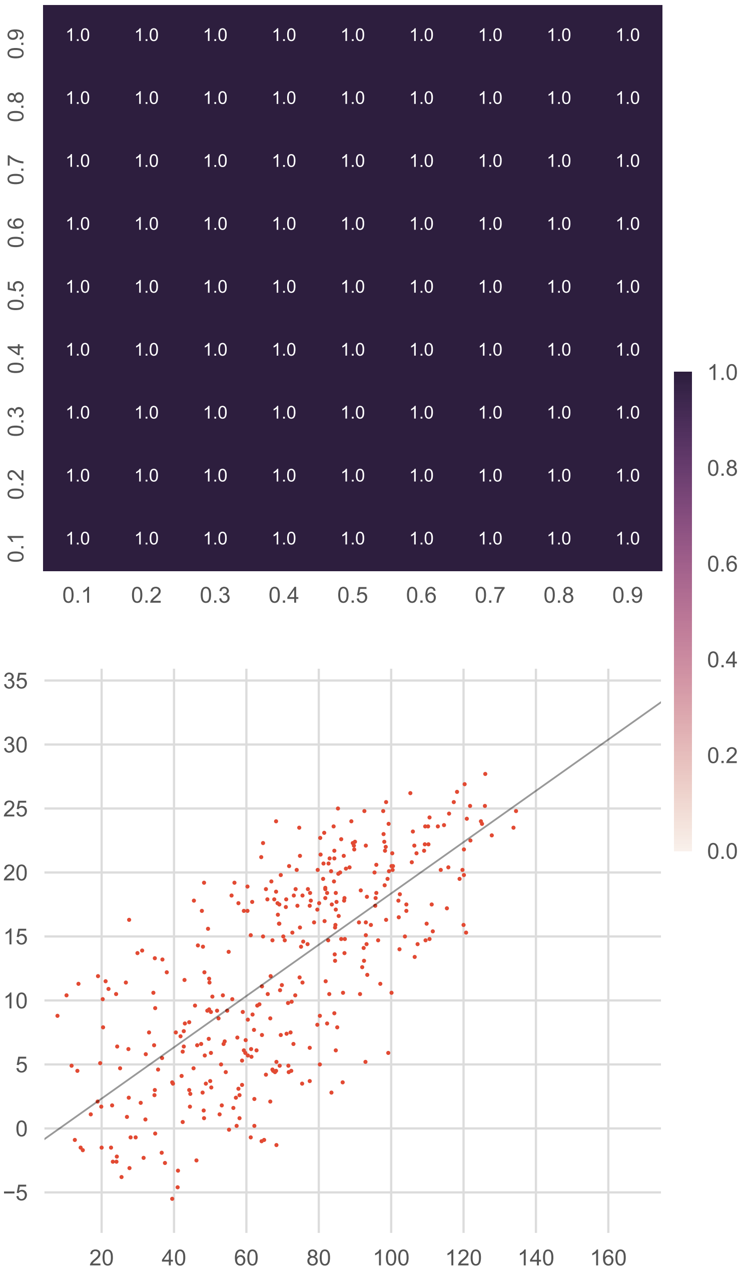

Figure 38: The fitted line was y = -1.677 + 0.200x. The Pearson Correlation Coefficient for the dataset was 0.713 with a p-value of 0.000. The Spearman Rank Correlation Coefficient for the dataset was 0.720. The Kendall Tau Rank Correlation Coefficient for the dataset was 0.516). The normalized mutual information content was 0.651. Causal Direction for this dataset could not be predicted.

39 Dataset-39

x: Ozone (microgram / cubic meter)
y: Temperature (degree celsius)
ground truth:
y-->x

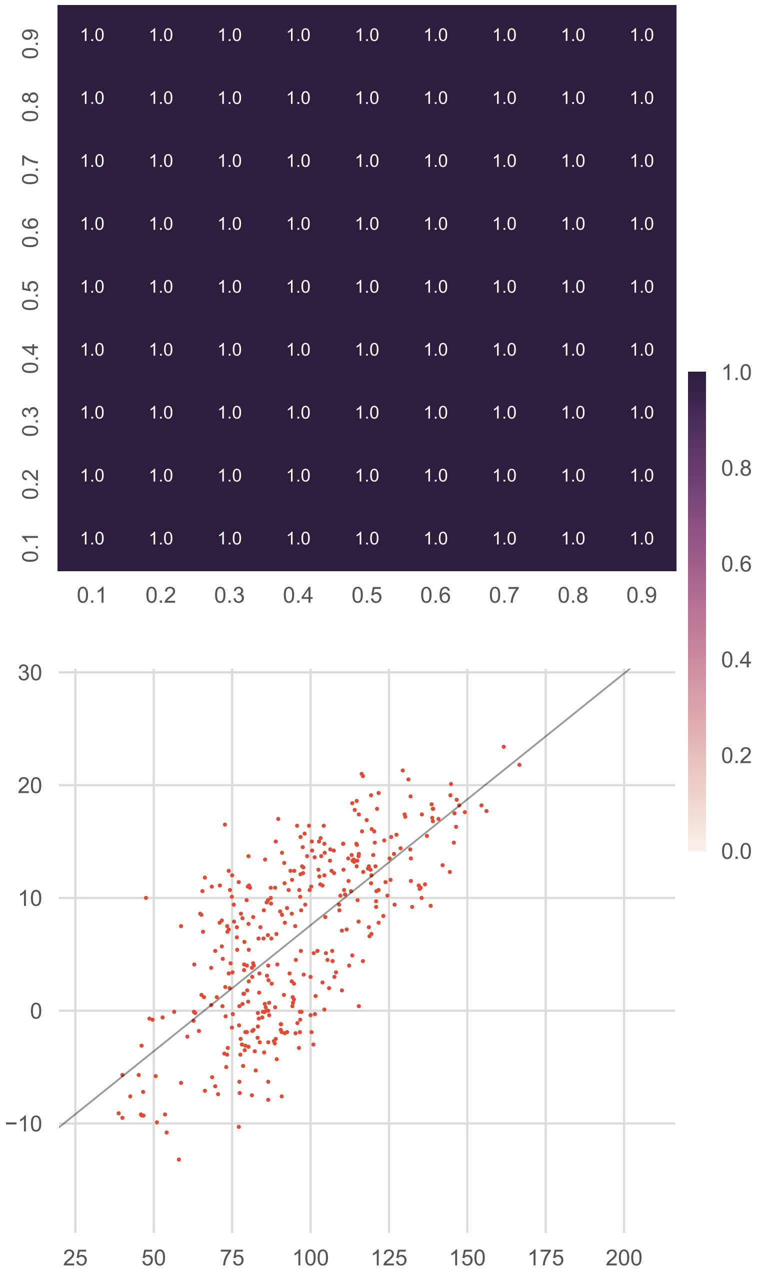

Figure 39: The fitted line was y = -14.761 + 0.223x. The Pearson Correlation Coefficient for the dataset was 0.703 with a p-value of 0.000. The Spearman Rank Correlation Coefficient for the dataset was 0.683. The Kendall Tau Rank Correlation Coefficient for the dataset was 0.489). The normalized mutual information content was 0.621. Causal Direction for this dataset could not be predicted.

40 Dataset-40

x: Ozone (microgram / cubic meter)
y: Temperature (degree celsius)
ground truth:
y-->x

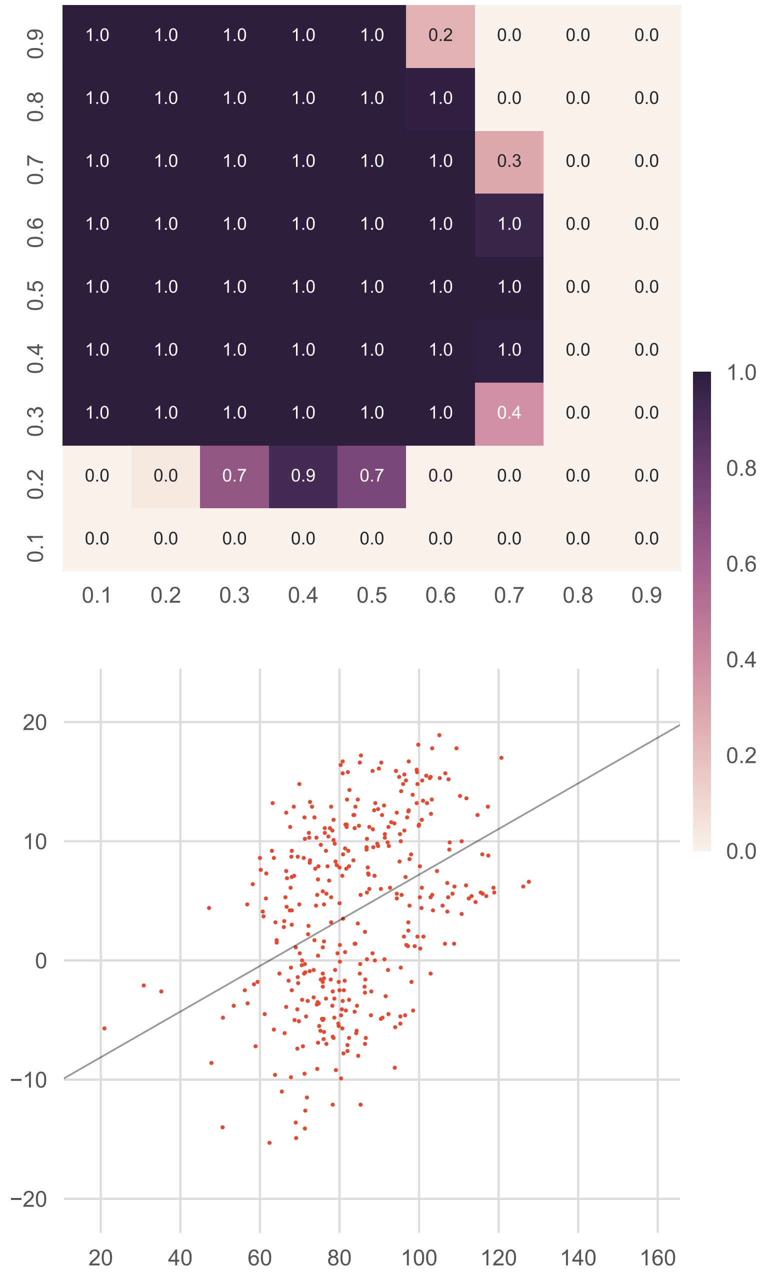

Figure 40: The fitted line was y = -11.951 + 0.191x. The Pearson Correlation Coefficient for the dataset was 0.393 with a p-value of 0.000. The Spearman Rank Correlation Coefficient for the dataset was 0.390. The Kendall Tau Rank Correlation Coefficient for the dataset was 0.260). The normalized mutual information content was 0.588. Causal Direction for this dataset was correctly predicted.

41 Dataset-41

x: life expectancy at birth for different countries, female, 2000-2005
y: latitude of the country's capital
ground truth:
y-->x

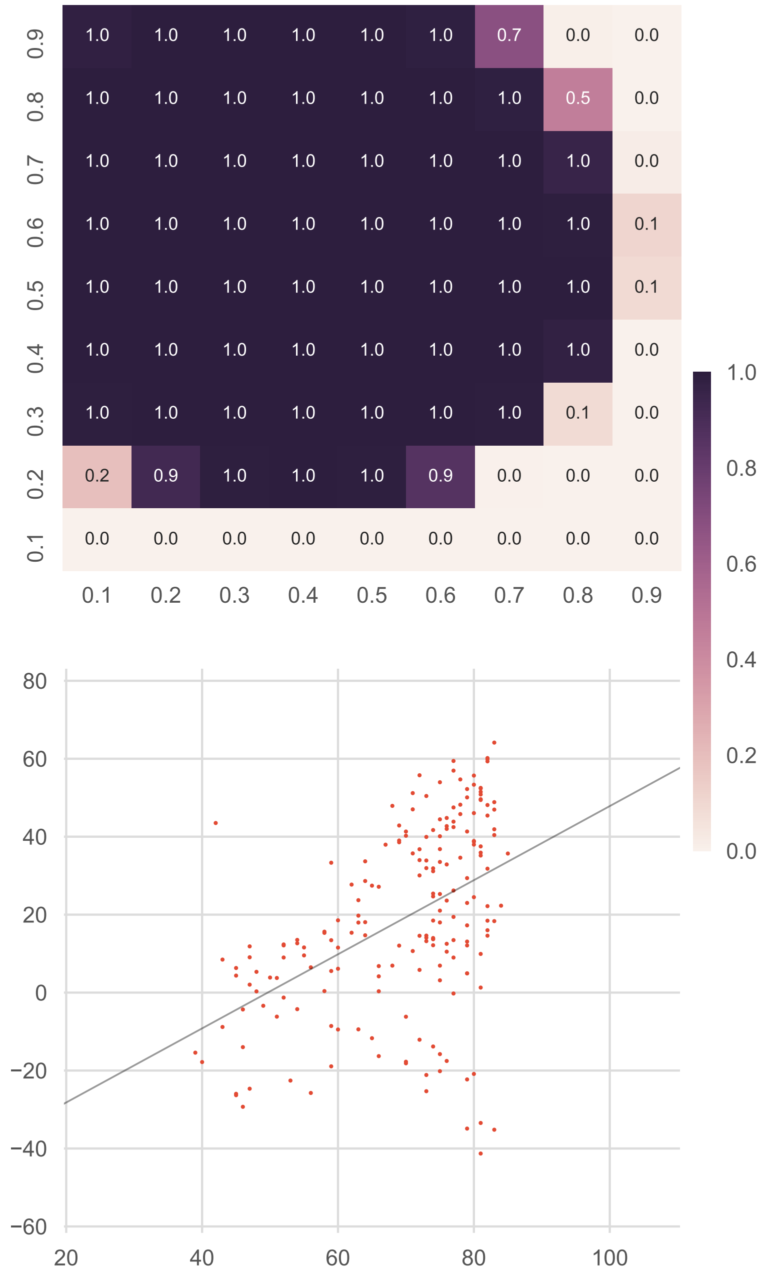

Figure 41: The fitted line was y = -47.202 + 0.951x. The Pearson Correlation Coefficient for the dataset was 0.462 with a p-value of 0.000. The Spearman Rank Correlation Coefficient for the dataset was 0.487. The Kendall Tau Rank Correlation Coefficient for the dataset was 0.352). The normalized mutual information content was 0.661. Causal Direction for this dataset was correctly predicted.

42 Dataset-42

x: life expectancy at birth for different countries, female, 1995-2000
y: latitude of the country's capital
ground truth:
y-->x

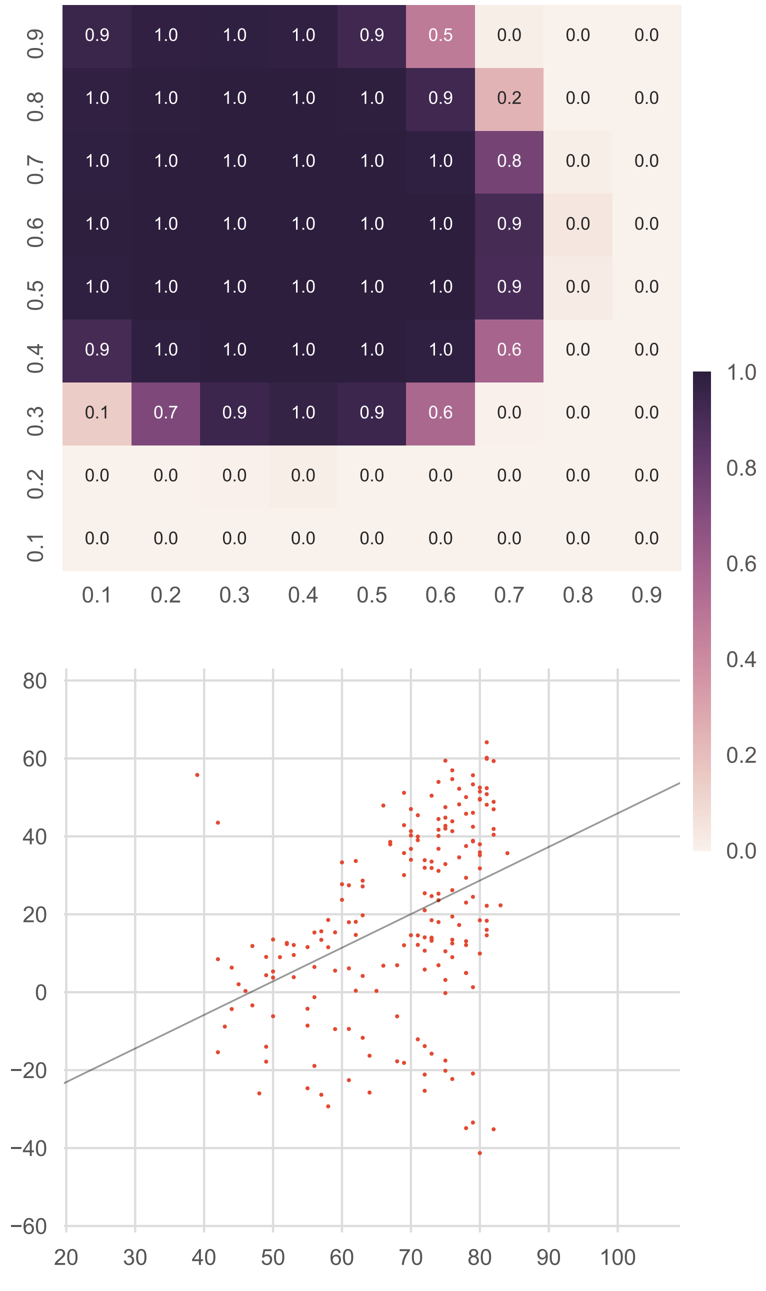

Figure 42: The fitted line was y = -40.313 + 0.862x. The Pearson Correlation Coefficient for the dataset was 0.397 with a p-value of 0.000. The Spearman Rank Correlation Coefficient for the dataset was 0.458. The Kendall Tau Rank Correlation Coefficient for the dataset was 0.326). The normalized mutual information content was 0.654. Causal Direction for this dataset was correctly predicted.

43 Dataset-43

x: life expectancy at birth for different countries, female, 1990-1995
y: latitude of the country's capital
ground truth:
y-->x

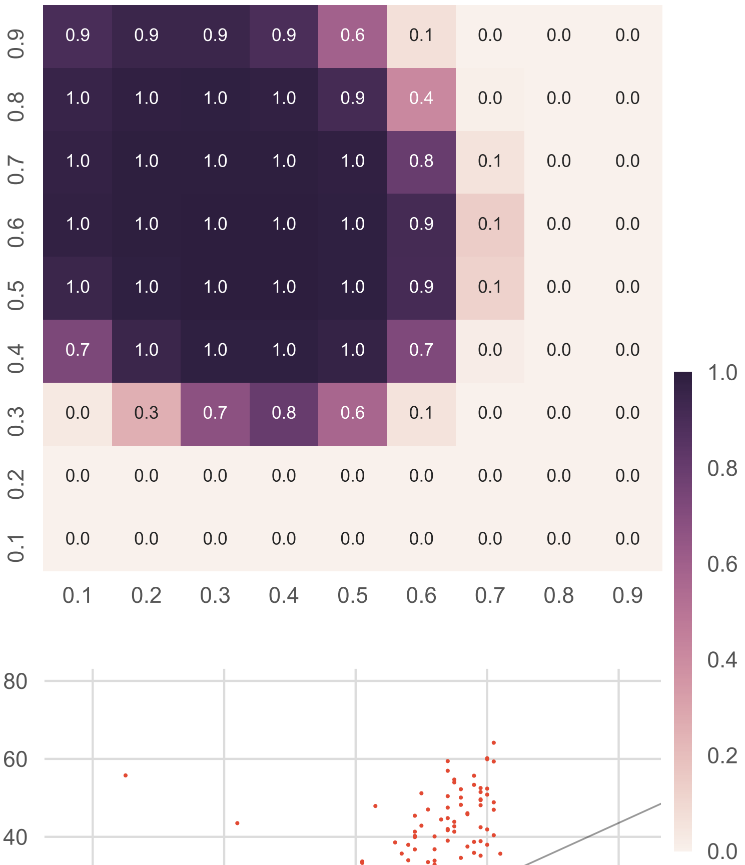

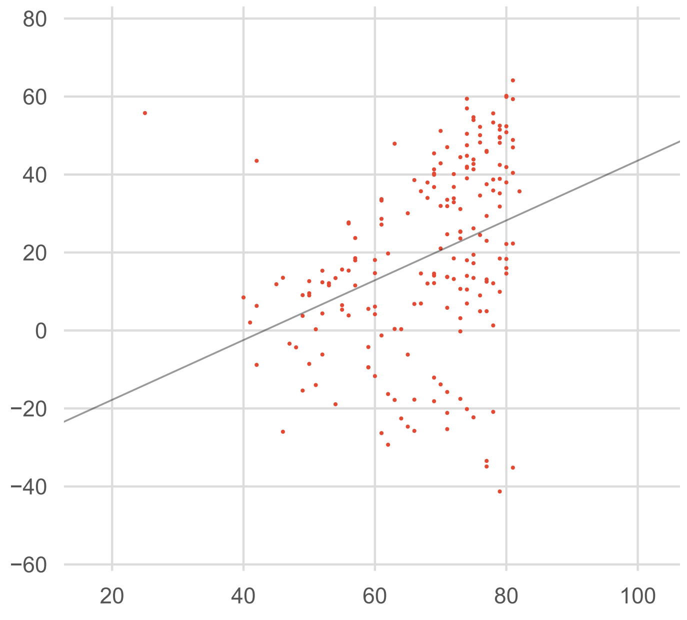

Figure 43: The fitted line was y = -33.128 + 0.767x. The Pearson Correlation Coefficient for the dataset was 0.349 with a p-value of 0.000. The Spearman Rank Correlation Coefficient for the dataset was 0.450. The Kendall Tau Rank Correlation Coefficient for the dataset was 0.315). The normalized mutual information content was 0.656. Causal Direction for this dataset was correctly predicted.

44 Dataset-44

x: life expectancy at birth for different countries, female, 1985-1990
y: latitude of the country's capital
ground truth:
y-->x

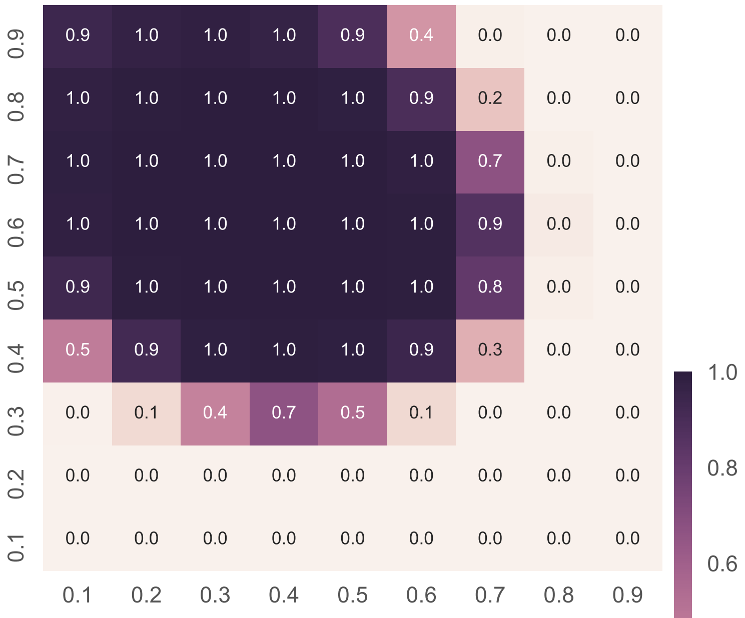

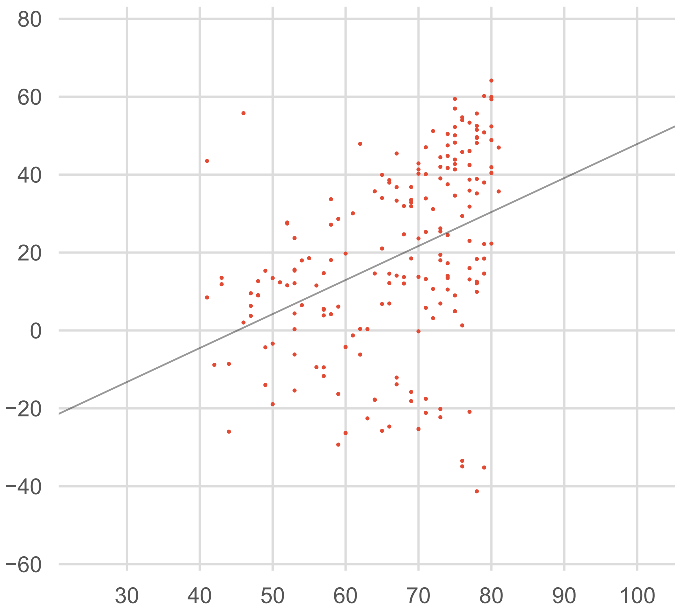

Figure 44: The fitted line was y = -39.436 + 0.873x. The Pearson Correlation Coefficient for the dataset was 0.387 with a p-value of 0.000. The Spearman Rank Correlation Coefficient for the dataset was 0.468. The Kendall Tau Rank Correlation Coefficient for the dataset was 0.327). The normalized mutual information content was 0.656. Causal Direction for this dataset was incorrectly predicted.

45 Dataset-45

x: life expectancy at birth for different countries, male, 2000-2005
y: latitude of the country's capital
ground truth:
y-->x

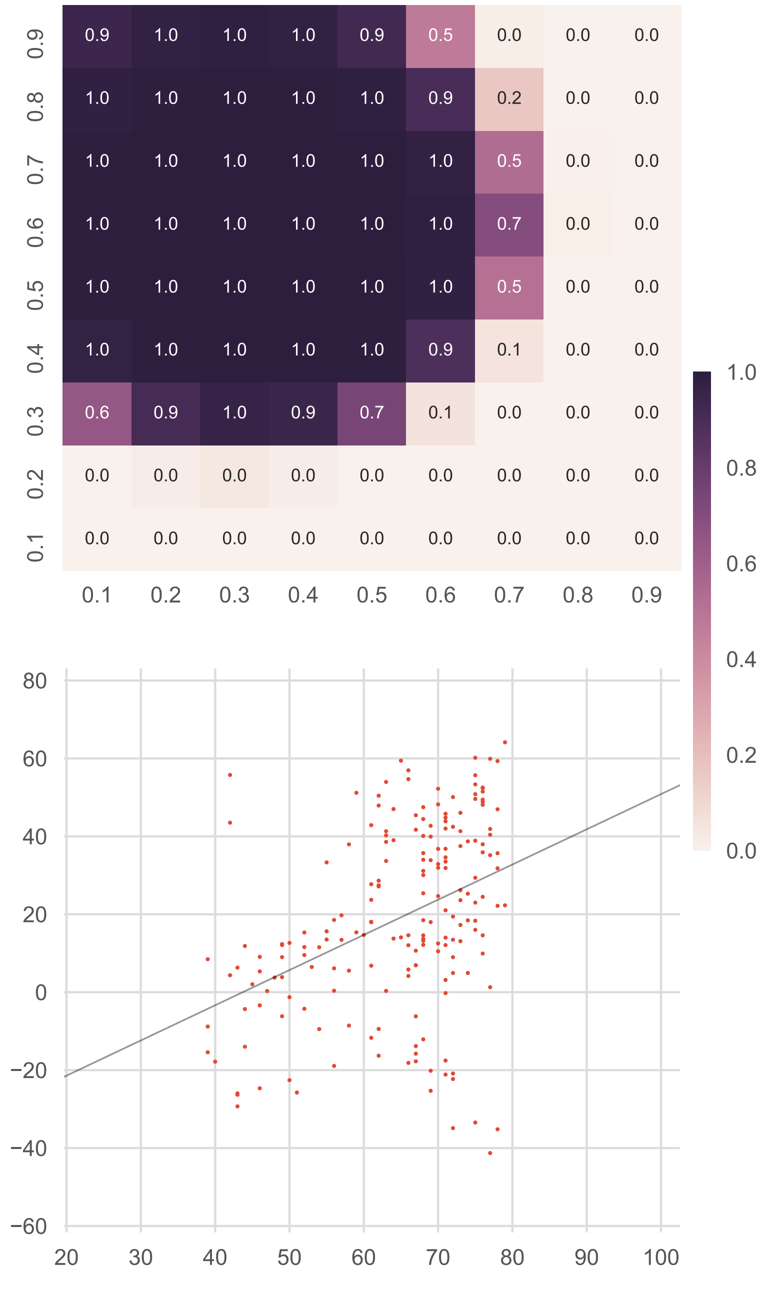

Figure 45: The fitted line was y = -39.474 + 0.903x. The Pearson Correlation Coefficient for the dataset was 0.395 with a p-value of 0.000. The Spearman Rank Correlation Coefficient for the dataset was 0.408. The Kendall Tau Rank Correlation Coefficient for the dataset was 0.295). The normalized mutual information content was 0.641. Causal Direction for this dataset was correctly predicted.

46 Dataset-46

x: life expectancy at birth for different countries, male, 1995-2000
y: latitude of the country's capital
ground truth:
y-->x

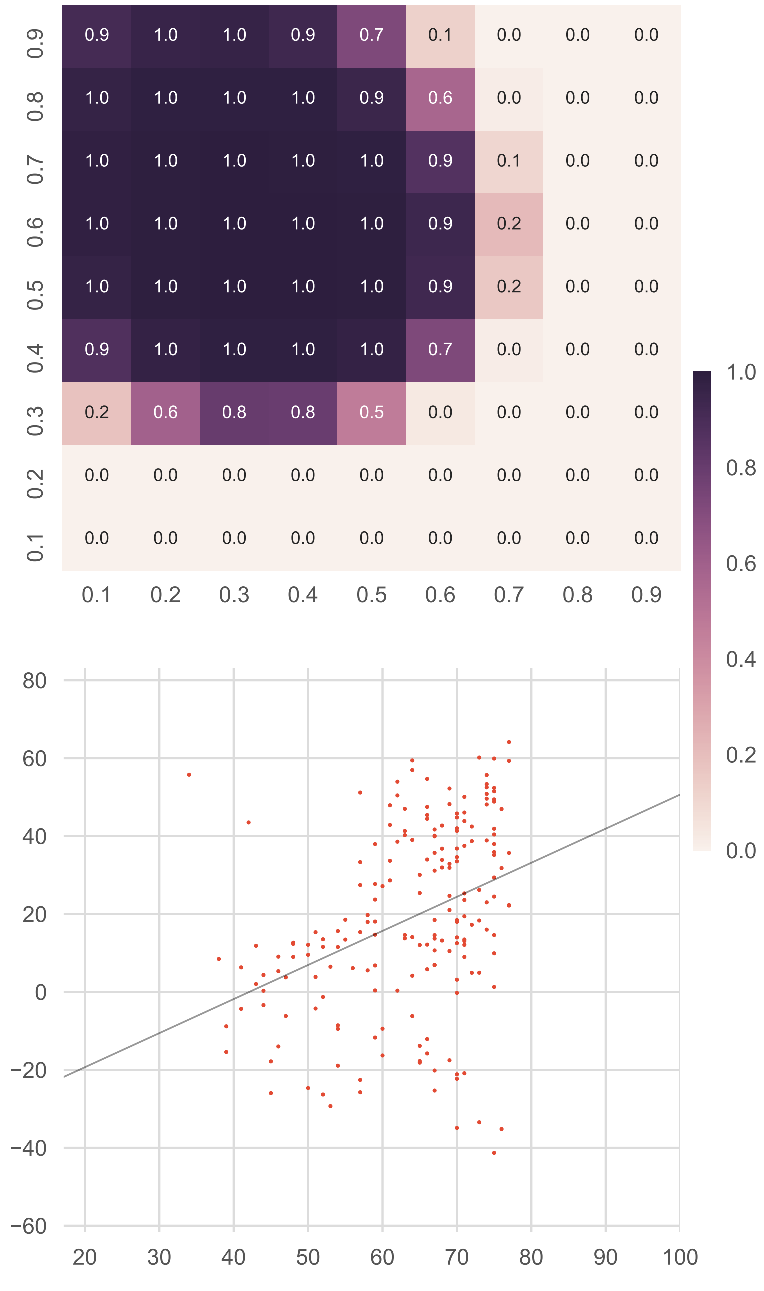

Figure 46: The fitted line was y = -36.809 + 0.875x. The Pearson Correlation Coefficient for the dataset was 0.364 with a p-value of 0.000. The Spearman Rank Correlation Coefficient for the dataset was 0.404. The Kendall Tau Rank Correlation Coefficient for the dataset was 0.285). The normalized mutual information content was 0.635. Causal Direction for this dataset was correctly predicted.

47 Dataset-47

x: life expectancy at birth for different countries, male, 1990-1995
y: latitude of the country's capital
ground truth:
y-->x

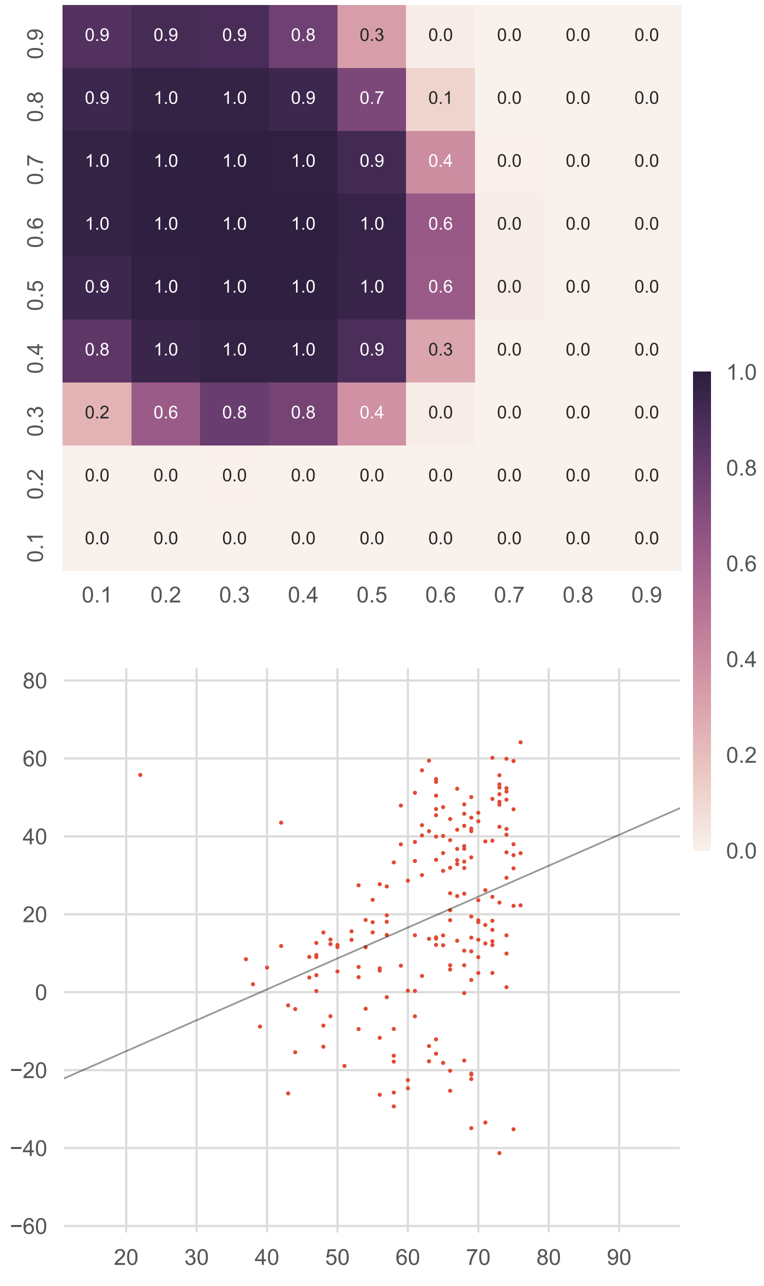

Figure 47: The fitted line was y = -30.992 + 0.793x. The Pearson Correlation Coefficient for the dataset was 0.322 with a p-value of 0.000. The Spearman Rank Correlation Coefficient for the dataset was 0.392. The Kendall Tau Rank Correlation Coefficient for the dataset was 0.269). The normalized mutual information content was 0.640. Causal Direction for this dataset was correctly predicted.

48 Dataset-48

x: life expectancy at birth for different countries, male, 1985-1990
y: latitude of the country's capital
ground truth:
y-->x

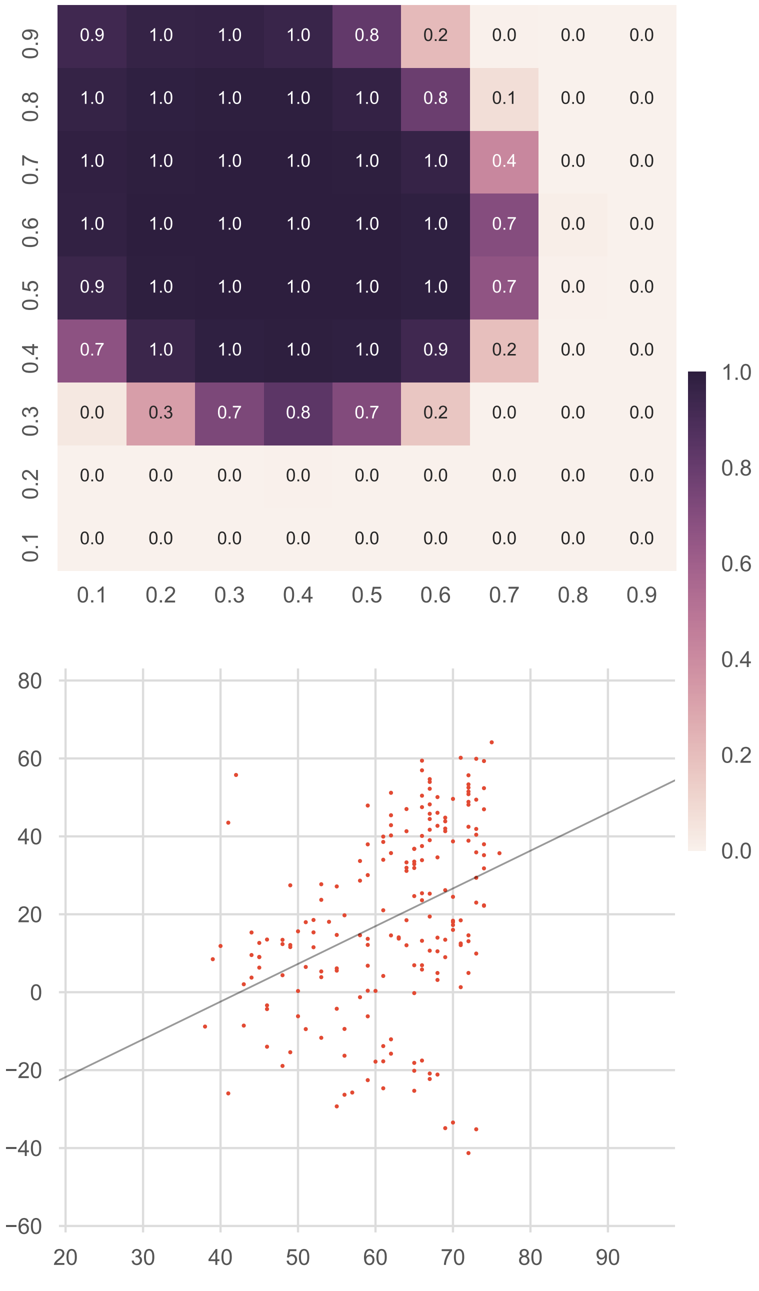

Figure 48: The fitted line was y = -41.153 + 0.968x. The Pearson Correlation Coefficient for the dataset was 0.375 with a p-value of 0.000. The Spearman Rank Correlation Coefficient for the dataset was 0.433. The Kendall Tau Rank Correlation Coefficient for the dataset was 0.298). The normalized mutual information content was 0.636. Causal Direction for this dataset was correctly predicted.

49 Dataset-49

x: Population with sustainable access to improved drinking water sources (%) total, 2006
y: Infant mortality rate (per 1 000 live births) both sexes, 2006
ground truth:
x-->y

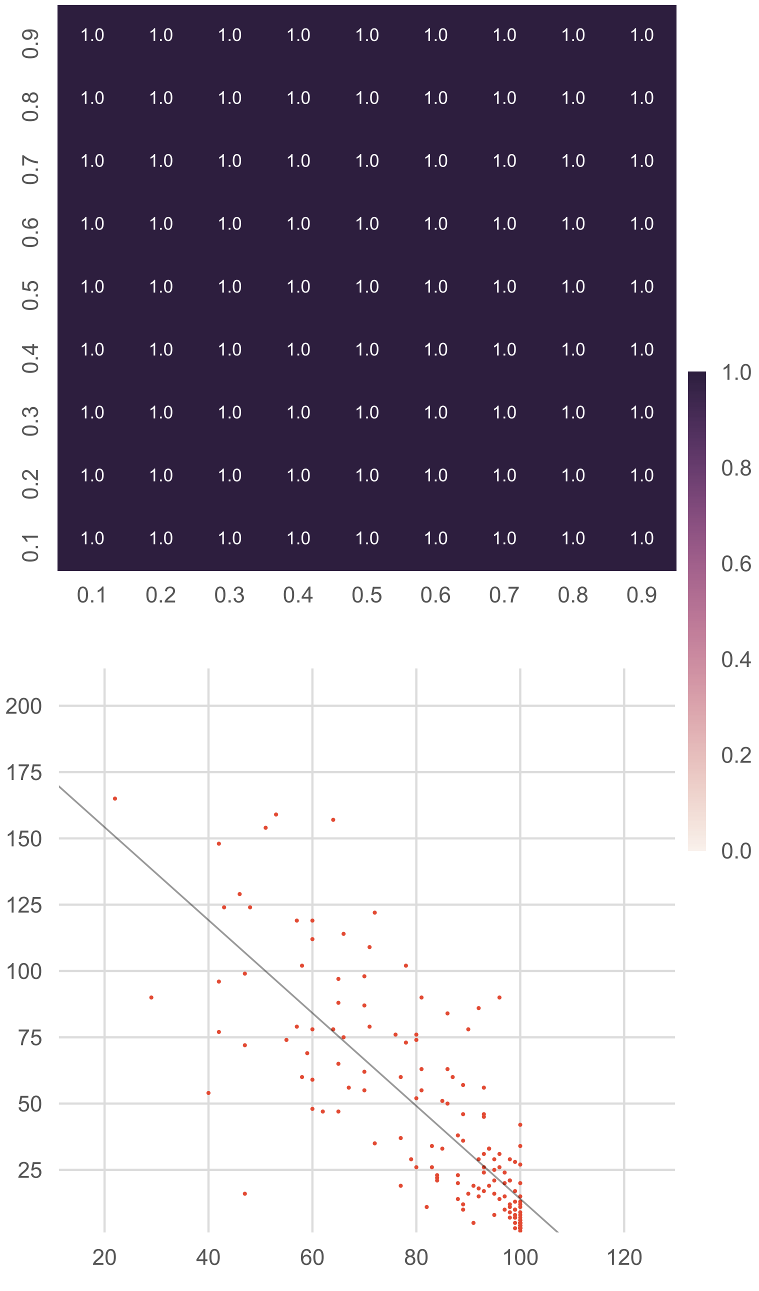

Figure 49: The fitted line was y = 189.254 + -1.752x. The Pearson Correlation Coefficient for the dataset was -0.804 with a p-value of 0.000. The Spearman Rank Correlation Coefficient for the dataset was -0.846. The Kendall Tau Rank Correlation Coefficient for the dataset was -0.676). The normalized mutual information content was 0.707. Causal Direction for this dataset could not be predicted.

50 Dataset-50

x: stock returns of Hang Seng Bank (0011.HK)
y: stock return of HSBC Hldgs (0005.HK)
ground truth:
x-->y

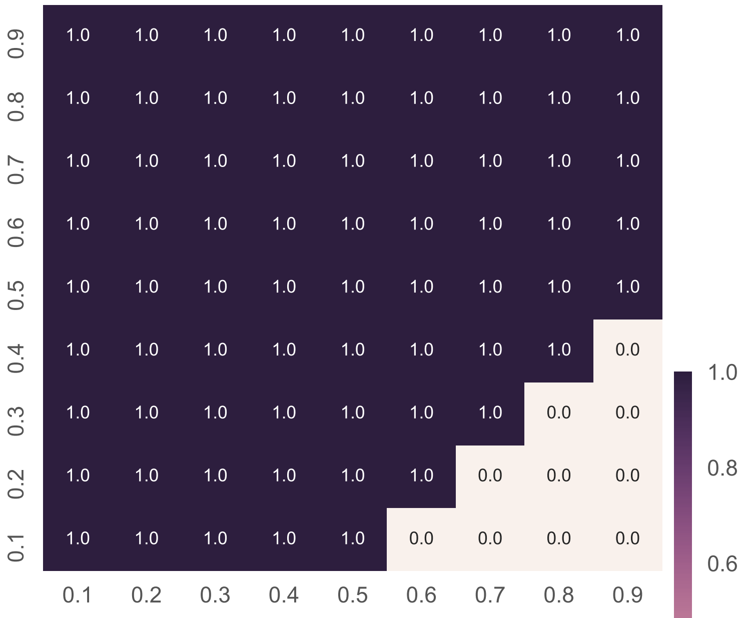

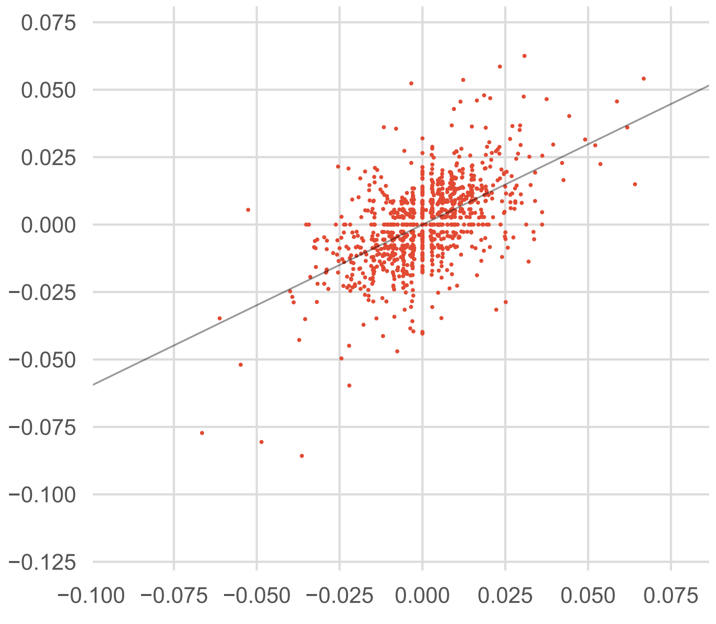

Figure 50: The fitted line was y = -0.000 + 0.597x. The Pearson Correlation Coefficient for the dataset was 0.568 with a p-value of 0.000. The Spearman Rank Correlation Coefficient for the dataset was 0.536. The Kendall Tau Rank Correlation Coefficient for the dataset was 0.393). The normalized mutual information content was 0.265. Causal Direction for this dataset could not be predicted.

51 Dataset-51

x: stock returns of Hutchison (0013.HK)
y: stock return of Cheung kong (0001.HK)
ground truth:
x-->y

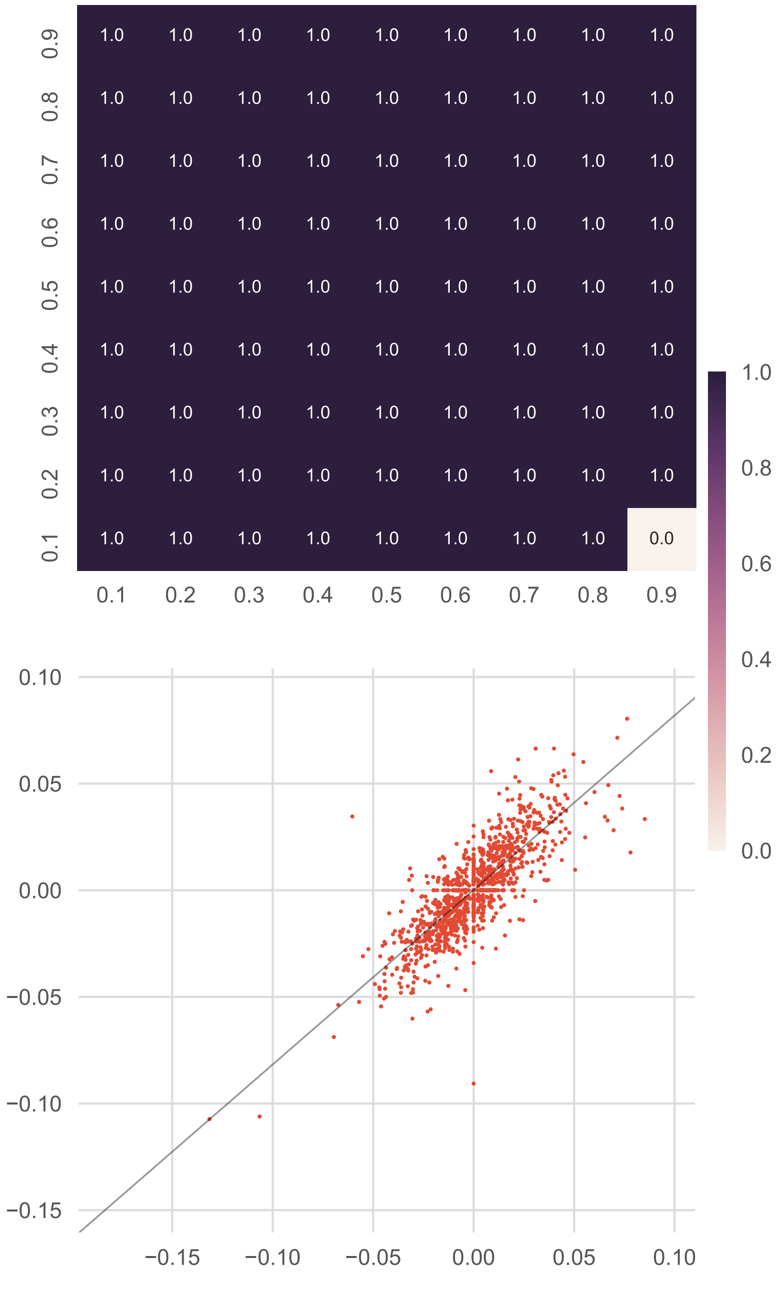

Figure 51: The fitted line was y = 0.000 + 0.818x. The Pearson Correlation Coefficient for the dataset was 0.814 with a p-value of 0.000. The Spearman Rank Correlation Coefficient for the dataset was 0.806. The Kendall Tau Rank Correlation Coefficient for the dataset was 0.627). The normalized mutual information content was 0.319. Causal Direction for this dataset could not be predicted.

52 Dataset-52

x: stock returns of Cheung kong (0001.HK)
y: stock return of Sun Hung Kai Prop. (0016.HK)
ground truth:
x-->y

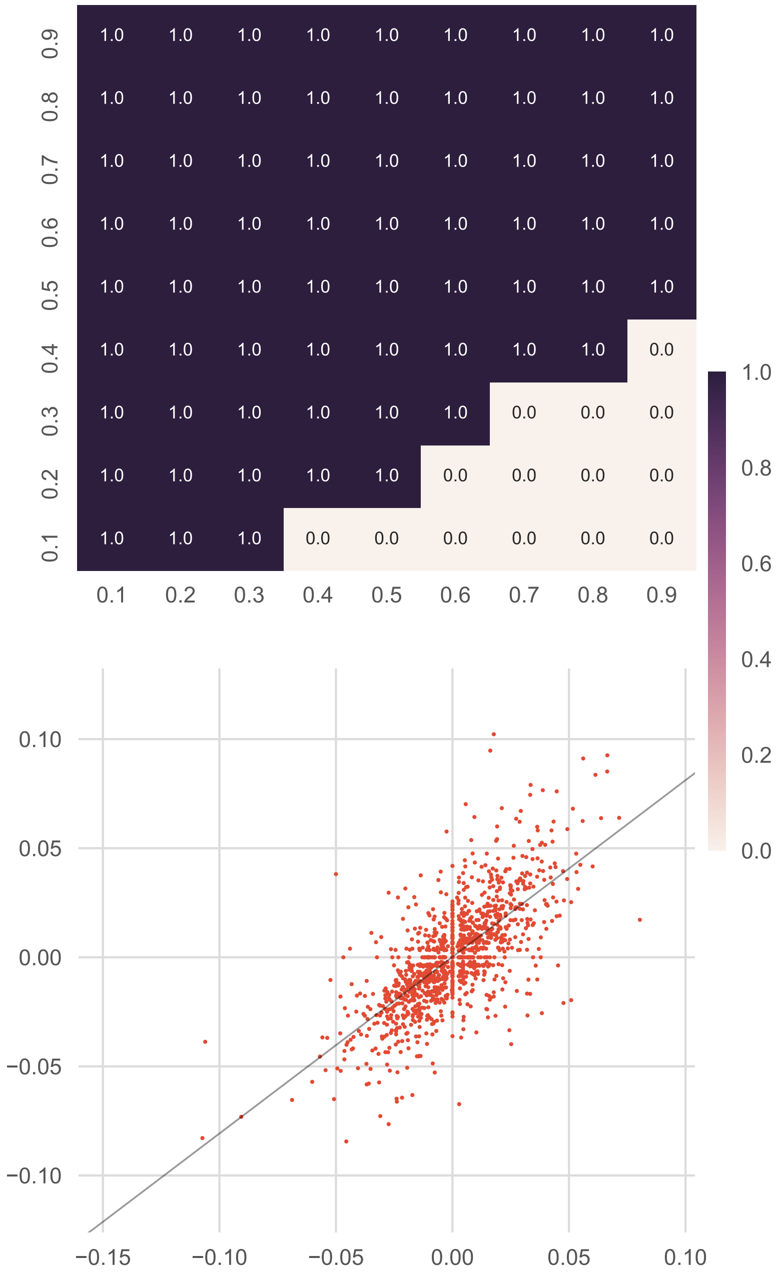

Figure 52: The fitted line was y = 0.000 + 0.810x. The Pearson Correlation Coefficient for the dataset was 0.703 with a p-value of 0.000. The Spearman Rank Correlation Coefficient for the dataset was 0.693. The Kendall Tau Rank Correlation Coefficient for the dataset was 0.526). The normalized mutual information content was 0.310. Causal Direction for this dataset was correctly predicted.

53 Dataset-53

ground truth:

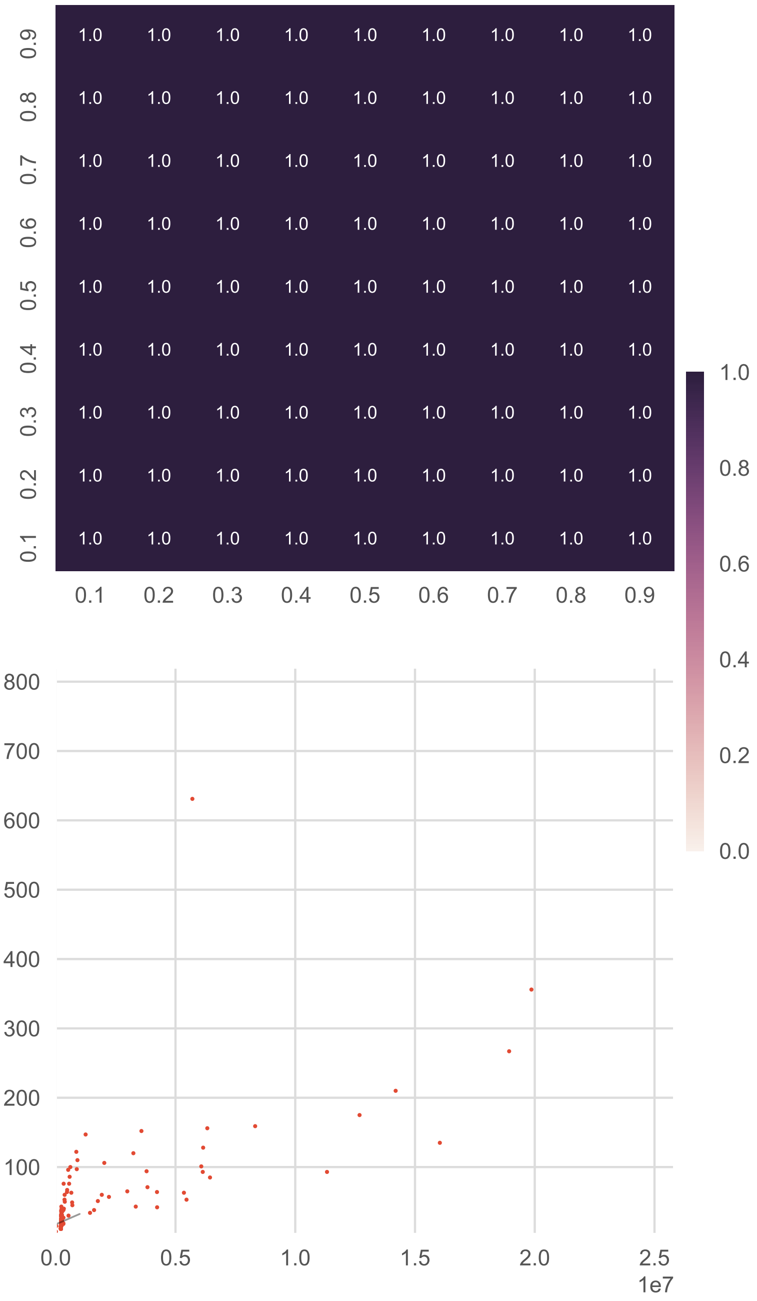

Figure 53: The fitted line was y = 17.430 + 0.000x. The Pearson Correlation Coefficient for the dataset was 0.709 with a p-value of 0.000. The Spearman Rank Correlation Coefficient for the dataset was 0.927. The Kendall Tau Rank Correlation Coefficient for the dataset was 0.840). The normalized mutual information content was 0.498. Causal Direction for this dataset could not be predicted.

54 Dataset-54

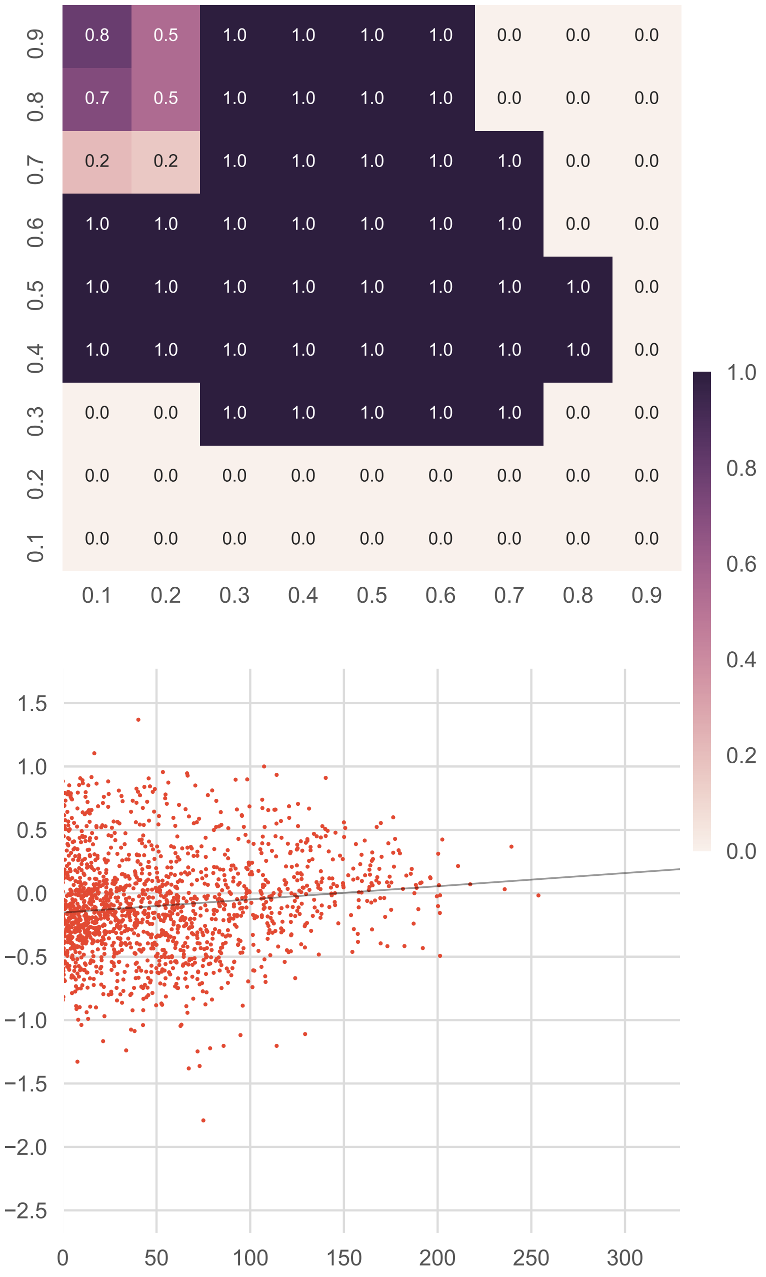

Figure 54: The fitted line was y = -0.152 + 0.001x. The Pearson Correlation Coefficient for the dataset was 0.125 with a p-value of 0.000. The Spearman Rank Correlation Coefficient for the dataset was 0.122. The Kendall Tau Rank Correlation Coefficient for the dataset was 0.081). The normalized mutual information content was 0.253. Causal Direction for this dataset was incorrectly predicted.

55 Dataset-55

x: GNI (Gross national income) per capita for different countries (in US$)
y: life expectancy at birth for different countries
ground truth:
x --> y

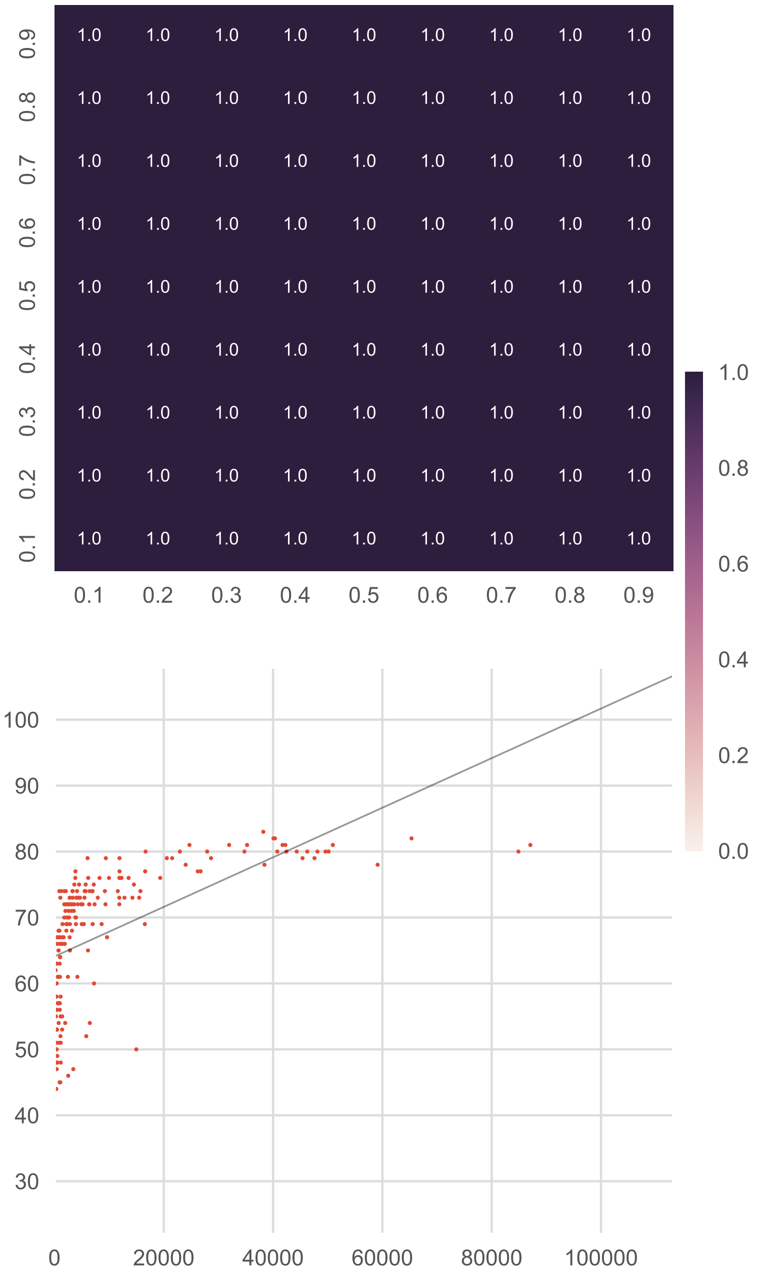

Figure 55: The fitted line was y = 64.086 + 0.000x. The Pearson Correlation Coefficient for the dataset was 0.582 with a p-value of 0.000. The Spearman Rank Correlation Coefficient for the dataset was 0.838. The Kendall Tau Rank Correlation Coefficient for the dataset was 0.669). The normalized mutual information content was 0.489. Causal Direction for this dataset could not be predicted.

56 Dataset-56

x: under 5 mortality rate for different countries (deaths per 1000 live births)
y: GNI (Gross national income) per capita for different countries (in US$)
ground truth:
y --> x

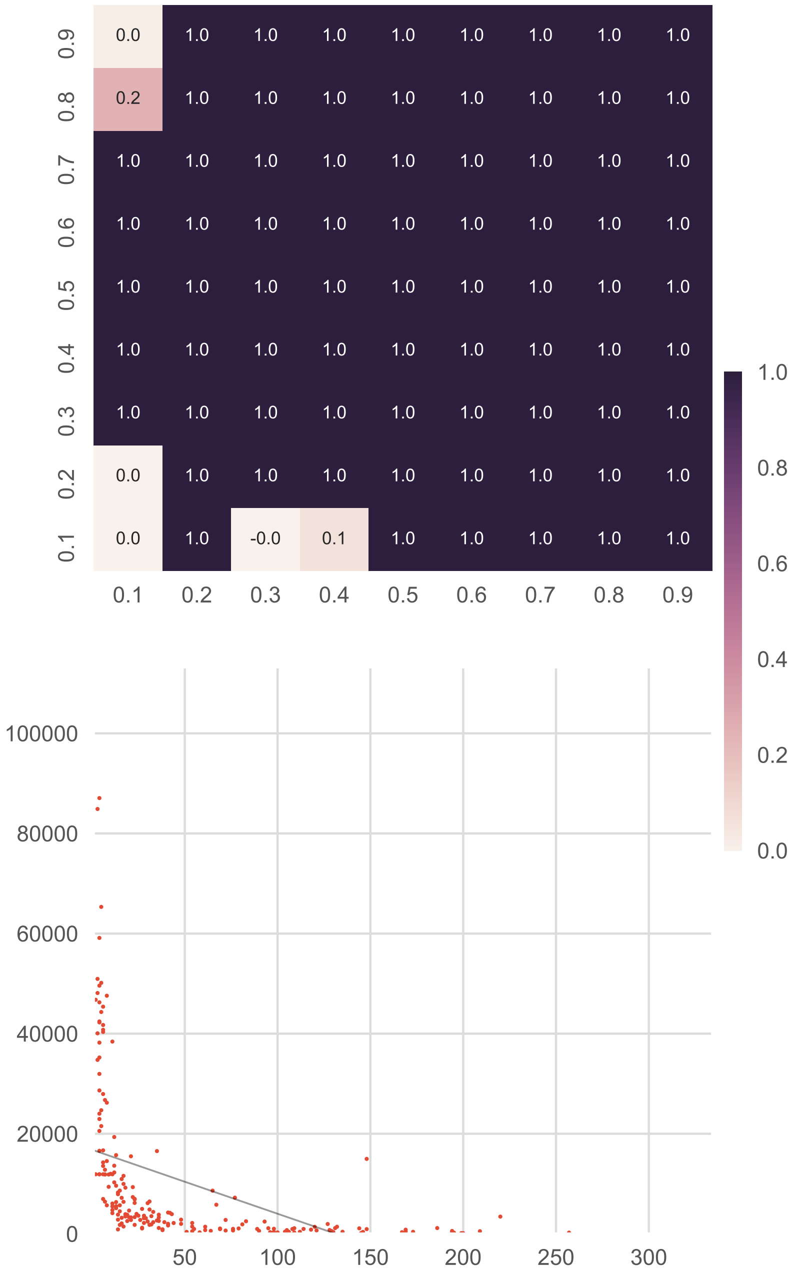

Figure 56: The fitted line was y = 16811.358 + -127.917x. The Pearson Correlation Coefficient for the dataset was -0.453 with a p-value of 0.000. The Spearman Rank Correlation Coefficient for the dataset was -0.877. The Kendall Tau Rank Correlation Coefficient for the dataset was -0.706). The normalized mutual information content was 0.483. Causal Direction for this dataset was incorrectly predicted.

57 Dataset-57

x (first column): the average annual rate of change of population;
y (second column): the average annual rate of change of total dietary consumption for total population (kcal/day).
x --> y

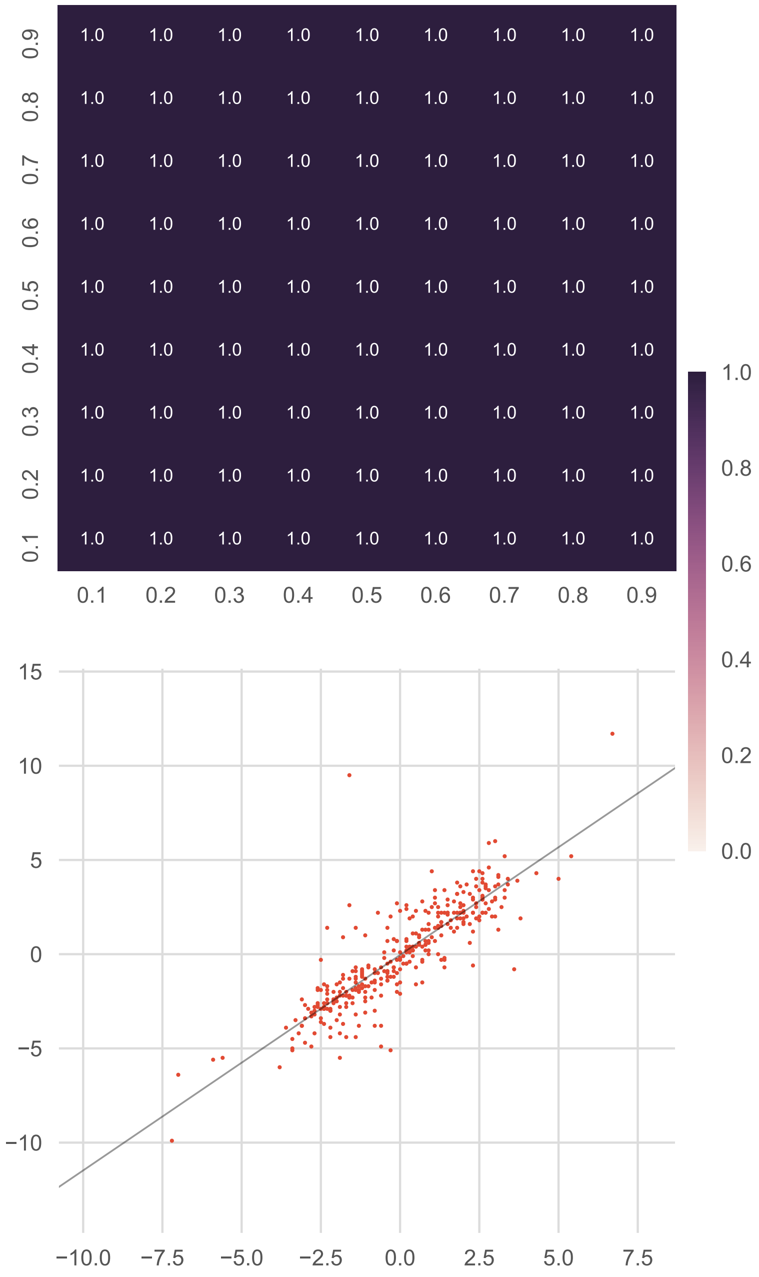

Figure 57: The fitted line was y = -0.041 + 1.144x. The Pearson Correlation Coefficient for the dataset was 0.864 with a p-value of 0.000. The Spearman Rank Correlation Coefficient for the dataset was 0.881. The Kendall Tau Rank Correlation Coefficient for the dataset was 0.721). The normalized mutual information content was 0.553. Causal Direction for this dataset could not be predicted.

58 Dataset-58

x -> y

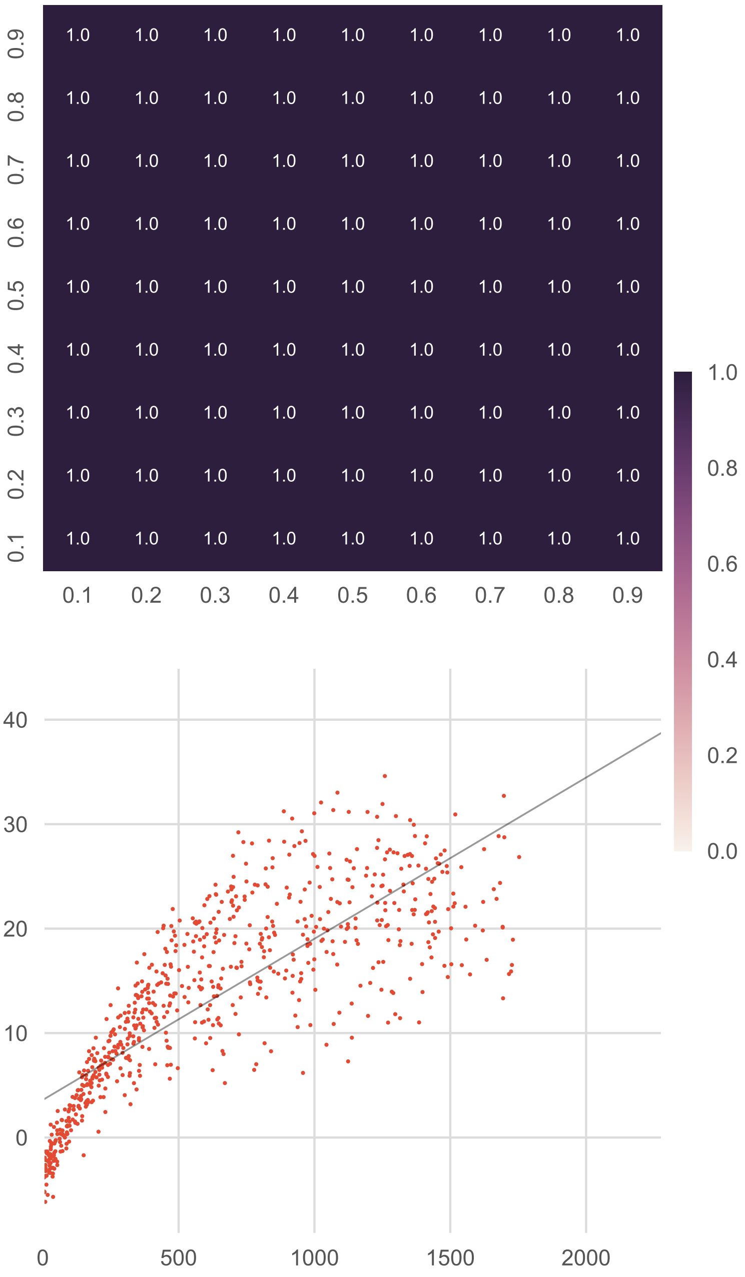

Figure 58: The fitted line was y = 3.597 + 0.015x. The Pearson Correlation Coefficient for the dataset was 0.806 with a p-value of 0.000. The Spearman Rank Correlation Coefficient for the dataset was 0.861. The Kendall Tau Rank Correlation Coefficient for the dataset was 0.690). The normalized mutual information content was 0.563. Causal Direction for this dataset could not be predicted.

59 Dataset-59

y -> x

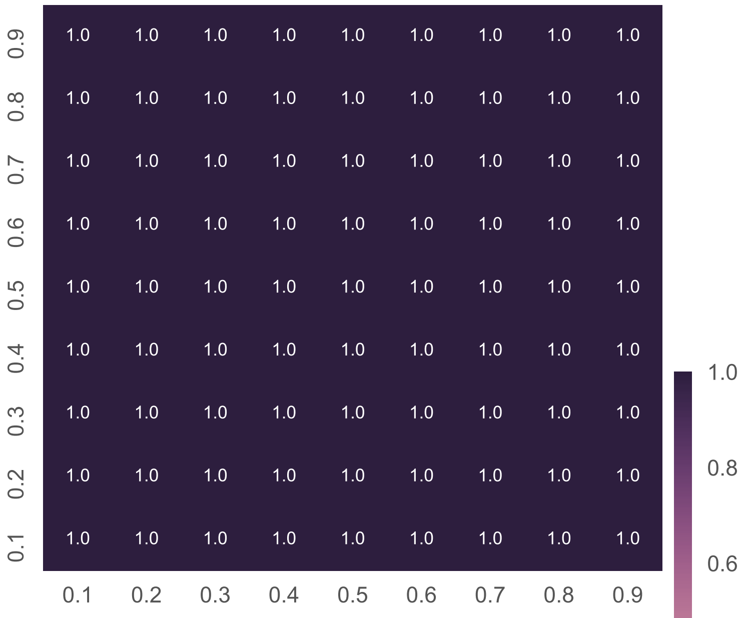

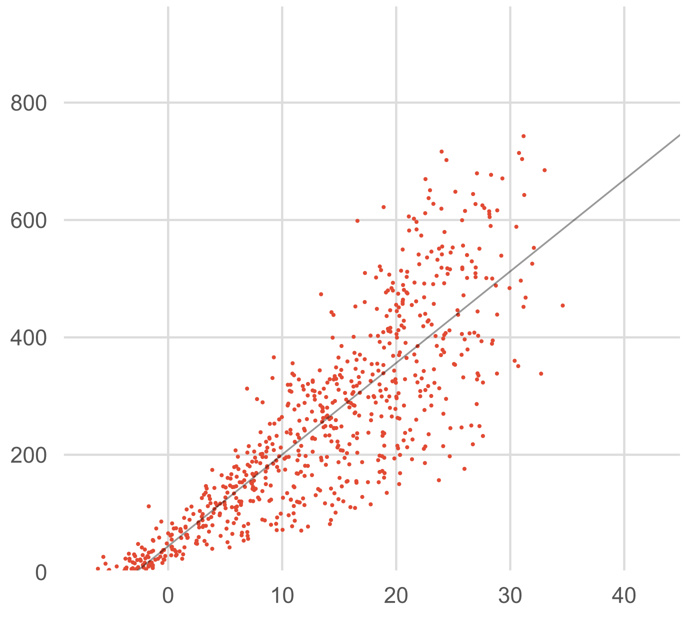

Figure 59: The fitted line was y = 44.287 + 15.606x. The Pearson Correlation Coefficient for the dataset was 0.838 with a p-value of 0.000. The Spearman Rank Correlation Coefficient for the dataset was 0.863. The Kendall Tau Rank Correlation Coefficient for the dataset was 0.683). The normalized mutual information content was 0.541. Causal Direction for this dataset could not be predicted.

60 Dataset-60

y -> x

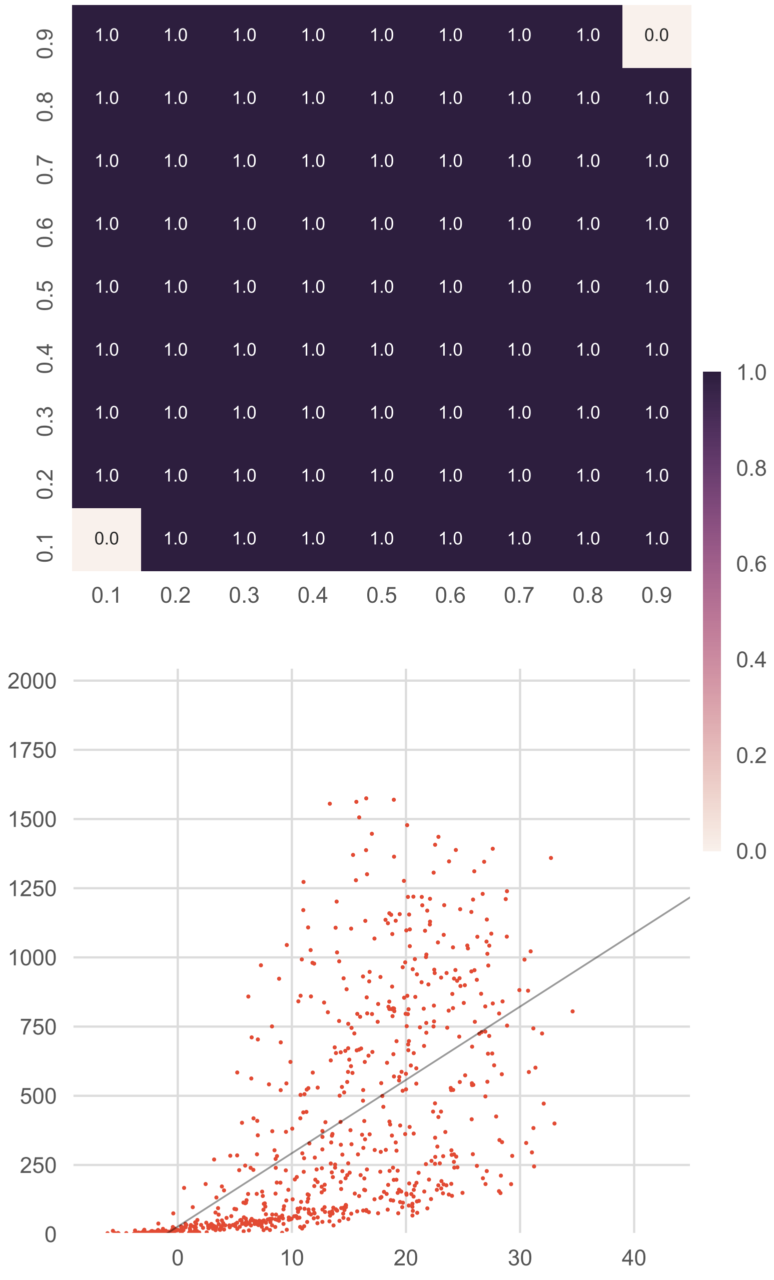

Figure 60: The fitted line was y = 27.651 + 26.488x. The Pearson Correlation Coefficient for the dataset was 0.600 with a p-value of 0.000. The Spearman Rank Correlation Coefficient for the dataset was 0.745. The Kendall Tau Rank Correlation Coefficient for the dataset was 0.558). The normalized mutual information content was 0.518. Causal Direction for this dataset could not be predicted.

61 Dataset-61

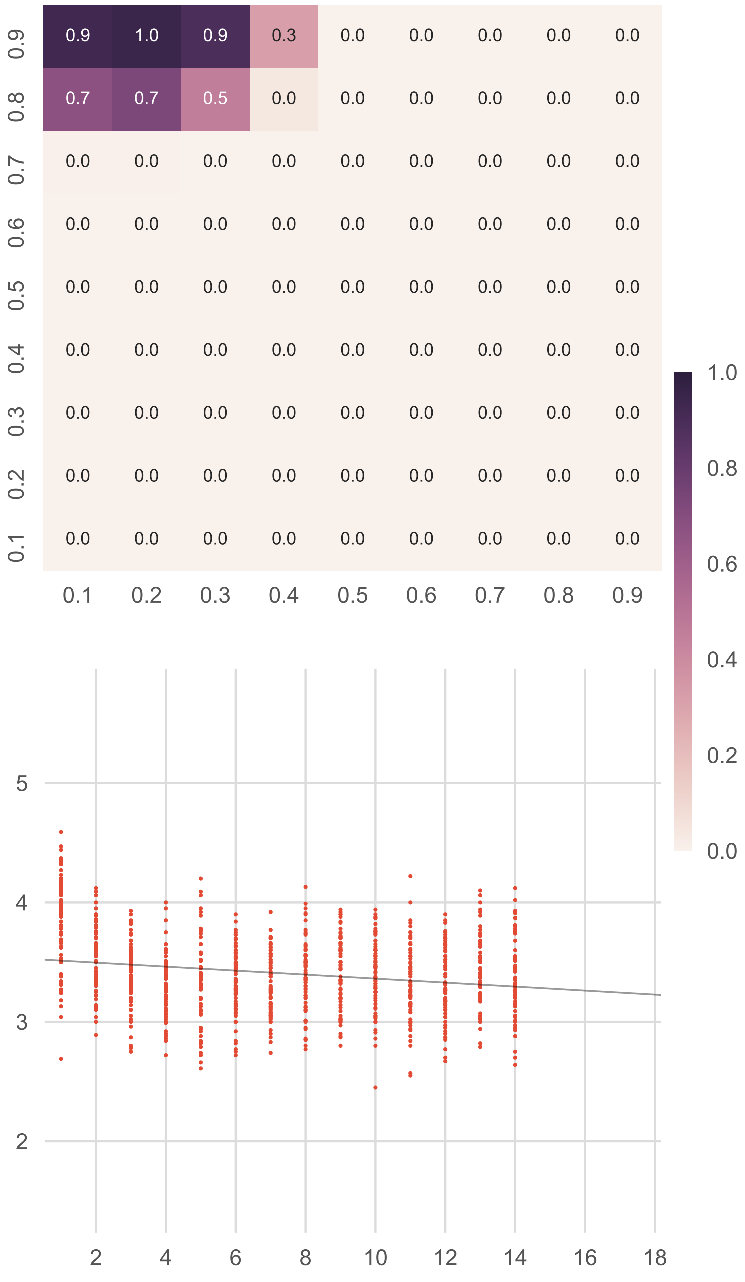

Figure 61: The fitted line was y = 3.529 + -0.017x. The Pearson Correlation Coefficient for the dataset was -0.205 with a p-value of 0.000. The Spearman Rank Correlation Coefficient for the dataset was -0.176. The Kendall Tau Rank Correlation Coefficient for the dataset was -0.124). The normalized mutual information content was 0.172. Causal Direction for this dataset was incorrectly predicted.

62 Dataset-62

x —> y

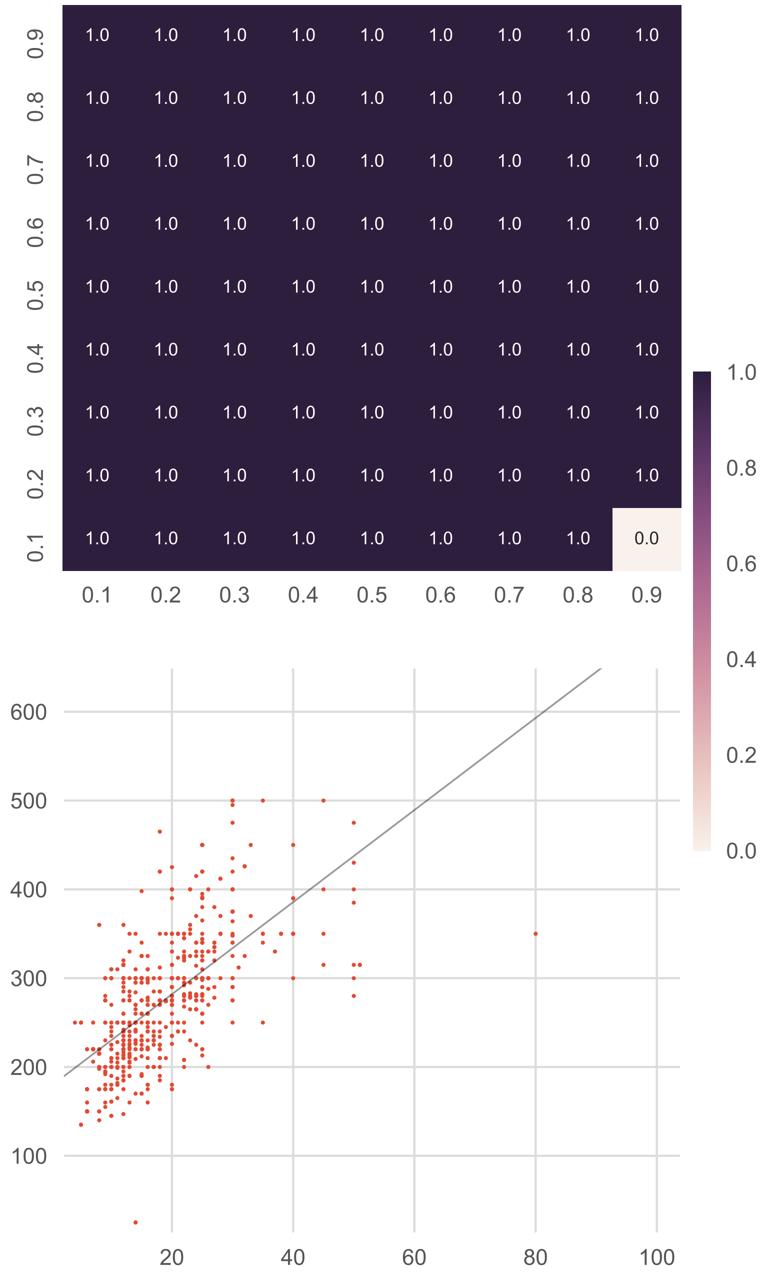

Figure 62: The fitted line was y = 178.265 + 5.184x. The Pearson Correlation Coefficient for the dataset was 0.633 with a p-value of 0.000. The Spearman Rank Correlation Coefficient for the dataset was 0.670. The Kendall Tau Rank Correlation Coefficient for the dataset was 0.505). The normalized mutual information content was 0.384. Causal Direction for this dataset could not be predicted.

63 Dataset-63

y = Relative Spinal bone mineral density
x —> y

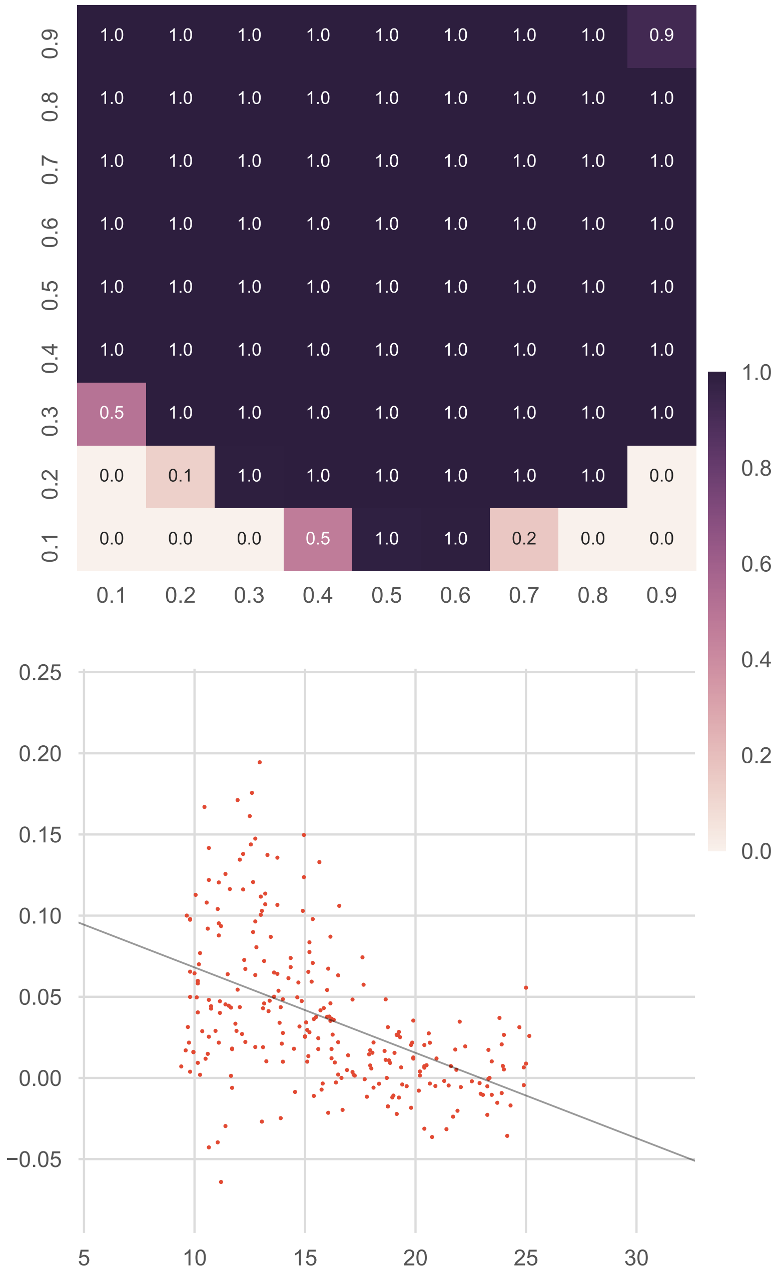

Figure 63: The fitted line was y = 0.121 + -0.005x. The Pearson Correlation Coefficient for the dataset was -0.487 with a p-value of 0.000. The Spearman Rank Correlation Coefficient for the dataset was -0.531. The Kendall Tau Rank Correlation Coefficient for the dataset was -0.369). The normalized mutual information content was 0.690. Causal Direction for this dataset was correctly predicted.

64 Dataset-64

y = Mass loss APRIL 2012 in %
y denotes the mass loss 6 months after the start of the experiment.
y denotes the mass loss 1 year after the start of the experiment.
y --> x

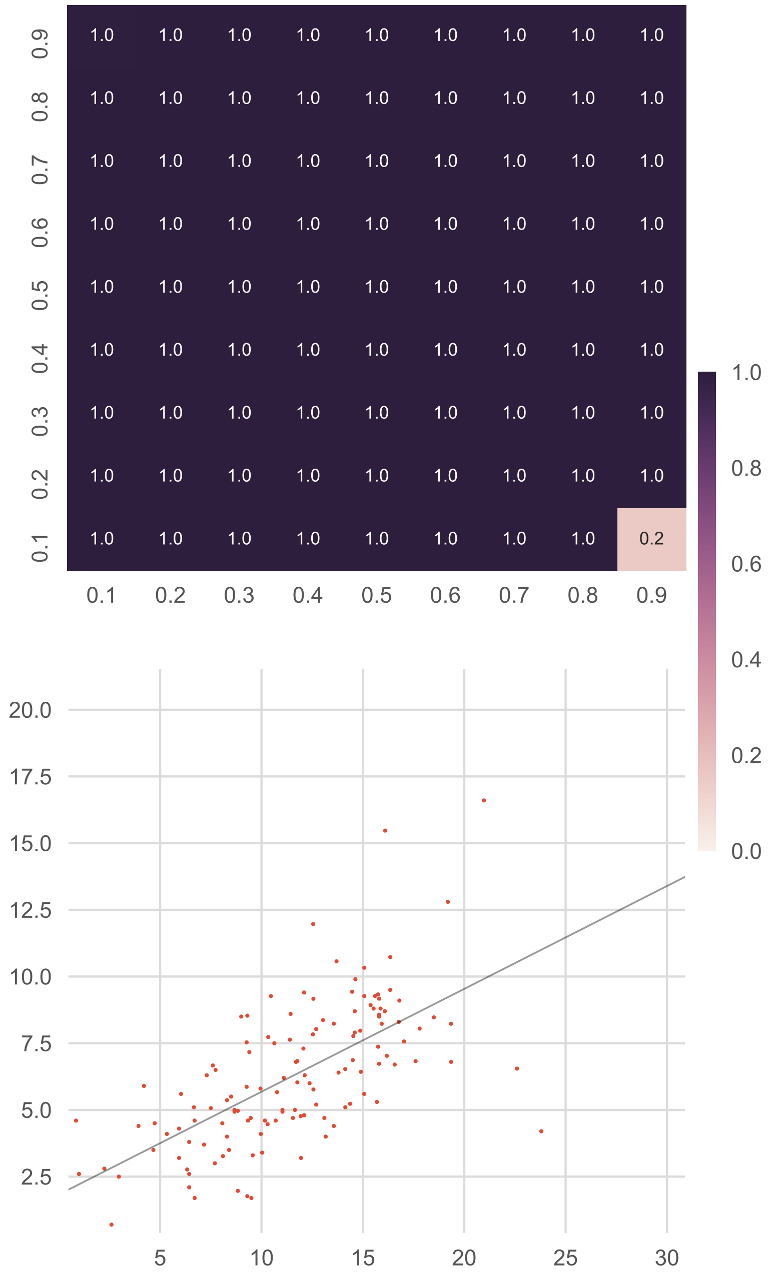

Figure 64: The fitted line was y = 1.826 + 0.386x. The Pearson Correlation Coefficient for the dataset was 0.639 with a p-value of 0.000. The Spearman Rank Correlation Coefficient for the dataset was 0.690. The Kendall Tau Rank Correlation Coefficient for the dataset was 0.490). The normalized mutual information content was 0.757. Causal Direction for this dataset could not be predicted.

65 Dataset-65

y = Mass loss APRIL 2012 in %
y denotes the mass loss 6 months after the start of the experiment.
y denotes the mass loss 1 year after the start of the experiment.
y --> x

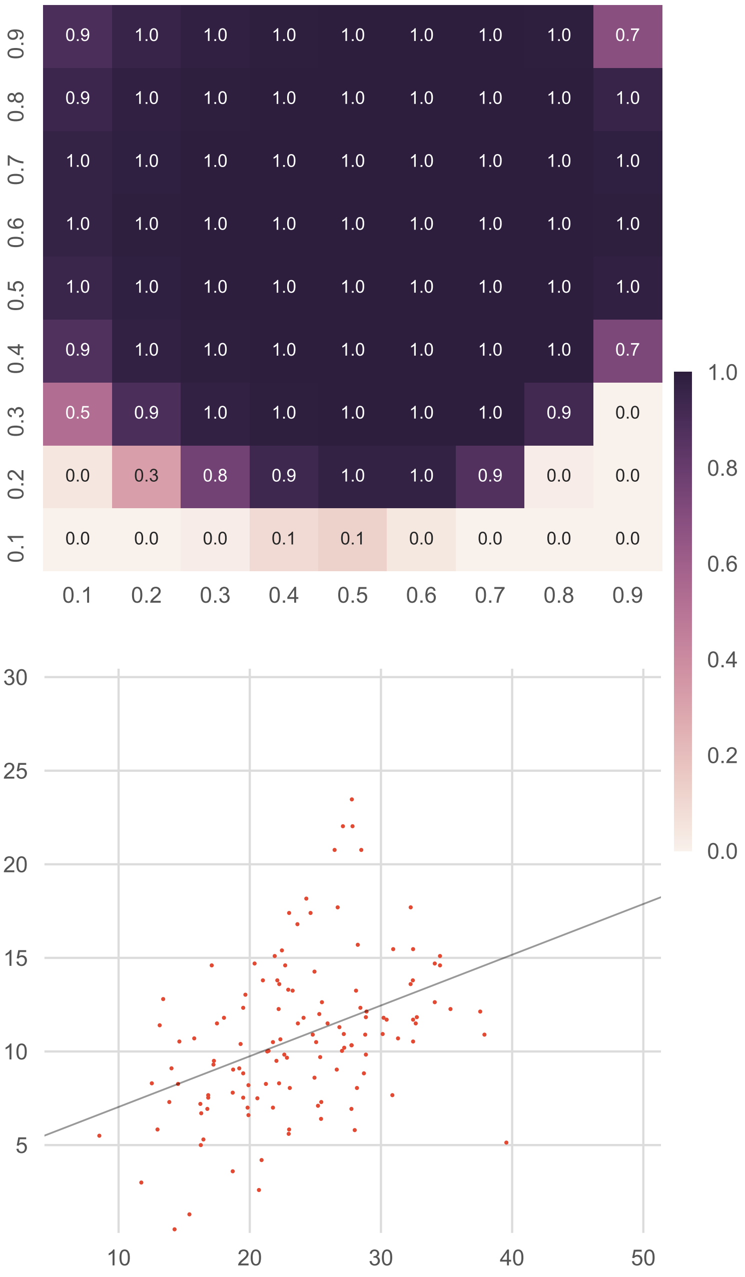

Figure 65: The fitted line was y = 4.326 + 0.271x. The Pearson Correlation Coefficient for the dataset was 0.408 with a p-value of 0.000. The Spearman Rank Correlation Coefficient for the dataset was 0.451. The Kendall Tau Rank Correlation Coefficient for the dataset was 0.312). The normalized mutual information content was 0.754. Causal Direction for this dataset was incorrectly predicted.

66 Dataset-66

y = Soil moisture at 10cm depth (in %)
x --> y

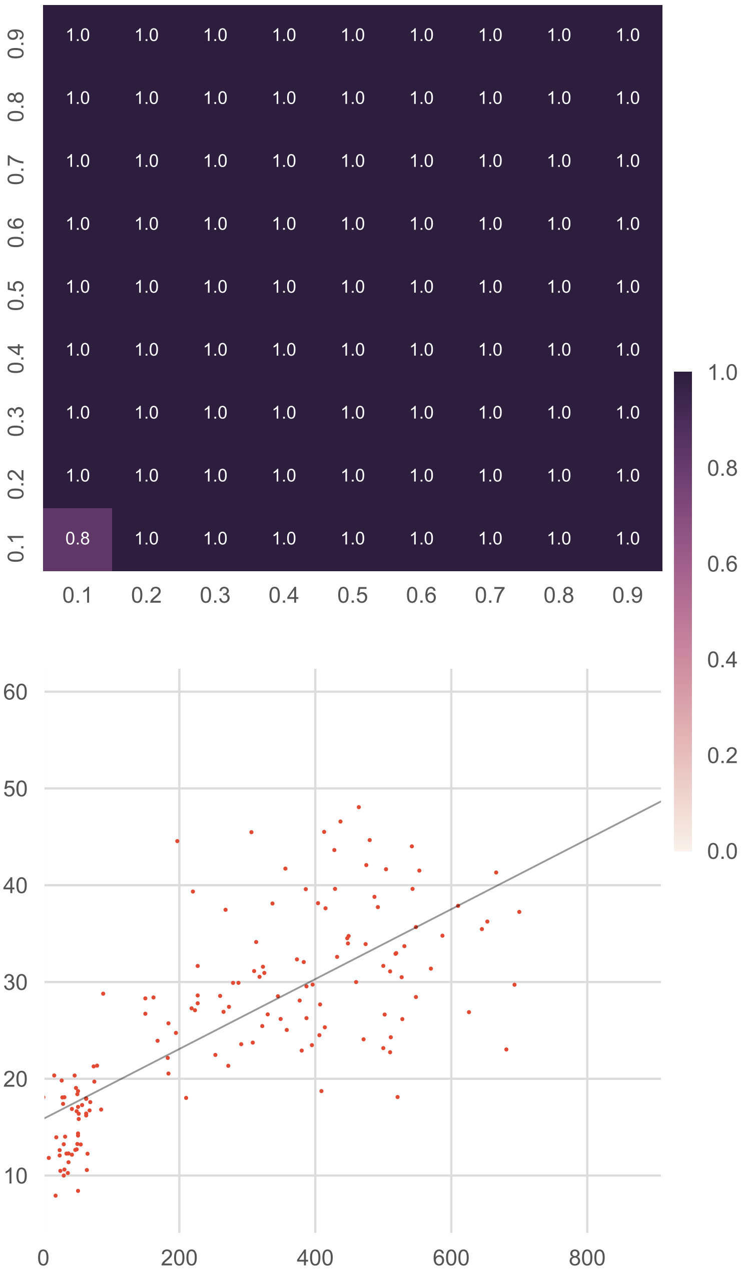

Figure 66: The fitted line was y = 15.854 + 0.036x. The Pearson Correlation Coefficient for the dataset was 0.746 with a p-value of 0.000. The Spearman Rank Correlation Coefficient for the dataset was 0.767. The Kendall Tau Rank Correlation Coefficient for the dataset was 0.565). The normalized mutual information content was 0.782. Causal Direction for this dataset was correctly predicted.

67 Dataset-67

y = Clay content (in g/kg)
y --> x

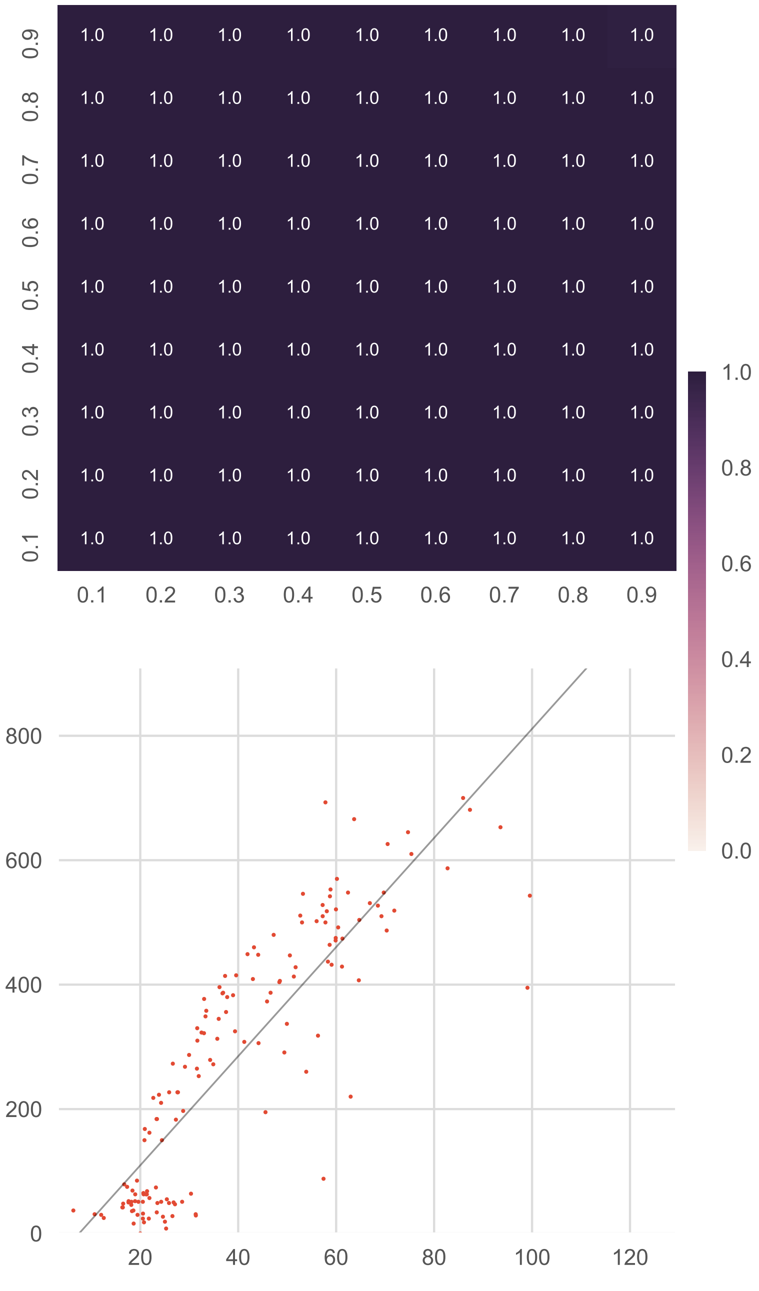

Figure 67: The fitted line was y = -65.832 + 8.770x. The Pearson Correlation Coefficient for the dataset was 0.867 with a p-value of 0.000. The Spearman Rank Correlation Coefficient for the dataset was 0.886. The Kendall Tau Rank Correlation Coefficient for the dataset was 0.707). The normalized mutual information content was 0.775. Causal Direction for this dataset could not be predicted.

68 Dataset-68

y = average runoff in over 1948 to 2004 mm/day
x --> y

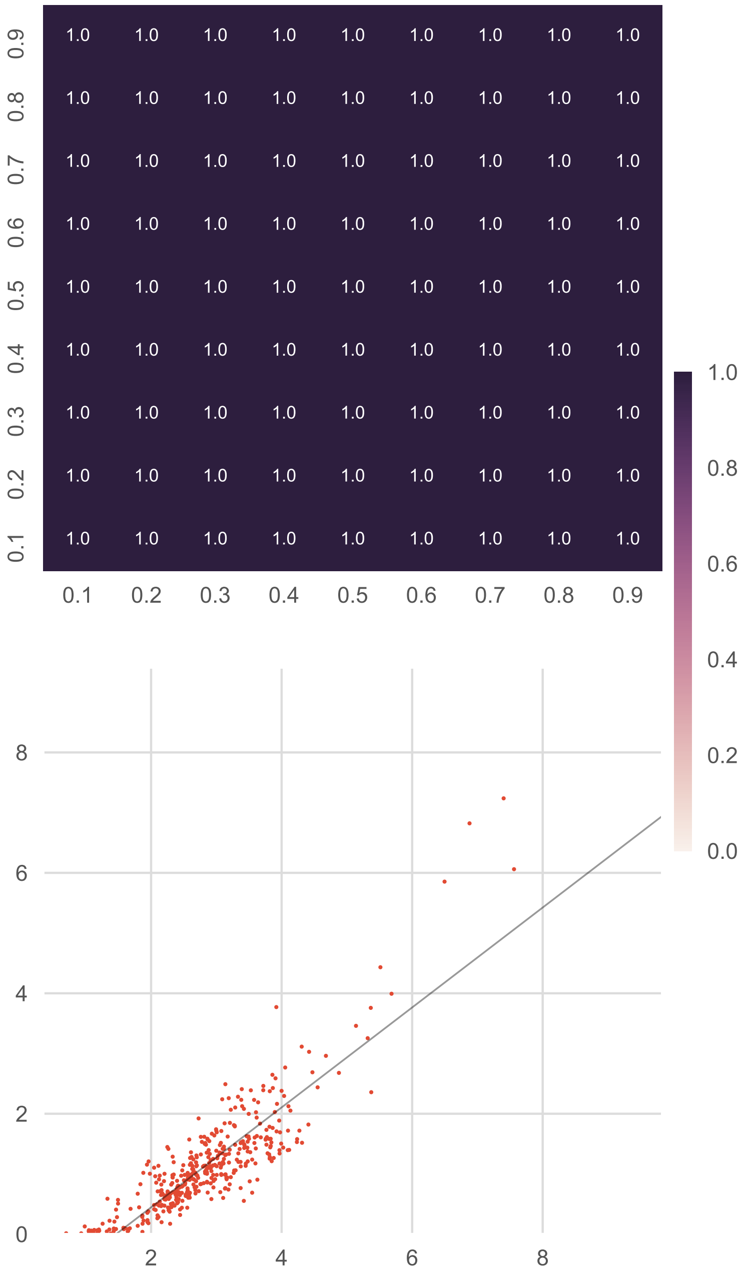

Figure 68: The fitted line was y = -1.214 + 0.830x. The Pearson Correlation Coefficient for the dataset was 0.874 with a p-value of 0.000. The Spearman Rank Correlation Coefficient for the dataset was 0.849. The Kendall Tau Rank Correlation Coefficient for the dataset was 0.670). The normalized mutual information content was 0.473. Causal Direction for this dataset could not be predicted.

69 Dataset-69

x - - > y

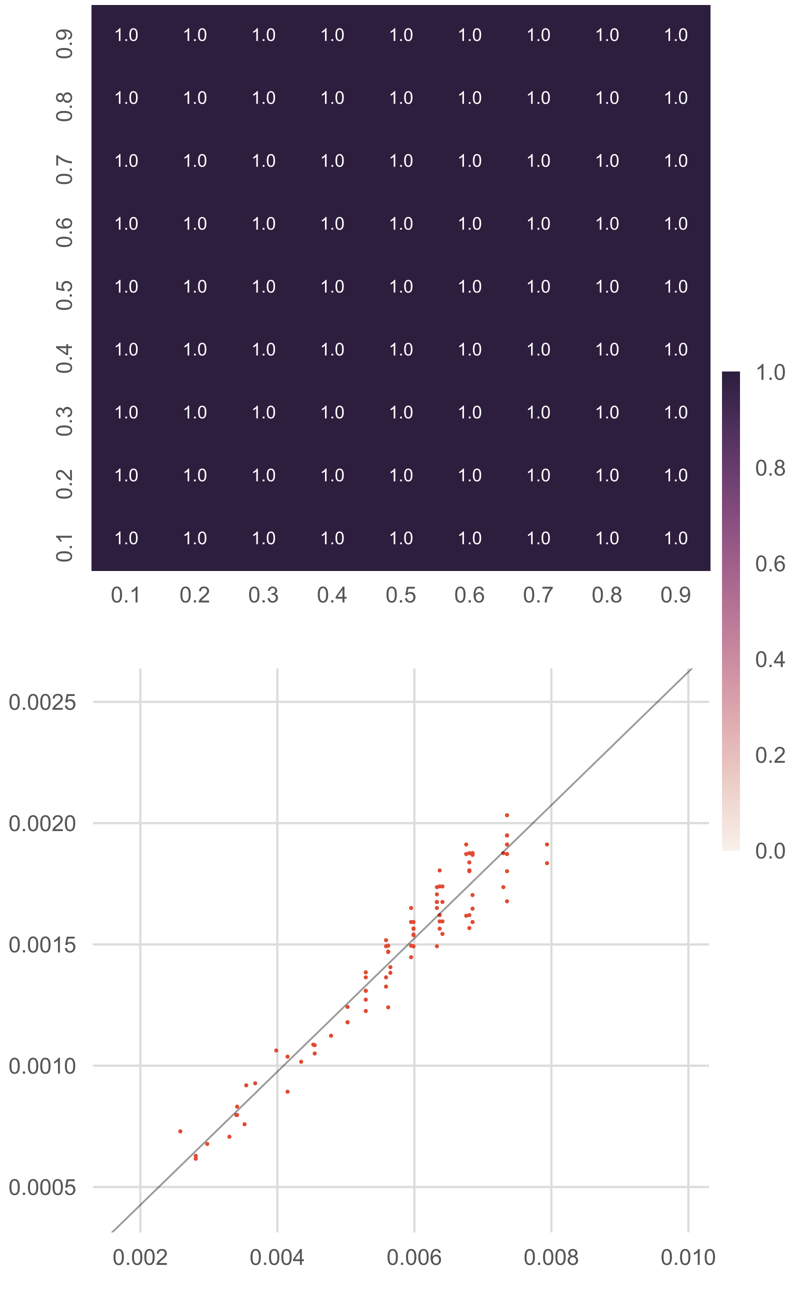

Figure 69: The fitted line was y = -0.000 + 0.275x. The Pearson Correlation Coefficient for the dataset was 0.968 with a p-value of 0.000. The Spearman Rank Correlation Coefficient for the dataset was 0.943. The Kendall Tau Rank Correlation Coefficient for the dataset was 0.828). The normalized mutual information content was 0.729. Causal Direction for this dataset could not be predicted.

70 Dataset-70

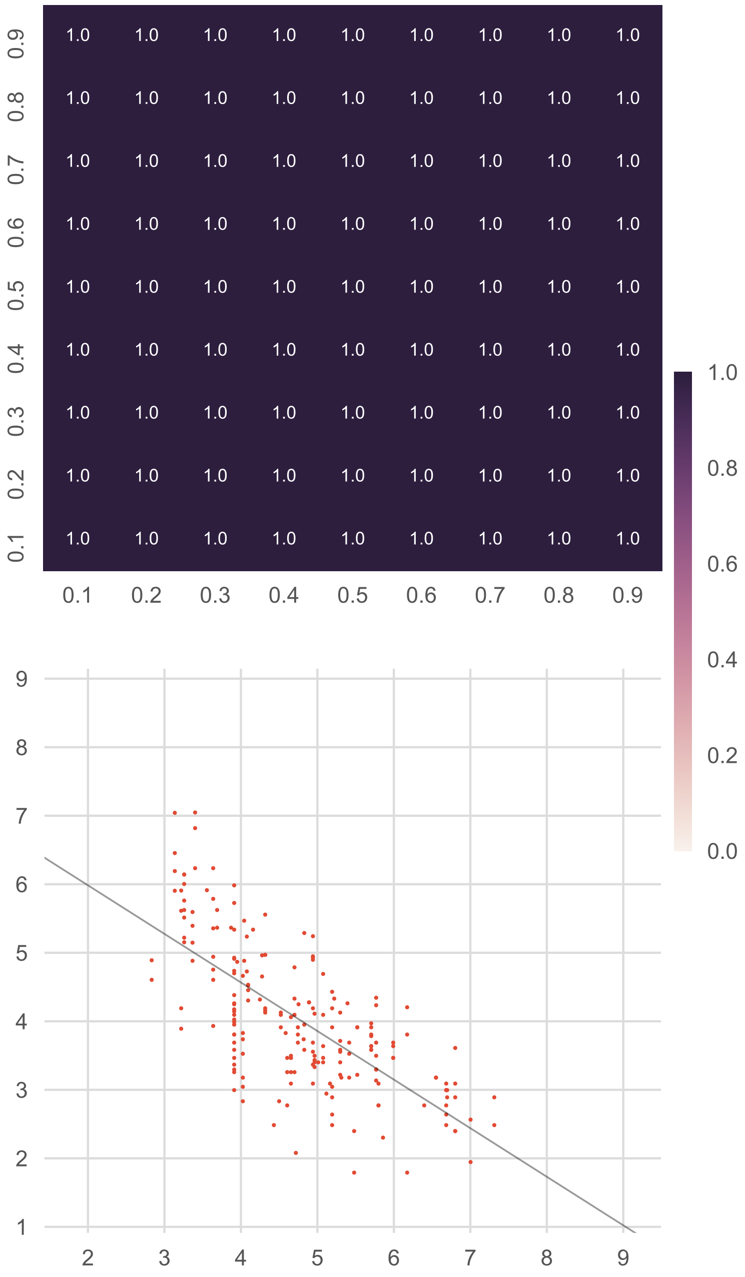

Figure 70: The fitted line was y = 7.402 + -0.709x. The Pearson Correlation Coefficient for the dataset was -0.702 with a p-value of 0.000. The Spearman Rank Correlation Coefficient for the dataset was -0.692. The Kendall Tau Rank Correlation Coefficient for the dataset was -0.516). The normalized mutual information content was 0.633. Causal Direction for this dataset could not be predicted.

71 Dataset-71

ground truth:
x->y

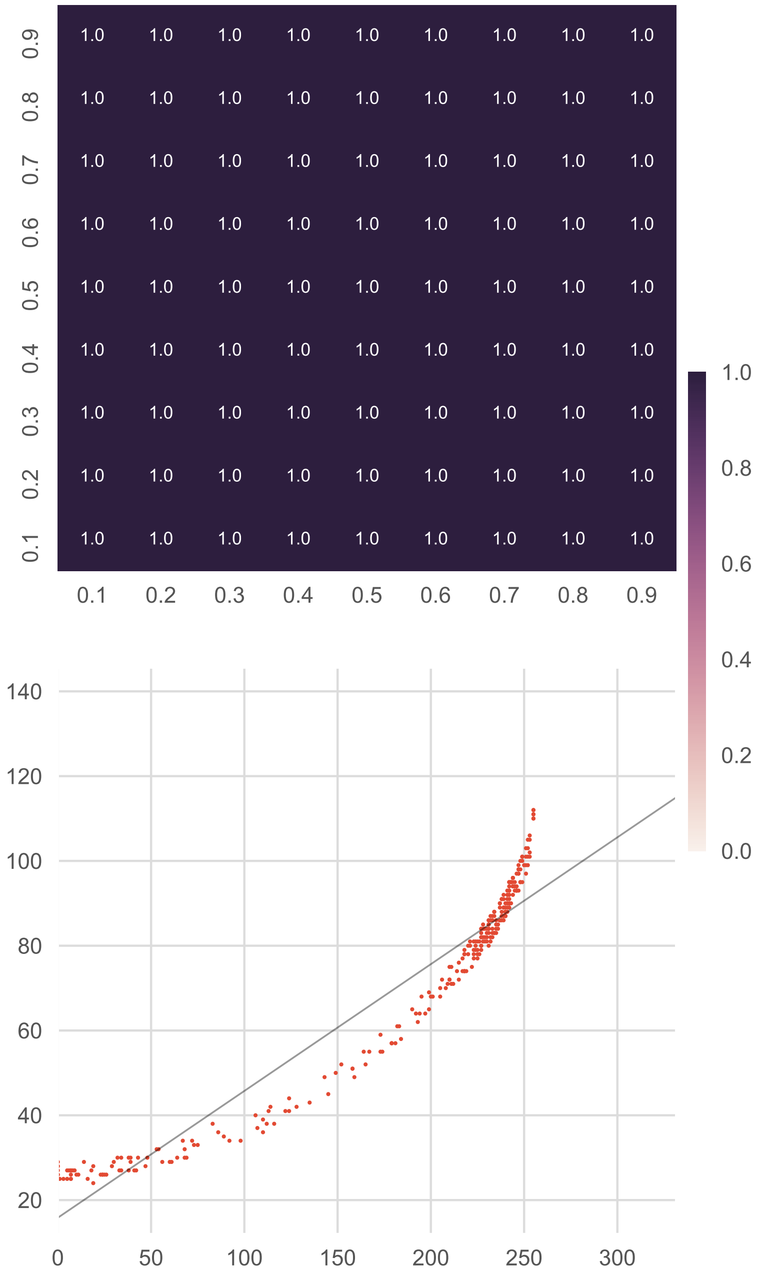

Figure 71: The fitted line was y = 15.835 + 0.299x. The Pearson Correlation Coefficient for the dataset was 0.963 with a p-value of 0.000. The Spearman Rank Correlation Coefficient for the dataset was 0.991. The Kendall Tau Rank Correlation Coefficient for the dataset was 0.933). The normalized mutual information content was 0.707. Causal Direction for this dataset could not be predicted.

72 Dataset-72

ground truth:
x->y

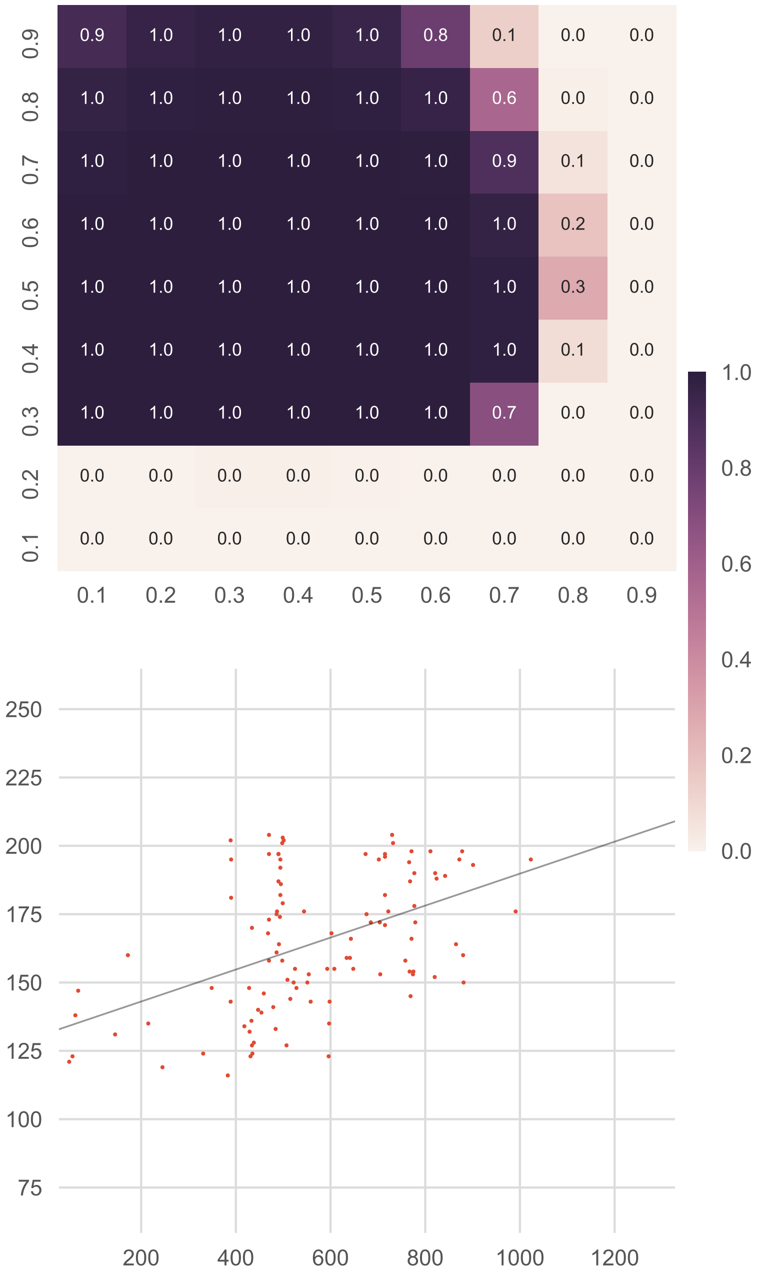

Figure 72: The fitted line was y = 131.346 + 0.058x. The Pearson Correlation Coefficient for the dataset was 0.470 with a p-value of 0.000. The Spearman Rank Correlation Coefficient for the dataset was 0.444. The Kendall Tau Rank Correlation Coefficient for the dataset was 0.315). The normalized mutual information content was 0.777. Causal Direction for this dataset was incorrectly predicted.

73 Dataset-73

ground truth:
x->y

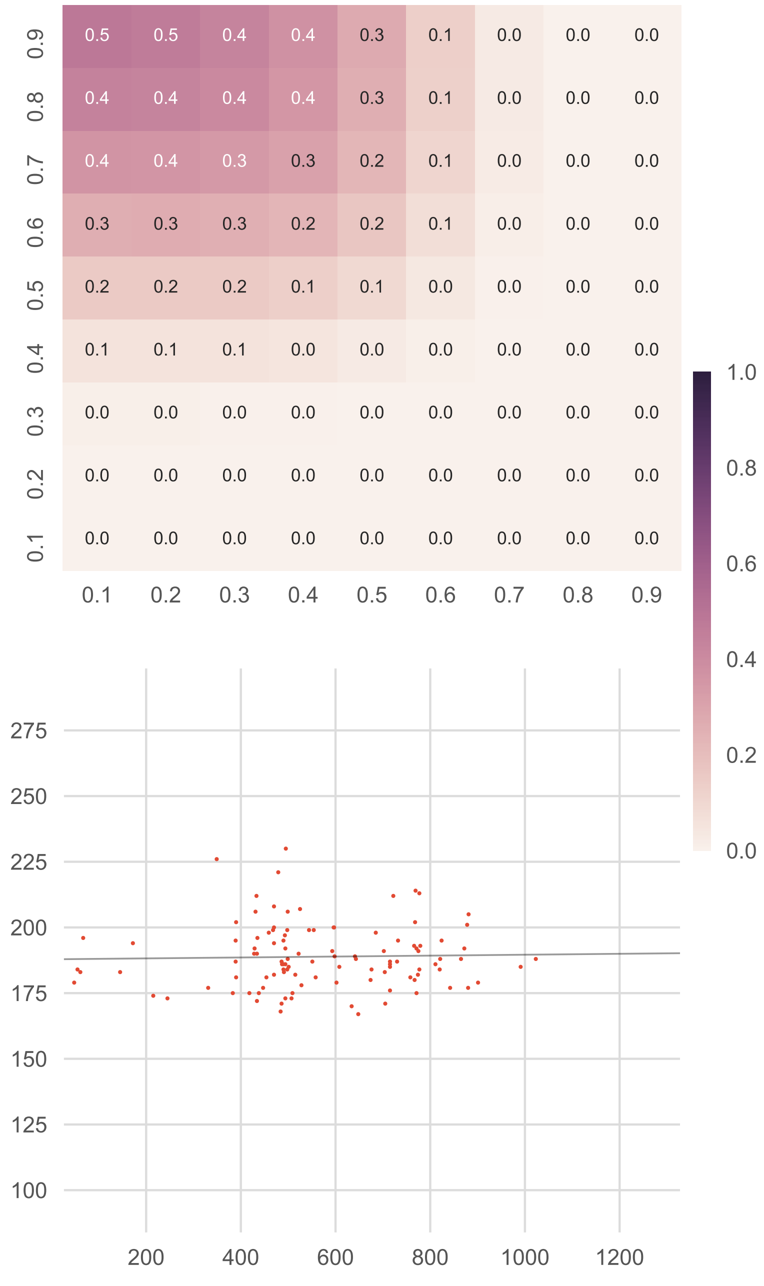

Figure 73: The fitted line was y = 187.871 + 0.002x. The Pearson Correlation Coefficient for the dataset was 0.029 with a p-value of 0.767. The Spearman Rank Correlation Coefficient for the dataset was 0.047. The Kendall Tau Rank Correlation Coefficient for the dataset was 0.033). The normalized mutual information content was 0.719. Causal Direction for this dataset was correctly predicted.

74 Dataset-74

ground truth:
x->y

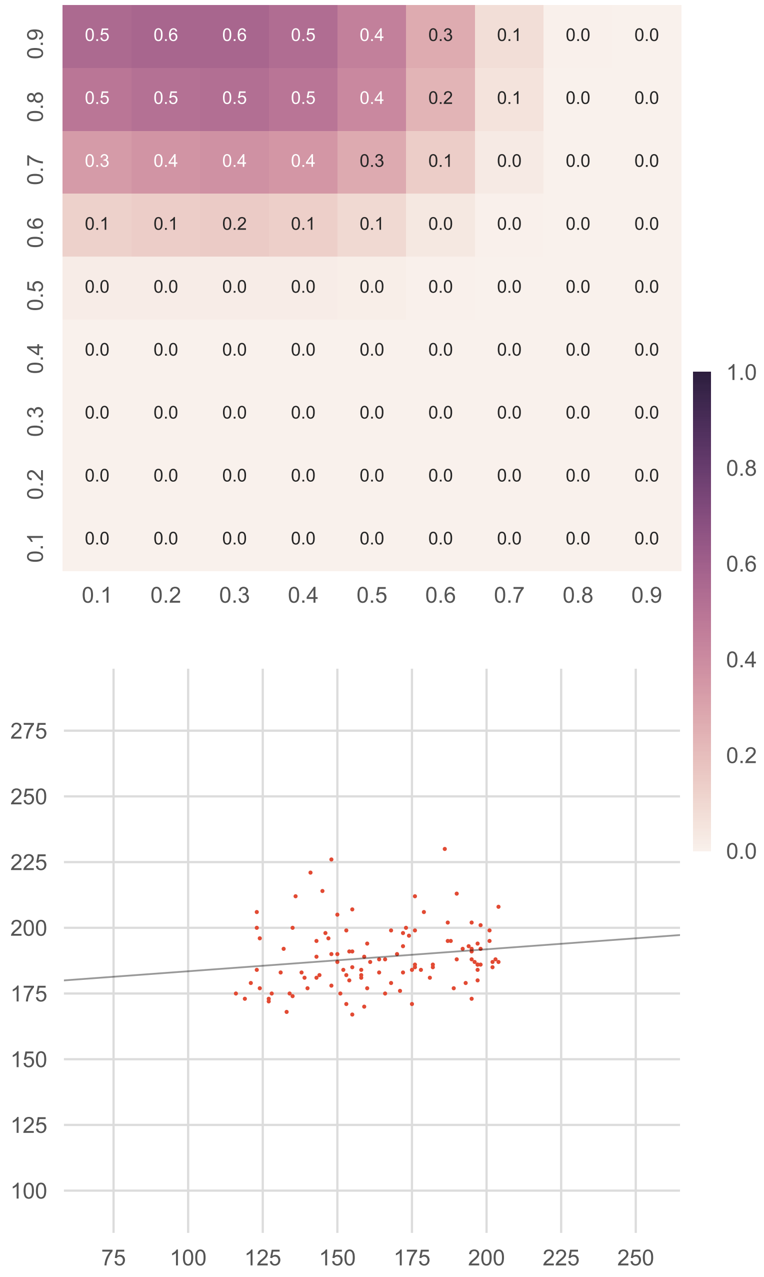

Figure 74: The fitted line was y = 175.102 + 0.084x. The Pearson Correlation Coefficient for the dataset was 0.171 with a p-value of 0.075. The Spearman Rank Correlation Coefficient for the dataset was 0.223. The Kendall Tau Rank Correlation Coefficient for the dataset was 0.154). The normalized mutual information content was 0.774. Causal Direction for this dataset was correctly predicted.

75 Dataset-75

ground truth:
y -> x

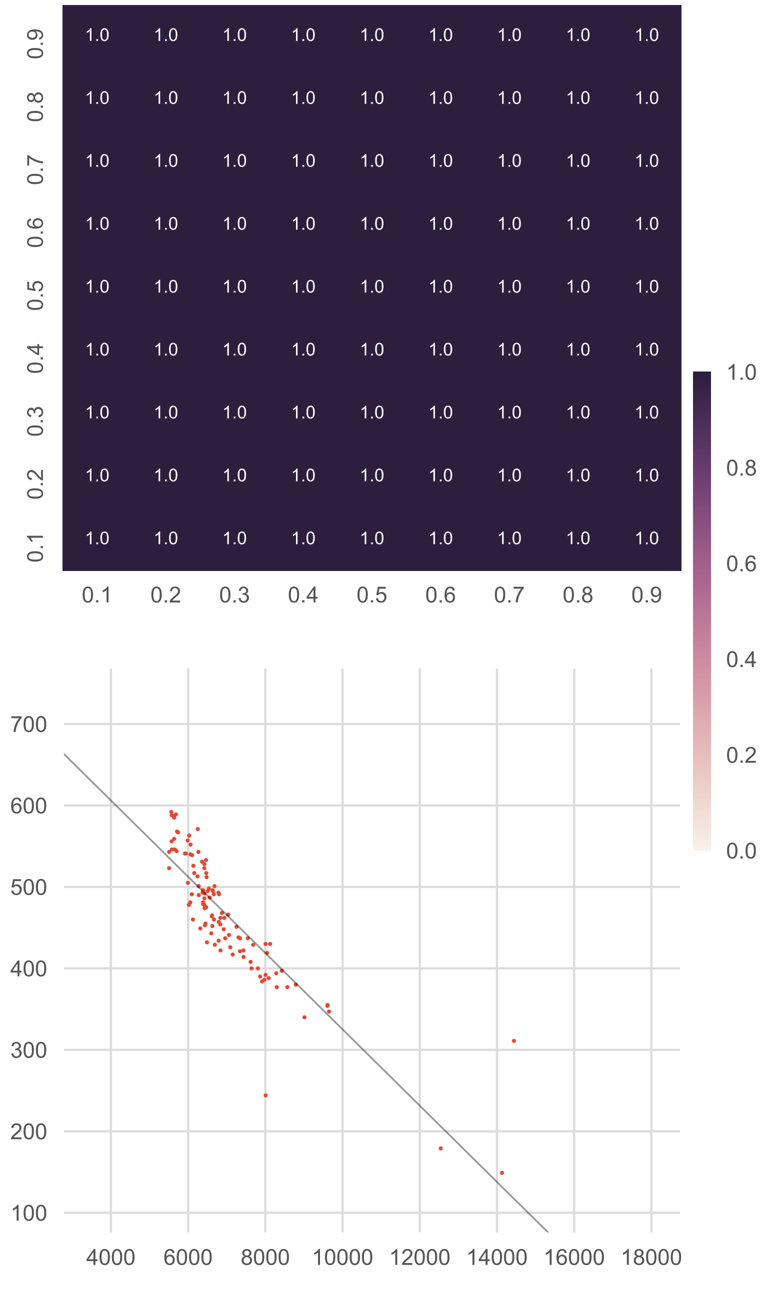

Figure 75: The fitted line was y = 793.488 + -0.047x. The Pearson Correlation Coefficient for the dataset was -0.870 with a p-value of 0.000. The Spearman Rank Correlation Coefficient for the dataset was -0.918. The Kendall Tau Rank Correlation Coefficient for the dataset was -0.764). The normalized mutual information content was 0.724. Causal Direction for this dataset could not be predicted.
