# Supplementary material for: A Bayesian measure of association that utilizes the underlying distributions of noise and information
Source: PLoS One. 2018 Aug 17;13(8):e0201185. doi: 10.1371/journal.pone.0201185 (PMC6097650; doi:10.1371/journal.pone.0201185)
Supplement: S4 File — The detailed results for the real world unassociated datasets are presented in the same manner as S3 File. (PDF) [file pone.0201185.s004.pdf]

# S4 File - Detailed Results on Unassociated datasets

All the datasets were taken from https://www.physionet.org/pn4/eegmmidb/. All the datasets was chosen from EEG recordings of different individuals. As every pair of recordings had recordings from two different individuals, the recordings were unassociated . We have calculated Pearson Correlation (PC), Spearman Rank Correlation (SR), Kendall Tau Rank Correlation (KT), Normalized Mutual Information Content (MI) and the Bayesian Probability Of Association (BPA) for all the datasets.

# 1 Dataset-1

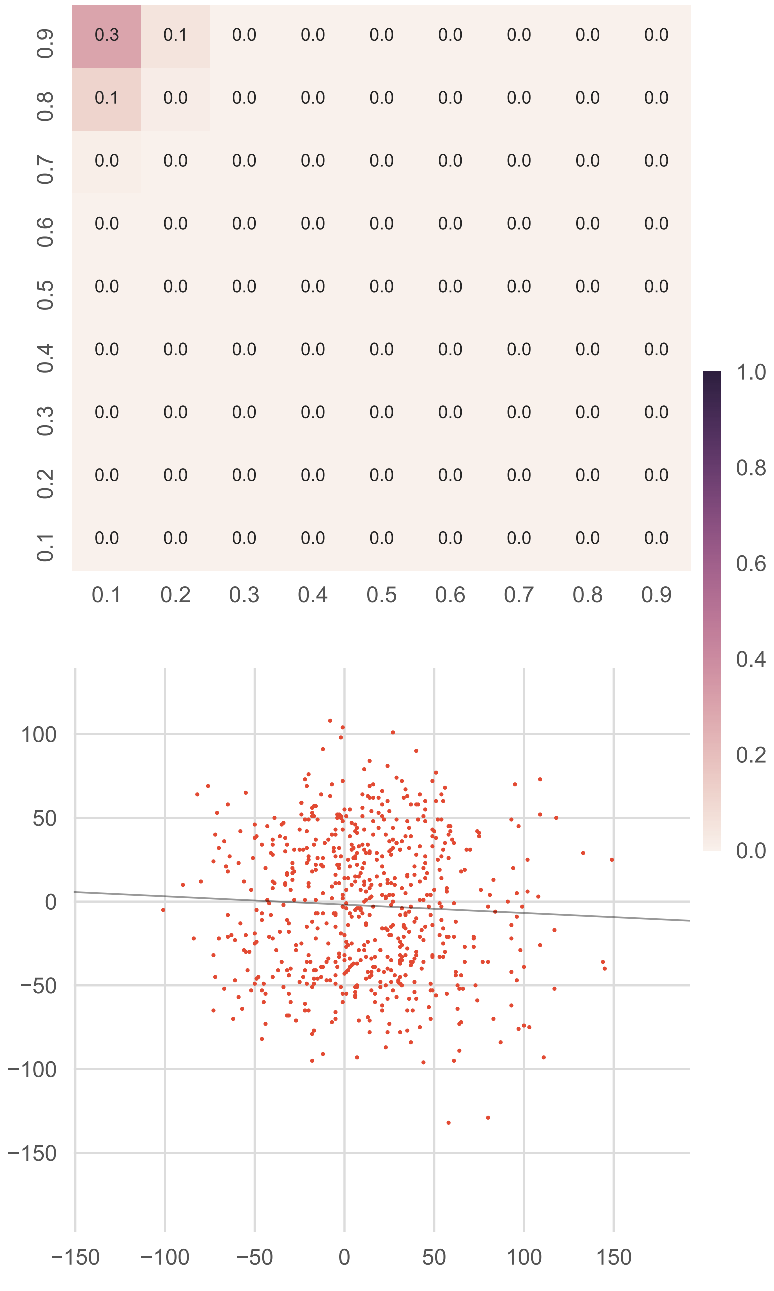




Figure 1: The fitted line was y = -1.817 + -0.050x. The Pearson Correlation Coefficient for the dataset was -0.048 with a p-value of 0.216. The Spearman Rank Correlation Coefficient for the dataset was -0.036. The Kendall Tau Rank Correlation Coefficient for the dataset was -0.023). The normalized mutual information content was 0.455.

## 2 Dataset-2

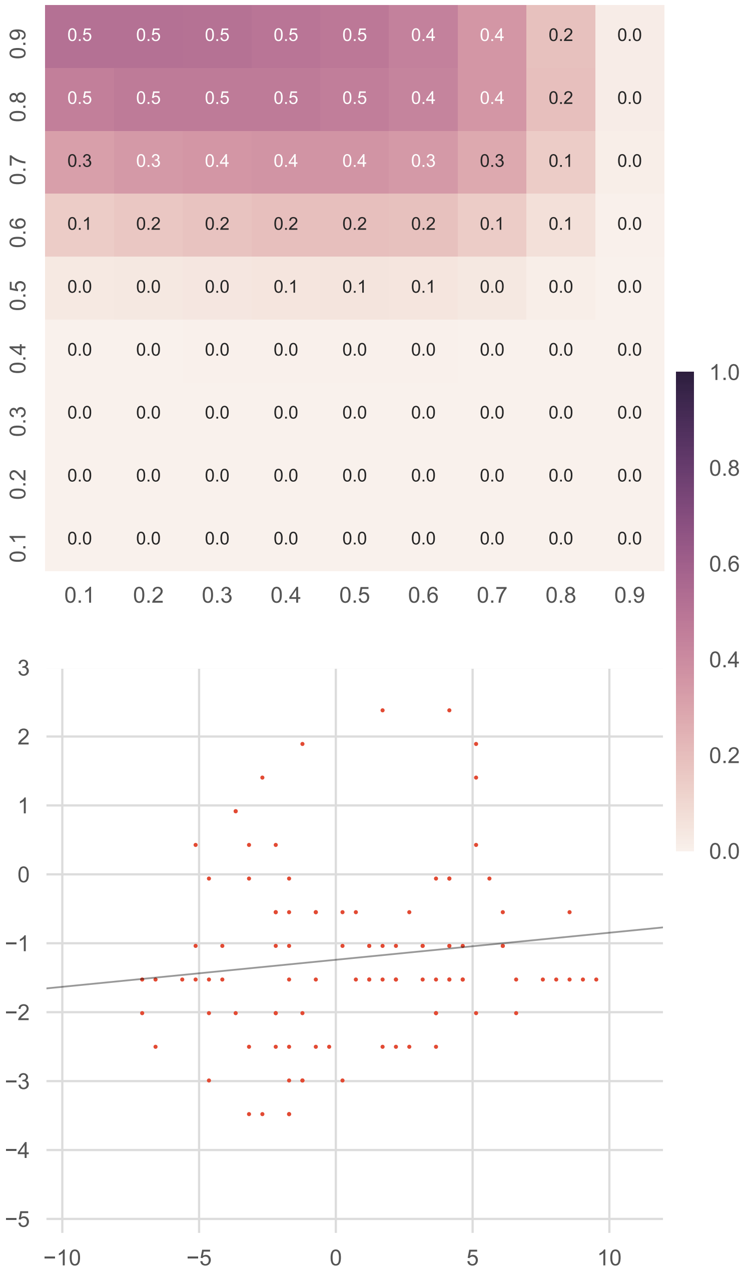


Figure 2: The fitted line was y = -1.239 + 0.039x. The Pearson Correlation Coefficient for the dataset was 0.128 with a p-value of 0.204. The Spearman Rank Correlation Coefficient for the dataset was 0.137. The Kendall Tau Rank Correlation Coefficient for the dataset was 0.093). The normalized mutual information content was 0.436.

## 3 Dataset-3

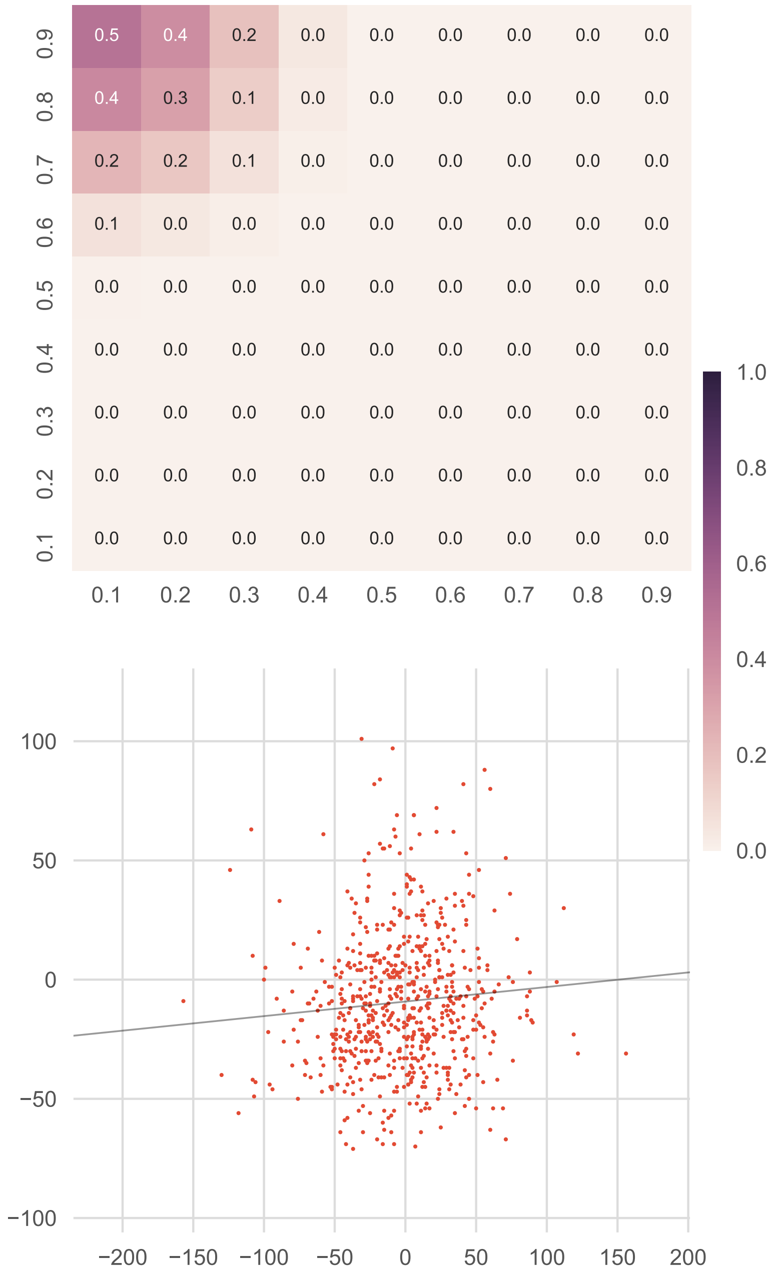




Figure 3: The fitted line was y = -9.200 + 0.061x. The Pearson Correlation Coefficient for the dataset was 0.083 with a p-value of 0.034. The Spearman Rank Correlation Coefficient for the dataset was 0.098. The Kendall Tau Rank Correlation Coefficient for the dataset was 0.067). The normalized mutual information content was 0.399.

## 4 Dataset-4

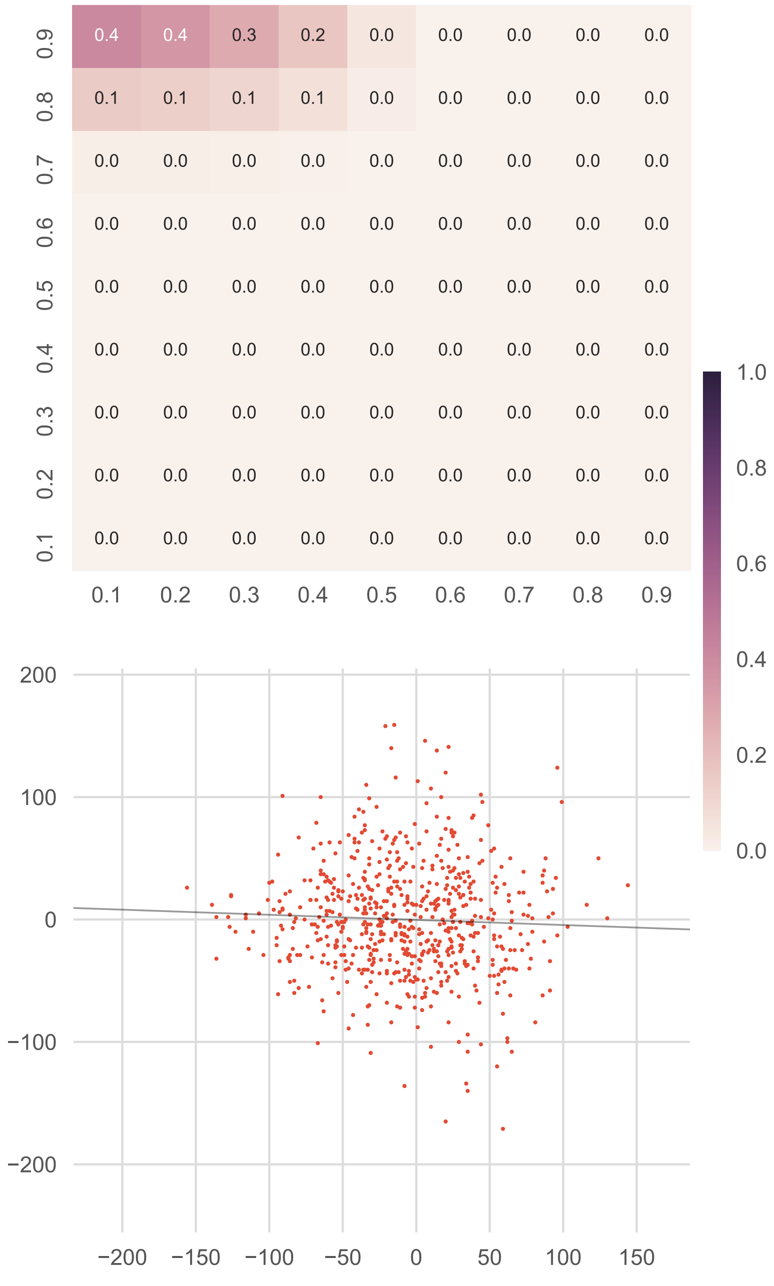



Figure 4: The fitted line was y = -0.316 + -0.042x. The Pearson Correlation Coefficient for the dataset was -0.044 with a p-value of 0.257. The Spearman Rank Correlation Coefficient for the dataset was -0.052. The Kendall Tau Rank Correlation Coefficient for the dataset was -0.035). The normalized mutual information content was 0.429.

## 5 Dataset-5

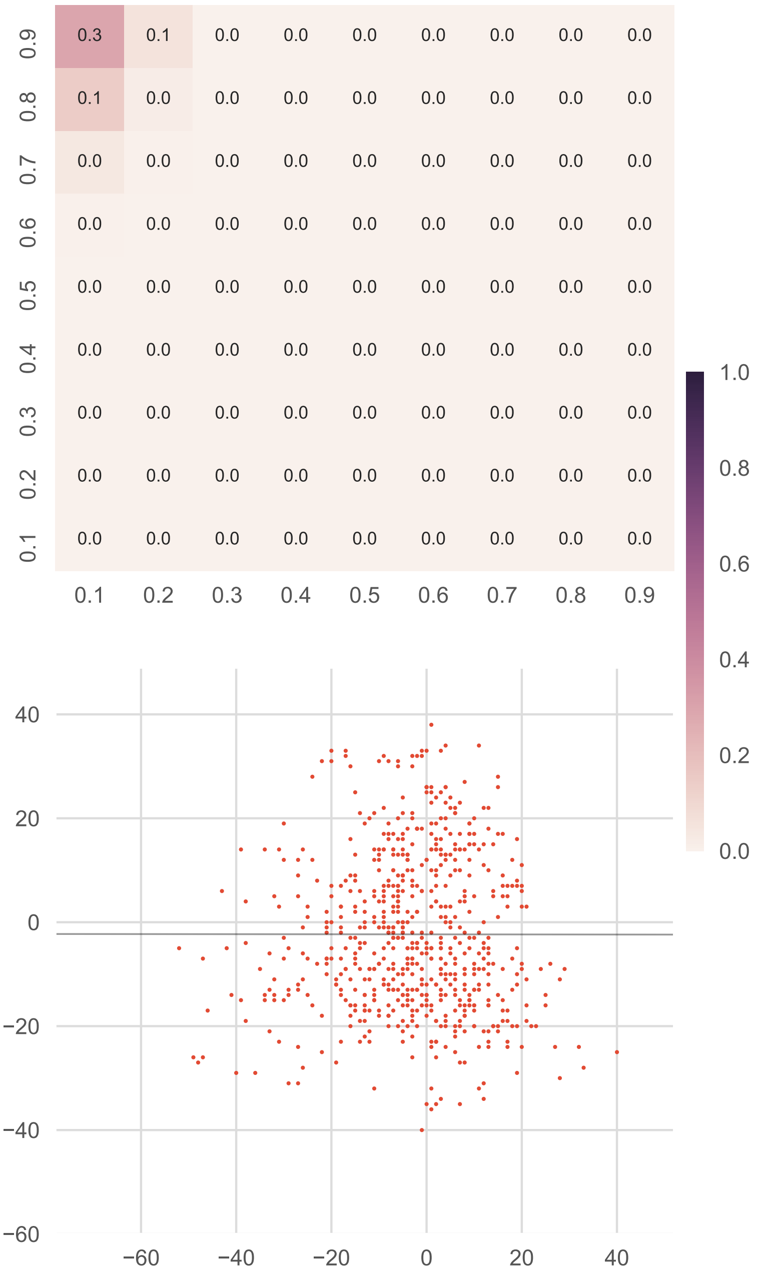




Figure 5: The fitted line was y = -2.335 + -0.001x. The Pearson Correlation Coefficient for the dataset was -0.001 with a p-value of 0.983. The Spearman Rank Correlation Coefficient for the dataset was -0.031. The Kendall Tau Rank Correlation Coefficient for the dataset was -0.019). The normalized mutual information content was 0.420.

## 6 Dataset-6

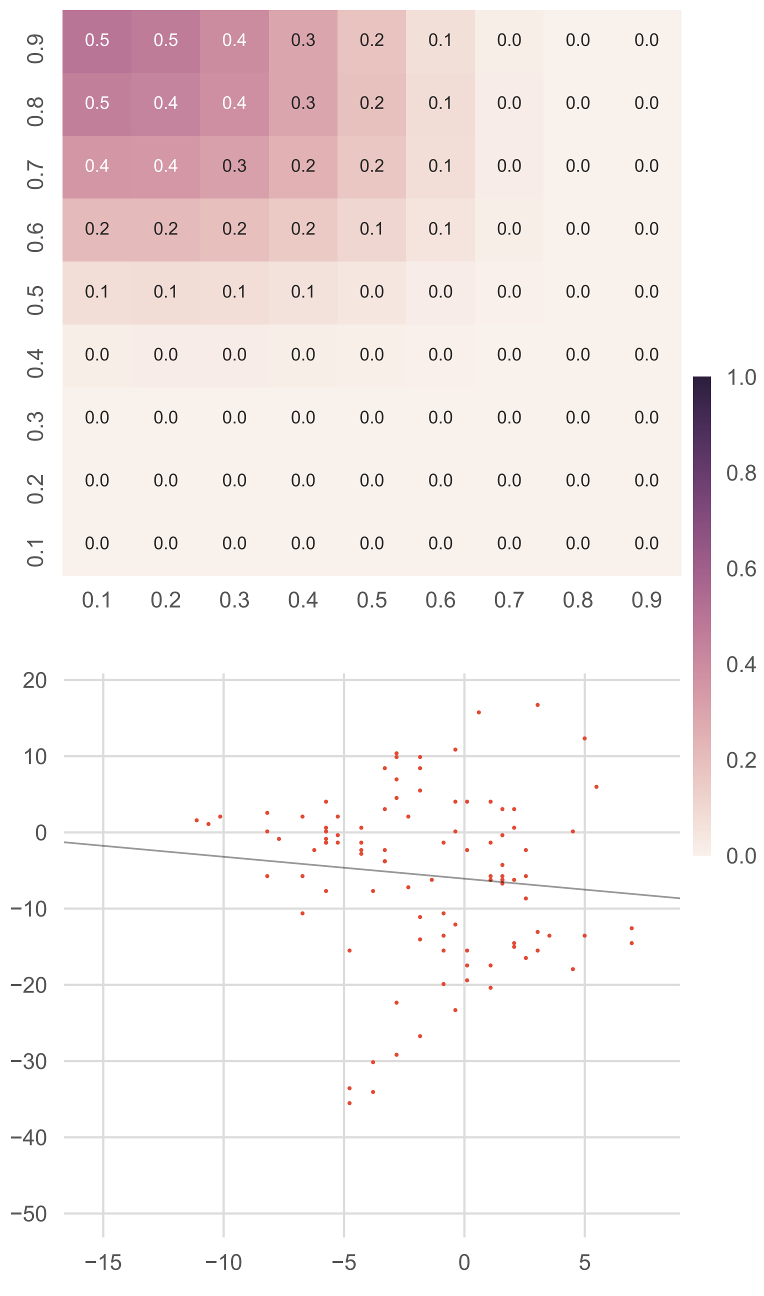


Figure 6: The fitted line was y = -6.073 + -0.287x. The Pearson Correlation Coefficient for the dataset was -0.103 with a p-value of 0.309. The Spearman Rank Correlation Coefficient for the dataset was -0.168. The Kendall Tau Rank Correlation Coefficient for the dataset was -0.110). The normalized mutual information content was 0.699.

# 7 Dataset-7

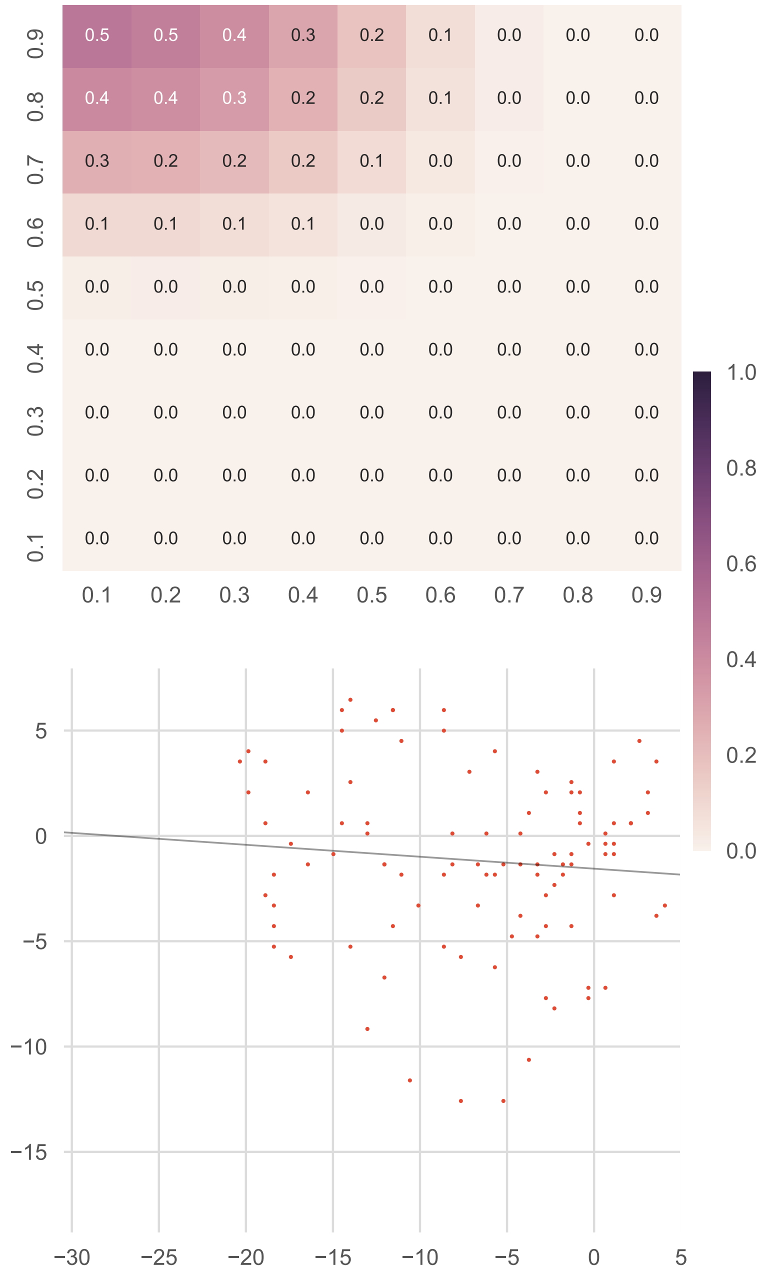




Figure 7: The fitted line was y = -1.557 + -0.057x. The Pearson Correlation Coefficient for the dataset was -0.092 with a p-value of 0.360. The Spearman Rank Correlation Coefficient for the dataset was -0.052. The Kendall Tau Rank Correlation Coefficient for the dataset was -0.020). The normalized mutual information content was 0.681.

# 8 Dataset-8

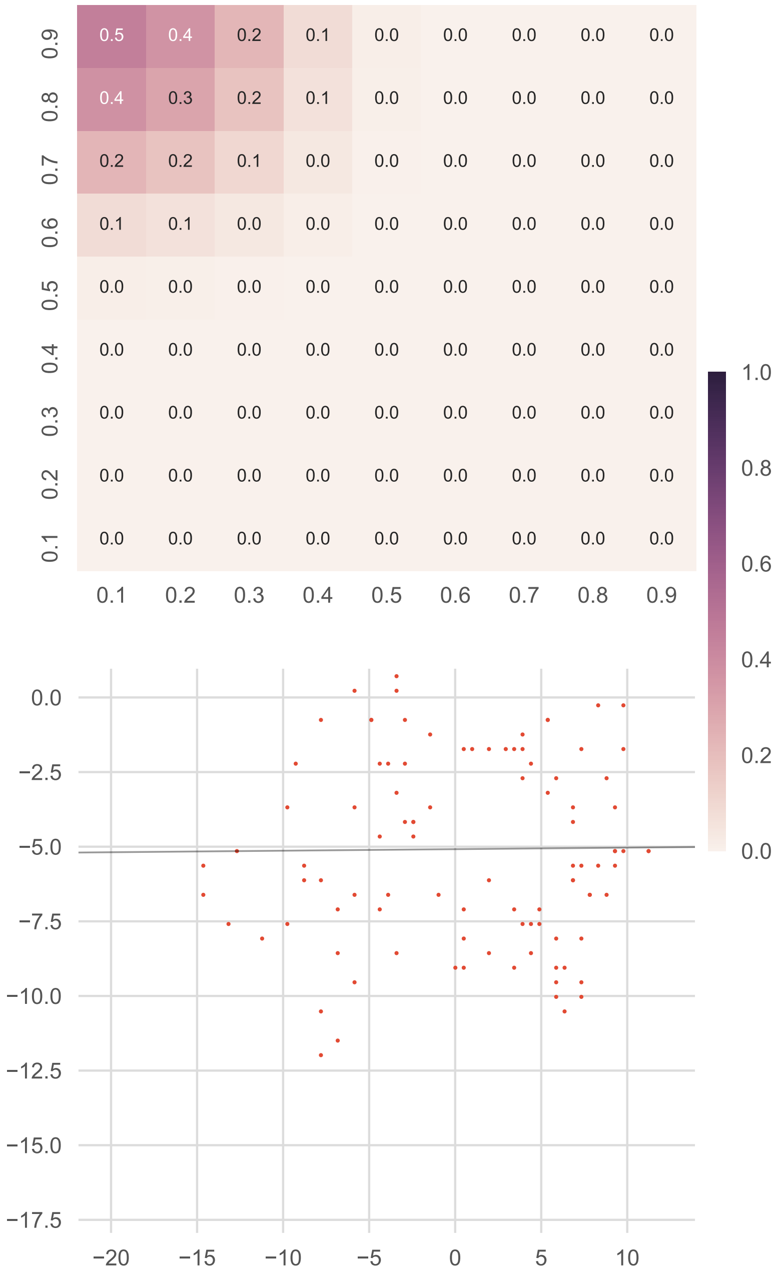



Figure 8: The fitted line was y = -5.083 + 0.005x. The Pearson Correlation Coefficient for the dataset was 0.011 with a p-value of 0.916. The Spearman Rank Correlation Coefficient for the dataset was 0.009. The Kendall Tau Rank Correlation Coefficient for the dataset was 0.005). The normalized mutual information content was 0.654.

# 9 Dataset-9

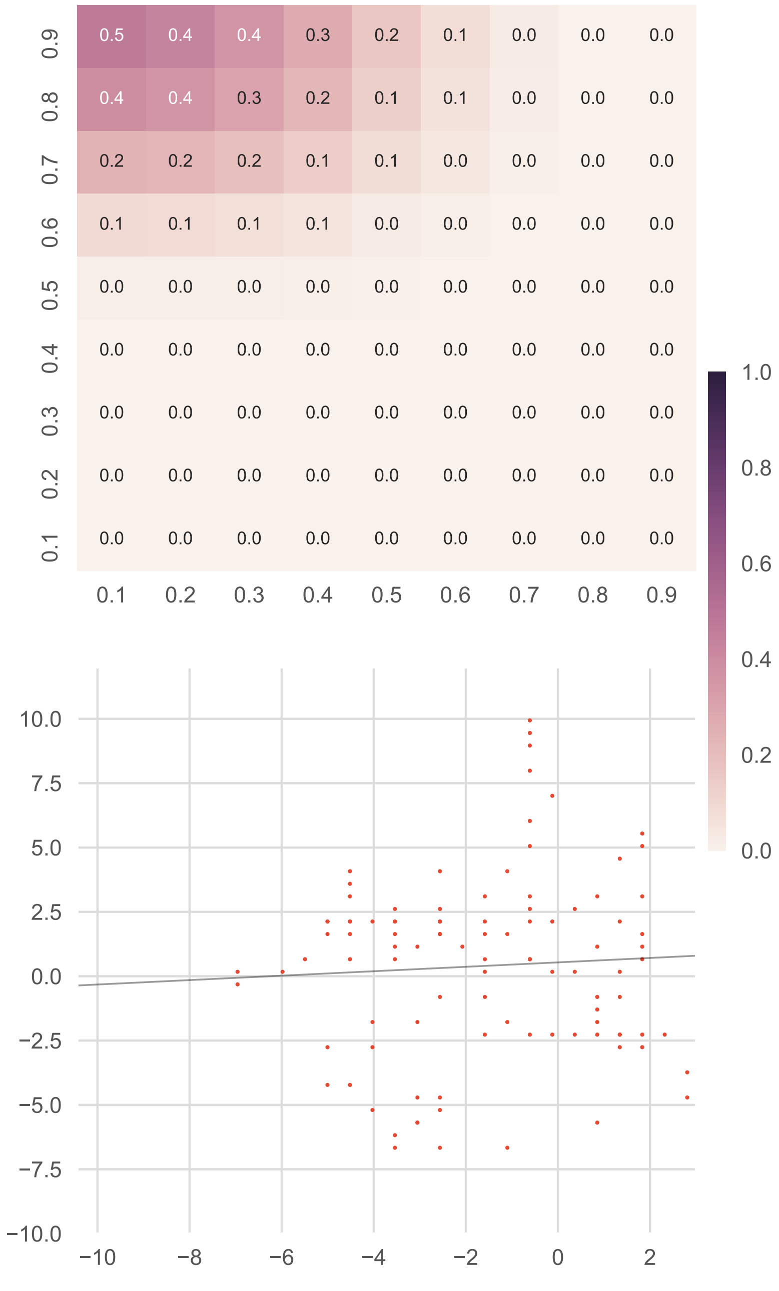




Figure 9: The fitted line was y = 0.538 + 0.086x. The Pearson Correlation Coefficient for the dataset was 0.058 with a p-value of 0.566. The Spearman Rank Correlation Coefficient for the dataset was 0.021. The Kendall Tau Rank Correlation Coefficient for the dataset was 0.010). The normalized mutual information content was 0.474.

## 10 Dataset-10

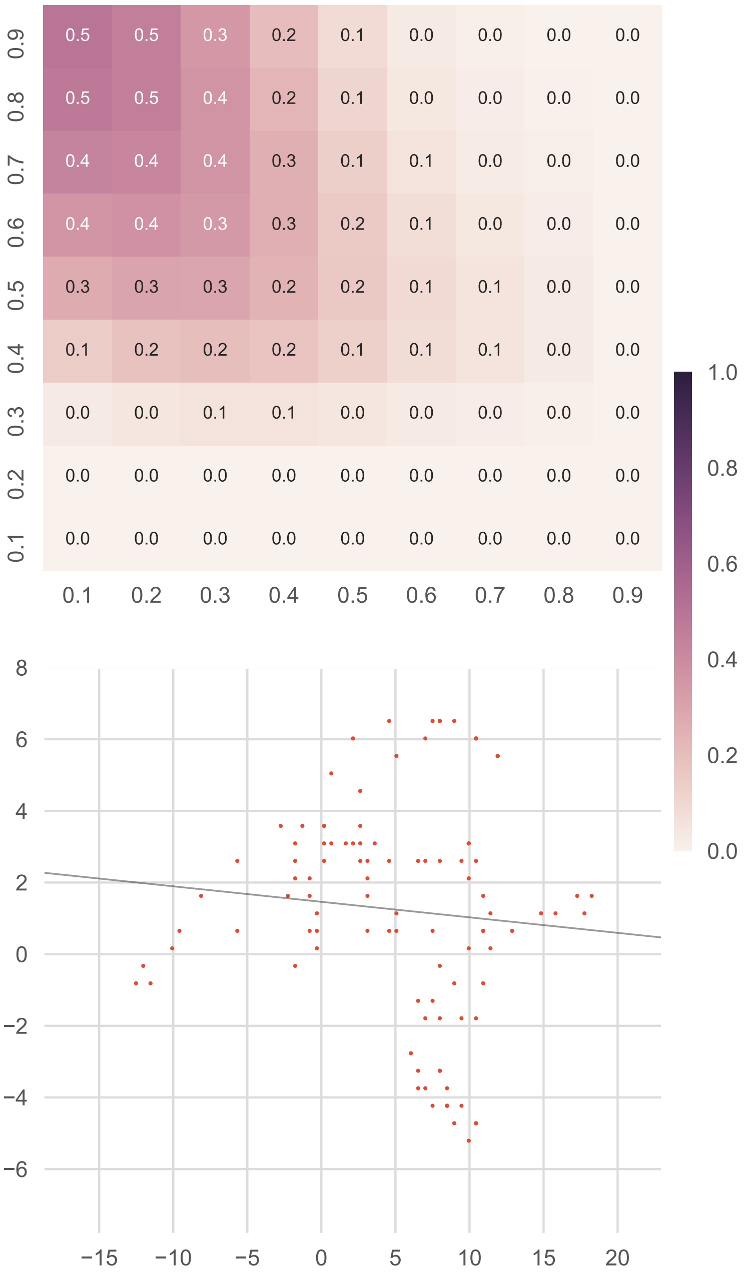




Figure 10: The fitted line was y = 1.461 + -0.043x. The Pearson Correlation Coefficient for the dataset was -0.091 with a p-value of 0.368. The Spearman Rank Correlation Coefficient for the dataset was -0.148. The Kendall Tau Rank Correlation Coefficient for the dataset was -0.088). The normalized mutual information content was 0.618.

# 11 Dataset-11

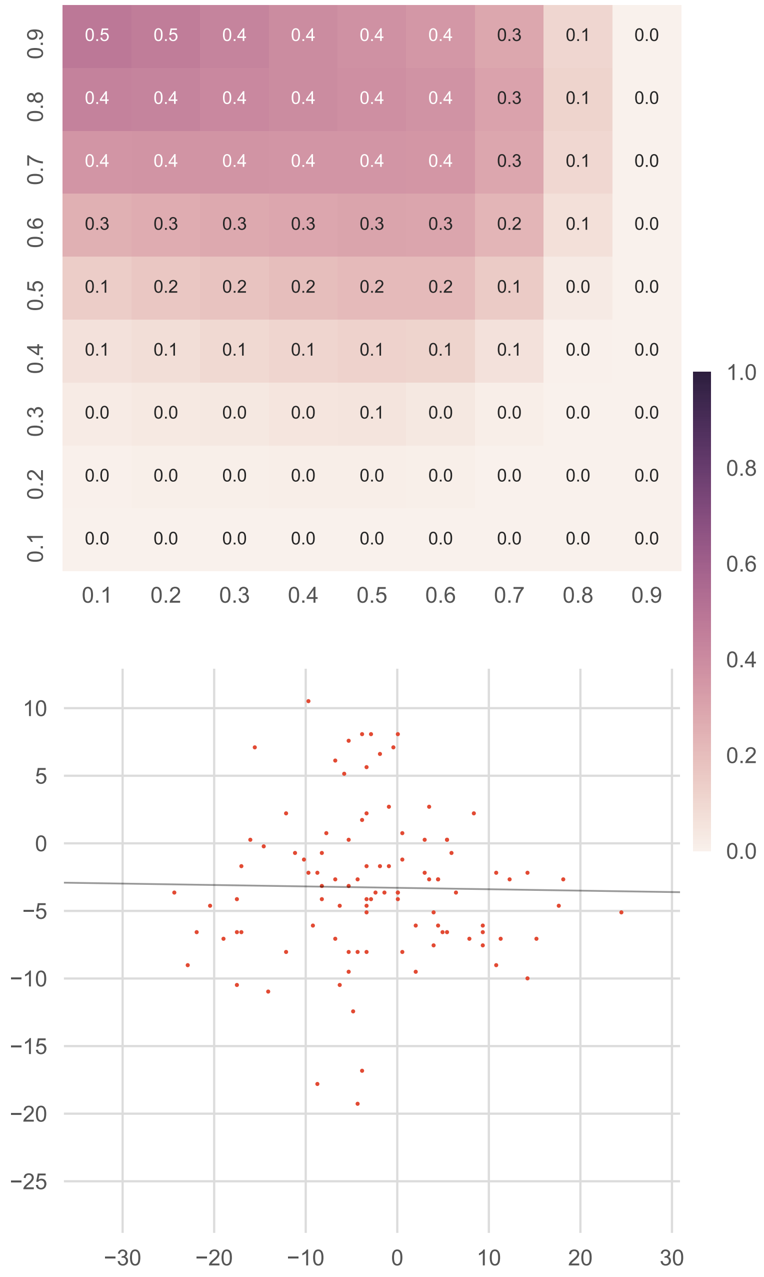


Figure 11: The fitted line was y = -3.291 + -0.010x. The Pearson Correlation Coefficient for the dataset was -0.018 with a p-value of 0.857. The Spearman Rank Correlation Coefficient for the dataset was -0.014. The Kendall Tau Rank Correlation Coefficient for the dataset was -0.018). The normalized mutual information content was 0.701.

## 12 Dataset-12

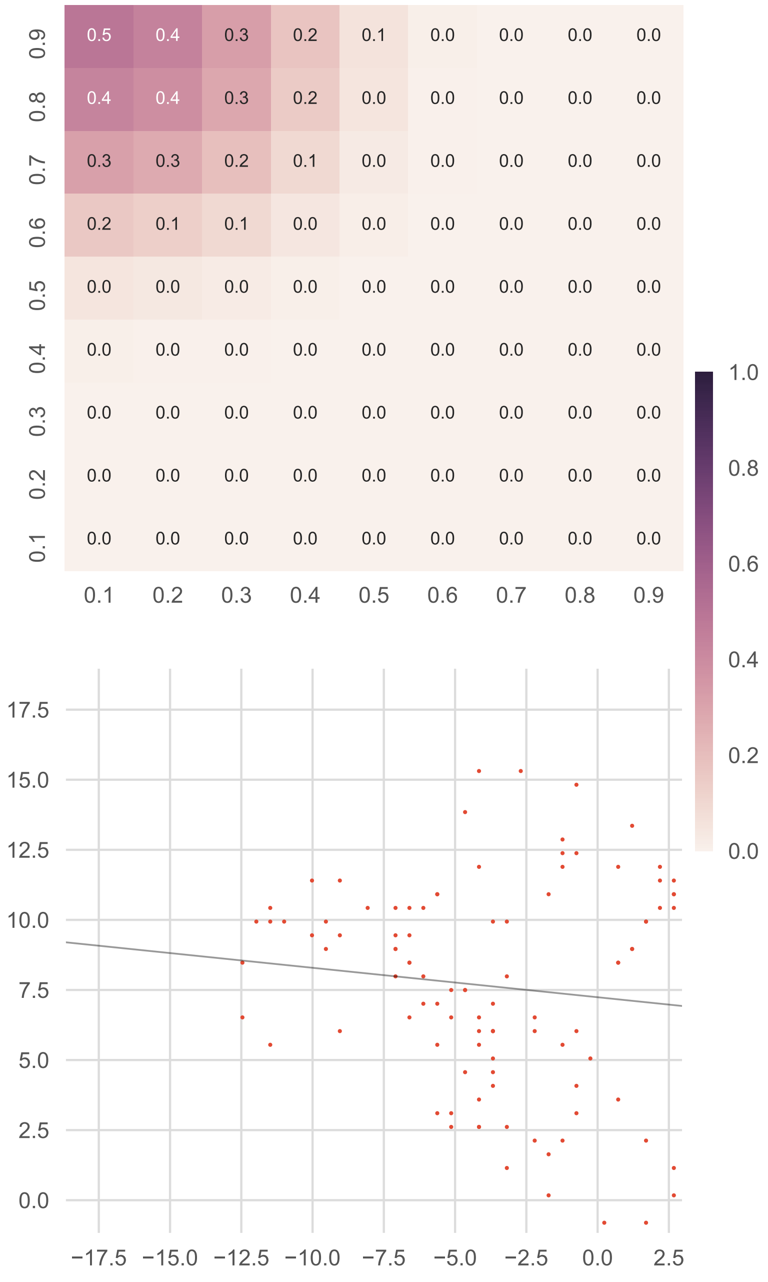




Figure 12: The fitted line was y = 7.241 + -0.105x. The Pearson Correlation Coefficient for the dataset was -0.113 with a p-value of 0.263. The Spearman Rank Correlation Coefficient for the dataset was -0.063. The Kendall Tau Rank Correlation Coefficient for the dataset was -0.083). The normalized mutual information content was 0.630.

# 13 Dataset-13

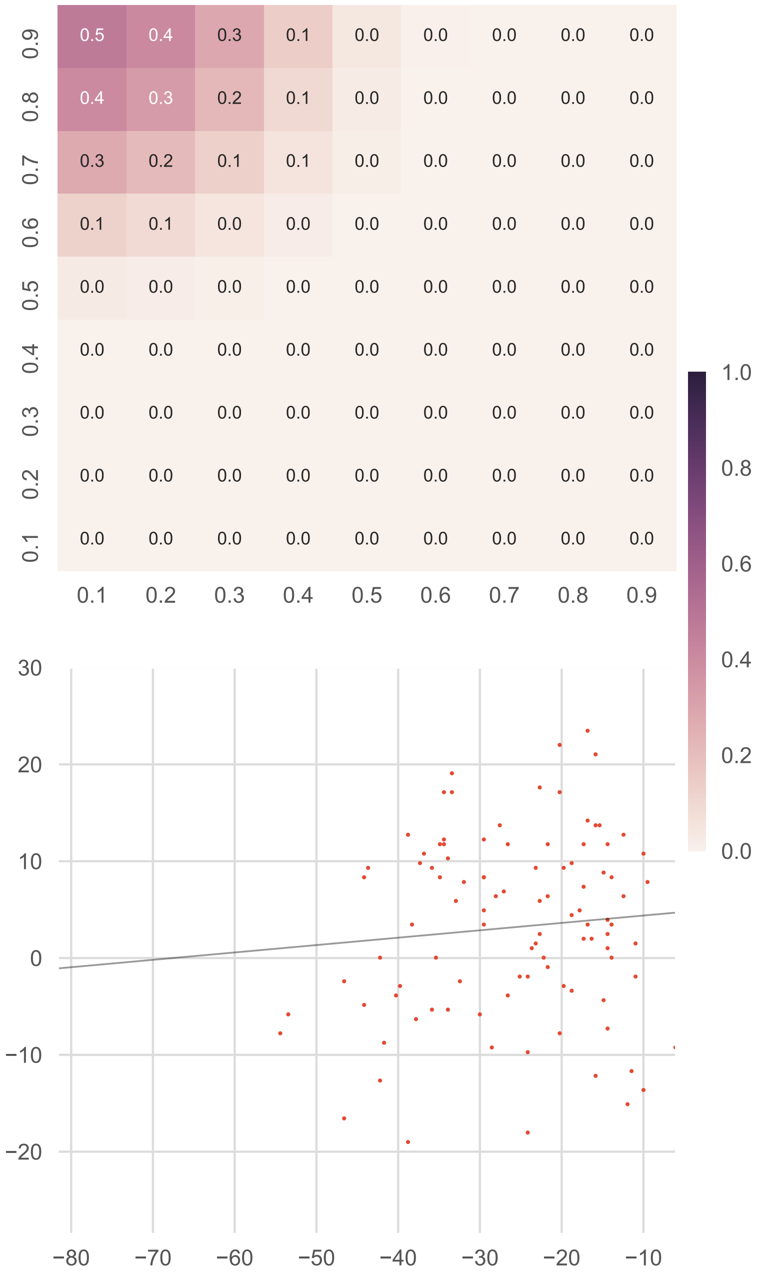




Figure 13: The fitted line was y = 5.152 + 0.076x. The Pearson Correlation Coefficient for the dataset was 0.090 with a p-value of 0.375. The Spearman Rank Correlation Coefficient for the dataset was 0.051. The Kendall Tau Rank Correlation Coefficient for the dataset was 0.035). The normalized mutual information content was 0.817.

## 14 Dataset-14

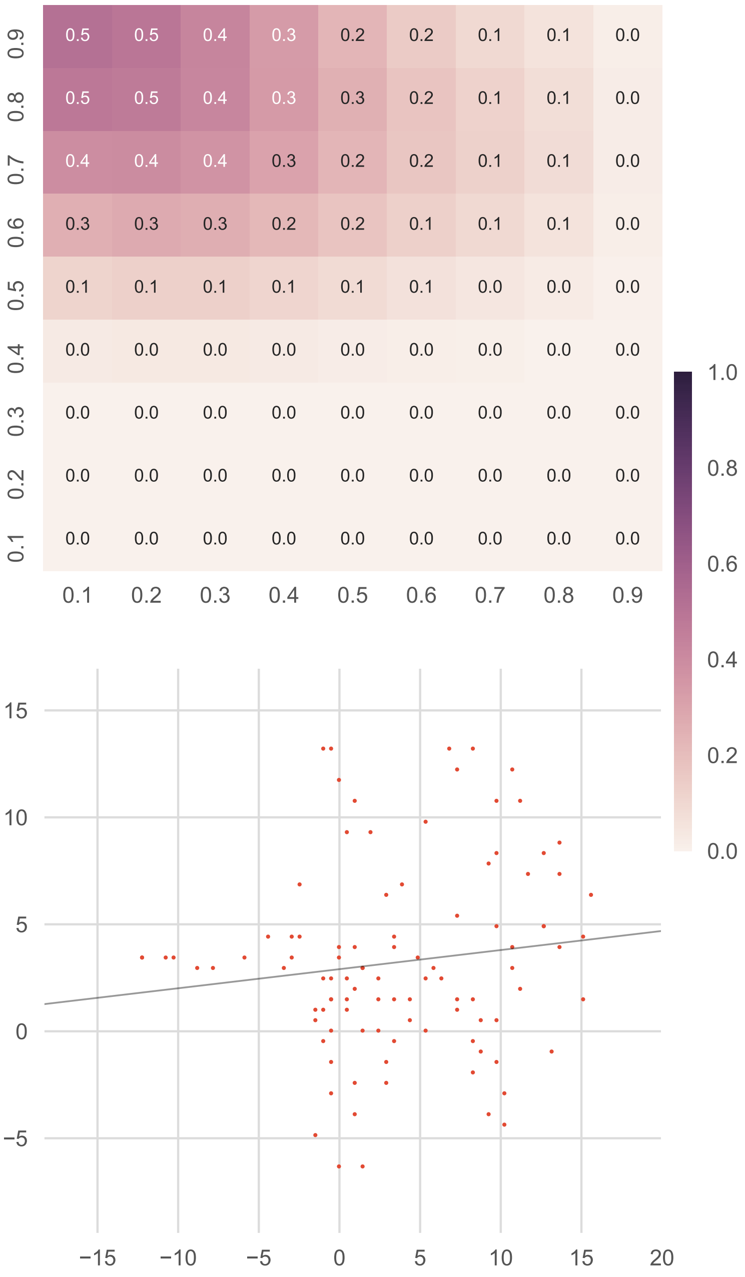




Figure 14: The fitted line was y = 2.904 + 0.089x. The Pearson Correlation Coefficient for the dataset was 0.126 with a p-value of 0.210. The Spearman Rank Correlation Coefficient for the dataset was 0.116. The Kendall Tau Rank Correlation Coefficient for the dataset was 0.061). The normalized mutual information content was 0.678.

## 15 Dataset-15

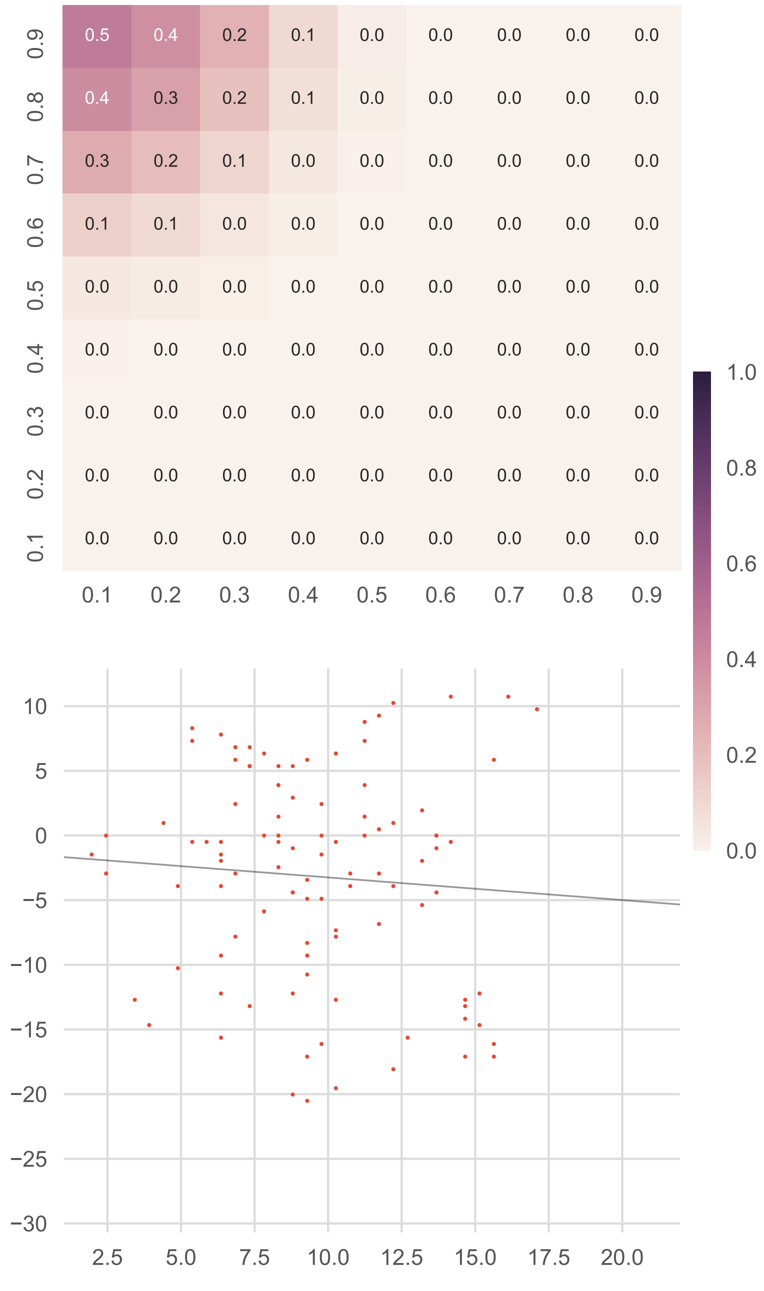


Figure 15: The fitted line was y = -1.495 + -0.175x. The Pearson Correlation Coefficient for the dataset was -0.072 with a p-value of 0.474. The Spearman Rank Correlation Coefficient for the dataset was -0.074. The Kendall Tau Rank Correlation Coefficient for the dataset was -0.054). The normalized mutual information content was 0.691.
